# *Bdellovibrio bacteriovorus* uses chimeric fibre proteins to recognize and invade a broad range of bacterial hosts

**DOI:** 10.1038/s41564-023-01552-2

**Published:** 2024-01-04

**Authors:** Simon G. Caulton, Carey Lambert, Jess Tyson, Paul Radford, Asmaa Al-Bayati, Samuel Greenwood, Emma J. Banks, Callum Clark, Rob Till, Elisabete Pires, R. Elizabeth Sockett, Andrew L. Lovering

**Affiliations:** 1 School of Biosciences, University of Birmingham, Birmingham, UK; 2 School of Life Sciences, University of Nottingham, Medical School, Queen’s Medical Centre, Nottingham, UK; 3 Chemistry Research Laboratory, University of Oxford, Oxford, UK; 4 Present Address: Biodiscovery Institute, School of Life Sciences, Nottingham University, Nottingham, UK; 5 Present Address: Northern Technical University, Kirkuk, Iraq; 6 Present Address: Department of Molecular Microbiology, John Innes Centre, Norwich, UK; 7 Present Address: Institute of Microbiology and Infection, University of Birmingham, Birmingham, UK

**Keywords:** Bacteriology, X-ray crystallography

## Abstract

Predatory bacteria, like the model endoperiplasmic bacterium *Bdellovibrio bacteriovorus*, show several adaptations relevant to their requirements for locating, entering and killing other bacteria. The mechanisms underlying prey recognition and handling remain obscure. Here we use complementary genetic, microscopic and structural methods to address this deficit. During invasion, the *B. bacteriovorus* protein CpoB concentrates into a vesicular compartment that is deposited into the prey periplasm. Proteomic and structural analyses of vesicle contents reveal several fibre-like proteins, which we name the mosaic adhesive trimer (MAT) superfamily, and show localization on the predator surface before prey encounter. These dynamic proteins indicate a variety of binding capabilities, and we confirm that one MAT member shows specificity for surface glycans from a particular prey. Our study shows that the *B. bacteriovorus* MAT protein repertoire enables a broad means for the recognition and handling of diverse prey epitopes encountered during bacterial predation and invasion.

## Abstract (web summary)

A diverse arsenal of fibre-like proteins extend outward from the *B. bacteriovorus* cell surface and localize towards prey cells, facilitating recognition and invasion.

## Main

*Bdellovibrio bacteriovorus* is a bacterium that preys upon a wide range of other Gram-negative bacteria, entering their periplasm and consuming them from within. Physical contacts between *B. bacteriovorus* and diverse prey bacterial surfaces precede a decision-making stage in which the predator commits to invasion^1–4^. The invasion process requires interaction with, and traversal through, layers of prey lipopolysaccharide and peptidoglycan, requiring several different, transient, prey-adhesive steps. Contact regions between *B. bacteriovorus* and prey are apparent in both electron microscopy^5^ and tomography studies^6^, and proteinaceous adhesins would need to extend beyond predator lipopolysaccharide and be diverse enough to correspond to the breadth of molecular envelope diversity in known prey strains. The recognition of bacterial surfaces by viruses and the immune system is well documented, but the means by which bacterial predators recognize prey and engage in envelope traversal without causing lysis has remained obscure until now.

We found serendipitously that *B. bacteriovorus* Bd0635–mCherry (a homologue of CpoB, a PBP1b regulator in non-predatory bacteria^7^) moves dynamically inside invading predators, concentrating to a focus that develops into a discrete vesicular compartment that is deposited by the predator into the periphery of the prey periplasm, and persists in debris after predator exit. Analysis of vesicle contents reveals a family of phage-tail-fibre-like proteins with diverse adhesin domains.

These fibre-like proteins form discrete foci on attack-phase predators when invading prey, disappearing from external detection after predator invasion into prey, moving to a focus inside the prey periphery and co-locating with CpoB_Bd0635_ for several family members. Structural studies reveal a variety of domain usages (and therefore binding capabilities) at the fibre C-termini, placing these on intertwined trimeric β-prisms. We infer that this large mosaic adhesive trimer (MAT) protein repertoire allows predator interactions with, and invasion of, a diversity of bacterial prey with different cell envelopes and surfaces. We confirm binding of one MAT type to multiple bacterial surface glycans including those of *Proteus mirabilis*.

## Results

### *B. bacteriovorus* deposits a predator-derived vesicle inside prey

**Fig. 1:**
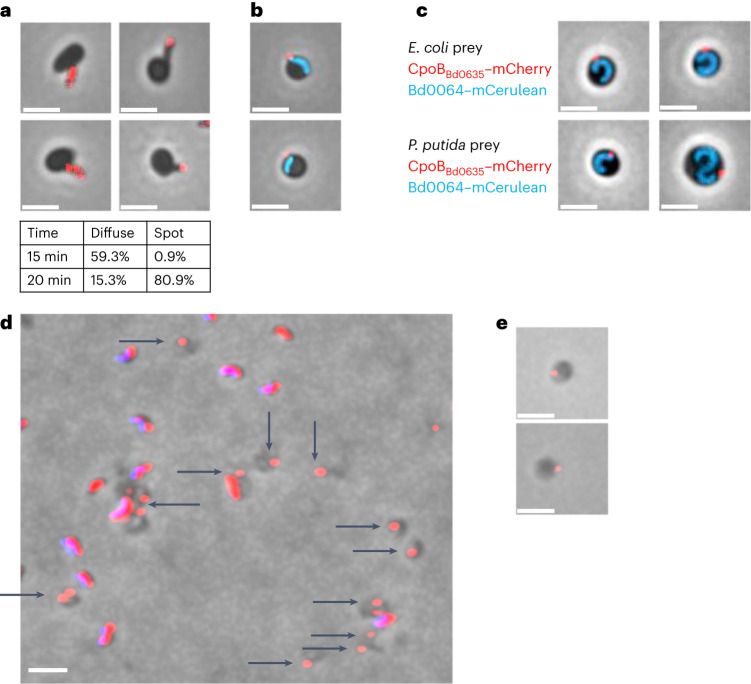
*B. bacteriovorus* lays down a robust vesicle ‘invasion organelle’ during invasion. **a**, Phase and epifluorescence microscopy tracking the movement of CpoB_Bd0635_–mCherry during *B. bacteriovorus* attachment to, and entry into, prey. At earlier time points, most cells exhibit a diffuse fluorescent signal throughout the cell. The table shows that the fluorescence moves to become a distinct focus at the distal, flagellated pole of *B. bacteriovorus* as they begin to enter the prey cell. **b**,**c**, Phase and epifluorescence microscope images showing a fluorescent CpoB_Bd0635_–mCherry focus (red) at the periphery of the prey cell, proximal to the cytoplasmic mCerulean of the predator cell (blue) at 30 min (**b**) and distal to the cytoplasmic mCerulean of the predator cell (blue) at 120–180 min (**c**) after the mixing of the predator and prey. This is shown when the prey is either *E. coli* or *Pseudomonas putida*. **d**, Phase-contrast and epifluorescence microscope images showing deposited vesicles remaining among prey envelope debris after prey exit: attack-phase *B. bacteriovorus* HD100 with CpoB_Bd0635_–mCherry fluorescence (red) alongside DAPI predator genome fluorescence (blue—resulting in a pink merge) and broken prey bdelloplast debris lacking DAPI signal (dark grey), but in cases containing CpoB_Bd0635_–mCherry fluorescent foci (black arrows). **e**, Phase and epifluorescence microscopy showing CpoB_Bd0635_–mCherry fluorescent (red) foci left in the spherical ‘ghost’ prey bdelloplast peptidoglycan sphere (dark grey), which is specifically left behind, unbroken, after predation on *E. coli* by the mutant *B. bacteriovorus* Δ*bd0468Δbd3279*. The images are representative of three independent repeats, and scale bars are 2 µm.

C-terminally mCherry-tagged CpoB_Bd0635_ (Supplementary Fig. 1) was initially diffuse throughout predator cells but became focused near the flagellar pole of *B. bacteriovorus* as they invaded *Escherichia coli* prey (Fig. 1a and Supplementary Figs. 2–4). The mCherry focus was proximal to the trailing predator pole within the rounded prey ‘bdelloplast’ during early predation (Fig. 1b) but was detached from predator cells after 120–180 min of predator growth. At this later stage, the CpoB_Bd0635_–mCherry-containing vesicle could be seen still within the periphery of bdelloplasts, but more clearly distinct from the *B. bacteriovorus* growing filamentous cell (Fig. 1c). It persisted robustly after prey exit, remaining fluorescent and attached to broken pieces of prey envelope debris, for wild-type predators (Fig. 1d), or persisting in prey cell ghost envelopes remaining after exit by mutant *Δbd0468Δbd3279* double deacetylase predators (Fig. 1e)^8^. Deletion tests for *bd0635* (refs. ^2,9^) resulted only in wild-type revertants, even when selecting for host-independent isolates, suggesting that CpoB_Bd0635_ may be essential for viability in *B. bacteriovorus*.

Vesicle CpoB_Bd0635_–mCherry fluorescence and the HADA-labelled entry porthole in prey peptidoglycan^1^ were coincident (Supplementary Figs. 4–7). HADA, 3-[[(7-hydroxy-2-oxo-2H-1-benzopyran-3-yl)carbonyl]amino]-D-alanine hydrocholoride. Together, these results identify a robust vesicle that is produced by predators during prey entry and remains associated with the point of invasion in the bdelloplast.

### Analysis of the vesicle reveals several phage-like proteins

Proteomic analysis of enriched CpoB_Bd0635_–mCherry vesicle preparations (Supplementary Figs. 8 and 9, and ‘Methods’ in Supplementary Note 1) revealed a group of homologous proteins annotated as ‘cell wall anchor/YapH-like/phage-related tail fibre’ proteins (Supplementary Table 1). As discussed below, these could be grouped into two subfamilies, S74 and non-S74 (NS74), depending on the presence of an autoproteolytic S74 protease and chaperone domain^10^, with 13 and 8 members, respectively, identified in the *B. bacteriovorus* genome. For reasons explained later, we group these as the MAT family, for ‘mosaic adhesive trimeric’ fibre.

### Several MAT proteins locate to the vesicle or bdelloplast

**Fig. 2:**
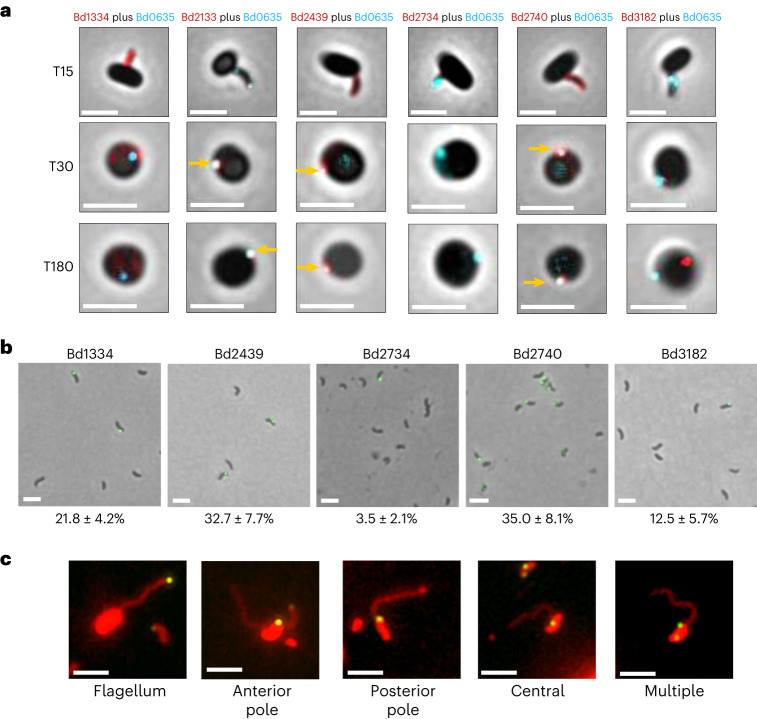
MAT protein localization on predators and in prey. **a**, Composite phase and epifluorescence microscope images showing attachment to, and growth of, predator strains inside *E. coli* prey bdelloplasts. The epifluorescence signal is from the C-terminally fluorescently tagged proteins: Bd1334–mCherry with CpoB_Bd0635_–mTeal, Bd2133–mNeonGreen with CpoB_Bd0635_–mCherry, Bd2439–mCherry with CpoB_Bd0635_–mTeal, Bd2734–mTeal with CpoB_Bd0635_–mCherry, Bd2740–mCherry with CpoB_Bd0635_–mTeal and Bd3182–mNeonGreen with CpoB_Bd0635_–mCherry. For comparison purposes, false colour is used in all images: CpoB_Bd0635_ is coloured cyan and all MAT proteins are coloured red; coincident double fluorescence is shown in white. Representative images from a full predatory time course for each strain are presented in Supplementary Fig. 11. For Bd2133, Bd2439 and Bd2740, coincidence with C-terminally fluorescently tagged CpoB_Bd0635_ is observed as a white focus (vesicle) indicated by yellow arrows. T15, T30 and T180 are 15 min, 30 min and 180 min, respectively, that have elapsed since predators and prey were mixed. Scale bars are 2 µm. The images are representative of three biological repeats. **b**, Composite phase and epifluorescence images showing spots of immunofluorescence on the outer surface of *B. bacteriovorus* attack-phase cells using FITC anti-mCherry antiserum and C-terminally mCherry-tagged MAT proteins. Bd2133 is absent from this analysis as a C-terminal mCherry tag was not tolerated by this protein. Details of immunofluorescent spot numbers and locations at different stages of predation are presented in Supplementary Fig. 13. The percentage of cells with observable immunofluorescent spots is presented for each strain below the image. Scale bars are 2 µm. The images are representative of cells from at least three biological repeats. **c**, Composite epifluorescence microscope images of FITC-labelled anti-mCherry antiserum immunofluorescence combined with the red membrane stain FM46-4 on attack-phase *B. bacteriovorus* cells reveal the locations of immunofluorescent spots with respect to the flagellum across all the mCherry-tagged MAT proteins examined. Details of the frequency of location for the different proteins are presented in Supplementary Fig. 13b. Scale bars are 2 µm. The images are representative of three biological repeats.

From this MAT grouping, we sampled several proteins for in vivo fluorescence tag localization. Bd2133 and Bd2439 (from the vesicle enrichment proteome) were deposited within the same vesicle as CpoB, while Bd3182 was deposited elsewhere within the bdelloplast and only at later time points (Fig. 2a; Supplementary Figs. 11, 12 and 15; and Supplementary Note 2).

MAT NS74 proteins Bd1334, Bd2734 and Bd2740 were studied as they represented different subgroupings, with potentially different functionalities (discussed later), but had not been found in the proteome of the vesicle (which was collected after predation). We found that Bd2740–mCherry did locate to the CpoB_Bd0635_-containing vesicle, deposited during predatory invasion, while Bd1334–mCherry and Bd2734–mCherry fluorescence were diffuse throughout the *B. bacteriovorus* cell (Fig. 2a, and Supplementary Figs. 11 and 12).

### MAT proteins are surface displayed on predators

We hypothesized that some MAT proteins may present their C-termini out of the *B. bacteriovorus* cell for prey interactions. Testing with a fluorescent secondary antibody and anti-mCherry antiserum revealed that all C-terminally tagged MAT proteins tested were detected at various locations on the surface of the attack-phase *B. bacteriovorus* cell, as expected for prey-interacting fibres, but were not detected externally after predator entry into the bdelloplast (Fig. 2b,c, Supplementary Figs. 10 and 12–16, and Supplementary Note 2).

**Fig. 3:**
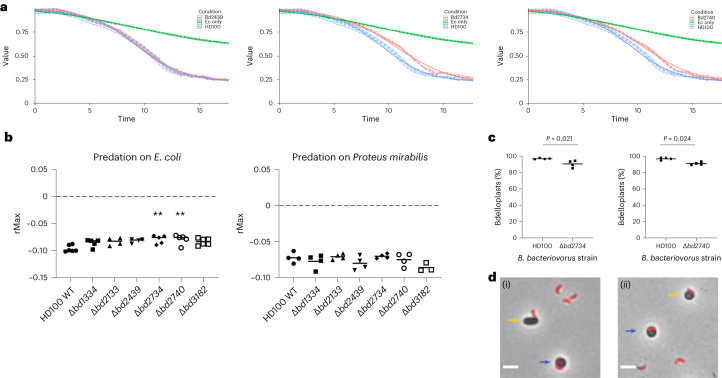
Predation assays for MAT mutant *B. bacteriovorus*. **a**, Monitoring reductions in prey cell OD_600_ as a result of prey lysis by predator MAT mutants (red) relative to the wild type (blue) versus prey *E. coli* only without predators (Ec) (green). Examples shown are predation by *Δbd2439*, which has no predatory defect, and Δ*bd2734* and Δ*bd2740*, which are delayed in predation relative to the wild type. **b**, Measures of the rMax for all mutant strains. rMax is significantly higher (a lower rate of OD_600_ reduction and therefore predation) for Δ*bd2734* and Δ*bd2740* when preying upon *E. coli* (Kruskal–Wallis test on non-normally distributed data, **P < 0.01; *P* = 0.0099 for Δ*bd2734* and *P* = 0.005 for Δ*bd2740*), but not significantly different for any strains preying upon *Proteus mirabilis* (one-way ANOVA on normally distributed data). Values of *n* (independent biological repeats) are, for predation on *E. coli*, 4 (Δ*bd2133*, Δ*bd2439*), 5 (Δ*bd1334*, Δ*bd2734*, Δ*bd2740*) and 6 (HD100, Δ*bd3182*) and, for predation on *Proteus mirabilis*, 3 (Δ*bd3182*) and 4 (all others). **c**, Percentage of bdelloplasts visually scored for a fully entered *B. bacteriovorus* 30 min after the mixing of the predator and prey for *B. bacteriovorus* HD100 wild type versus MAT deletion mutant strains Δ*bd2734* and Δ*bd2740*. The *P* values presented are from two-tailed unpaired *t*-test. Data are from four biological repeats. Details in Supplementary Fig. 17b. **d**, Example regions of interest showing the diversity of cells characterized as fully entered and incomplete for *B. bacteriovorus* MAT mutant entry. Cells counted as incomplete entry included rod-shaped prey with *B. bacteriovorus* attached (i) and rounded prey with partially entered *B. bacteriovorus* (ii) as indicated by yellow arrows. Blue arrows indicate cells characterized as *B. bacteriovorus* having fully entered the periplasm of the prey. Scale bars are 2 µm.

We deleted each individual gene for the six sampled proteins, finding a small, but significant, predation defect in two of the (NS74) mutants: Δ*bd2734* and Δ*bd2740*, using a microtitre-plate-based predation assay^11^ (Fig. 3). That the effect was small, and not seen for all MAT mutants (Supplementary Fig. 17), was expected as a multifactorial recognition and handling of the prey cell surface occurs during *B. bacteriovorus* predation. The rate of predation (rMax) on *E. coli* S17-1 prey was reduced with mutants Δ*bd2734* and Δ*bd2740*, but predation on an alternative prey, *Proteus mirabilis*, was not significantly different for any MAT mutant compared with the wild type (Fig. 3b). The reduced rMax may be a result of delayed attachment to, and entry into, prey; this is supported by our observation that detectibly fewer prey had been entered by the Δ*bd2734* (90.5% ± −4%) and Δ*bd2740* (91.1% ± −1.6%) mutants 30 min after mixing compared with the wild type (WT for Δ*bd2734*, 96.7% ± −0.7%, and WT for Δ*bd2740*, 96.7% ± −1.4%; Fig. 3c).

### Characterization of the wider MAT family and S74 activity

Having found MAT members on the surface of predators and some in the vesicle proteome, we sought to identify the full complement, and their structural diversity, in *B. bacteriovorus* HD100. Using Position Specific Iterative BLAST^12^, manual inspection and fold prediction^13^, we were able to find 21 confident matches (Supplementary Table 1), 13 terminating in a consensus S74 peptidase domain with a SDxxxK catalytic motif^10^ and 8 NS74 MATs that do not.

The S74 protein domain is a dual chaperone and protease domain shown to confer quality control, ensuring productive trimerization in surface-exposed phage β-helical spikes and enzymes^14^. All 21 MATs contain a conserved β-rich domain of ~130 aa immediately following the signal peptide.

**Fig. 4:**
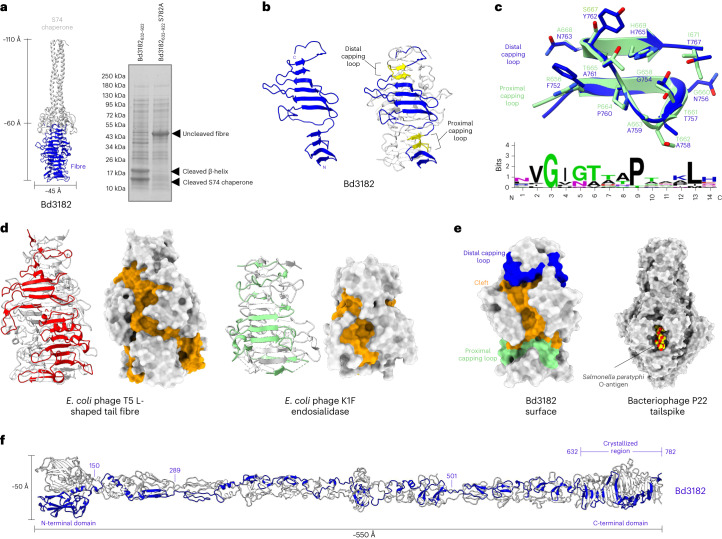
Structure and features of MAT protein Bd3182. **a**, ColabFold model of Bd3182 β-helix and S74 chaperone. The accompanying SDS-PAGE gel shows classical S74 processing of Bd3182 into separate fibre and chaperone domains, which is blocked in the S782A mutant. This autoproteolysis was observed on more than four independently expressed samples of Bd3182. **b**, Crystal structure of Bd3182_632–782_ (hereafter referred to as Bd3182). The single chain (left) forms a symmetric trimer (right). Distinctive motifs form capping loops on the β-helix edges (yellow). **c**, The capping loops form similar structures exemplified by superposition of the proximal (green) and distal (blue) loops of Bd3182. An alignment of capping loops from multiple fibres (Supplementary Fig. 18) provides a consensus motif for this feature (bottom). **d**, The phage T5 L-shaped tail fibre (red; PDB code 4UW8 (ref. ^14^)) and K1F endosialidase (green; PDB code 3GW6 (ref. ^10^)) show similar β-helical structures to that of Bd3182. The surface of each features a cleft formed by the beta sheet of the fibre (orange surface). **e**, The surface of Bd3182 shows a continuous cleft (orange), capped by the proximal (green) and distal (blue) capping loops. Bacteriophage P22 (structural homologue of Bd3182; main text) binding of O-antigen (carbon atoms, yellow) within a cleft on the surface of the β-helical fibre (PDB code 3TH0 (ref. ^50^)). **f**, Full-length fibre of Bd3182 modelled using a composite of ColabFold models and our experimentally determined crystal structure. Source data

A C-terminal region (amino acids 632–922) of Bd3182 confirmed the predicted chaperone and protease activity of the S74 domain, yielding the expected two bands, with self-cleavage at catalytic residue S782 (Fig. 4a). An S782A null mutant results in a single unprocessed species (Fig. 4a), in a manner identical to other S74-containing proteins^10^. Bd3182–mCherry prey bdelloplast location, sometimes as a tight focus, was seen at 120–180 min into predation (Supplementary Fig. 15) and was abolished in the S782A variant. Having potentially found a family of trimerizing adhesins with diverse adhesive tips facilitating predator–prey interactions, we set out to define their structures.

### Structure of a representative S74 group MAT fibre tip

We generated three different forms of the mature ‘tip’ region of family member Bd3182 (Fig. 4a and Supplementary Table 2; we describe the 1.1 Å form herein). The structure traces P647 to A781, forming a trimeric β-helix 40 Å across and 60 Å high (Fig. 4b). Each face of the β-helix is formed from multiple chains, resulting in an intertwined structure. Bd3182 retains the classical hydrophobic β-helix core, and the arrangement of loops on the surface creates a 30 Å × 15 Å cleft (Fig. 4b), limited in dimensions by two capping loops of identical structure (R656–H669 and F752–H765; Fig. 4b,c).

We found related loops in other *B. bacteriovorus* MATs (Supplementary Fig. 18) and generated a consensus motif (V–G–G/N–π–X–X–P–X–X–X–L; Fig. 4c). Searching the Protein Data Bank with this motif returns a hit to the S74-containing Myelin Regulatory Factor (MyRF)^15^, revealing an S74 feature conserved beyond the catalytic protease.

Trimer-to-trimer comparison reveals homology to bacteriophage S74 proteins, with Root-Mean-Square Deviations of 2.4 Å and 2.5 Å for phage K1F (3GW6 (ref. ^10^)) and bacteriophage T5 (4UW8 (ref. ^14^)), respectively. The RMSDs indicate that Bd3182 is a true S74 member, with a surface cleft similar to that of others (Fig. 4d,e), whose exposure (via S74 proteolysis) is necessary to bind their O-antigen ligand. The best match of a monomer via DALI^16^ analysis is to the podovirus P22 tailspike (RMSD 4.4 Å), whose bound O-antigen fragment^17^ is positioned in agreement with the Bd3182 cleft (Fig. 4e). An AlphaFold^18^ model of full-length Bd3182 reveals that trimerization is retained along its entirety, generating a fibre ~550 Å long (Fig. 4f and Supplementary Fig. 19). Thus, predatory bacterial MAT protein Bd3182 is related to phage adhesins but uses a distinctly longer fold with a more exposed binding cleft.

### Structures of NS74 members reveal MAT fibre-tip diversity

Taking Bd3182 as a representative of the other S74-terminating MATs, we next studied the eight more divergent ‘NS74’ proteins with differing termini. A region comprising N662 to the C-terminus of Bd2133 was determined to 2.5 Å resolution (Supplementary Table 2).

**Fig. 5:**
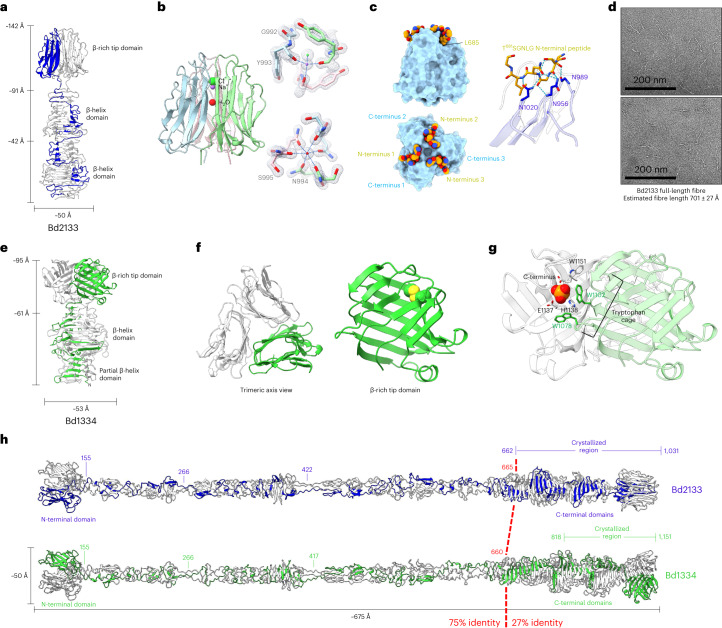
Structures of two divergent NS74 MAT proteins, Bd2133 and Bd1334. **a**, Crystal structure of Bd2133_662–1,031_ (hereafter referred to as Bd2133). This portion of the trimeric fibre forms a structure 142 Å in length and can be subdivided into three distinct domains: two β-helix domains and a C-terminal β-rich tip domain. **b**, The β-rich tip domain of Bd2133 forms a previously uncharacterized fold comprising a β-sandwich that packs into a trimer with multiple atoms and ions along the central three-fold axis (left). A stick representation of the coordination is shown (right), with a 1.5 Å 2fo–fc map at 2*σ*. **c**, The N-terminus of Bd2133 (residues T^681^SGNLG; orange) binds to the C-terminal domain in the crystal structure (left). The crystal symmetry provides a 1:1 stoichiometry of the N-terminal peptide to the trimeric C-terminal domain. The peptide binds through multiple backbone and side-chain hydrogen bonds, and packs L685 into a moderately hydrophobic shallow pocket. **d**, Bd2133_21–1,031_ full-length fibres show flexibility when imaged via negative-stain electron microscopy. These are representative micrographs from multiple grids made of a single sample of purified Bd2133. **e**, The crystal structure of Bd1334_818–1,151_ (hereafter referred to as Bd1334). This portion of the trimeric fibre forms a structure 95 Å in length, and the protein can be subdivided into a partial β-helix domain, a full β-helix domain and a C-terminal β-rich tip domain. **f**, The Bd1334 β-rich tip domain forms a previously uncharacterized fold comprising a squashed β-sandwich that packs into a trimer with the strands diagonal to the trimeric axis. A single disulphide is present (right, shown as spheres). **g**, A phosphate molecule (shown as spheres) is buried in a cavity formed between two protomers. Multiple residues interact with the phosphate, including W1078 and W1102, which form a cage around the ion. **h**, Full-length fibres of Bd2133 and Bd1334 modelled using a composite of ColabFold^13^ models and our experimentally determined crystal structures. The two proteins comprise very similar N-termini, both in sequence (75% sequence ID) and predicted structure; however, the C-terminus has been exchanged at some point during evolution.

The Bd2133 tip is 150 Å long, differentiated into three subdomains (Fig. 5a)—two β-helix regions akin to Bd3182 (G683–E813 and N814–A907) and a β-sandwich domain that substitutes for the S74 chaperone (W913–K1031). The latter two domains are linked by a stalk-like linear peptide (A^906^AGAAT). Despite a general agreement in the β-helix region, Bd2133 does not possess a Bd3182/S74-like cleft. Equivalents to the Bd3182 capping loops are present, demonstrative of common principles in the S74 and NS74 subgroupings.

The β-sandwich tip domain of Bd2133 adopts a twisted fold, trimerizing about strand G989–W999 (burying 5,640 Å^2^ per monomer). There are several atoms lying on the three-fold axis (Fig. 5b). Solving a 1 Å tip-only variant (A910 onwards) allowed us to confidently model these as a water, a sodium and a chloride ion. Unexpectedly, there are no homologues of this domain in the PDB (judged by DALI^16^), but FoldSeek^19^ found strong homology to Synechococcaceae and *Competibacter* proteins (Supplementary Fig. 20). During Bd2133 refinement, we noticed additional density at the sandwich tip, where the trimer interface creates a pocket using Y985, N956 and N1020. Guided by crystal symmetry, we have modelled this as an N-terminal hexapeptide derived from a nearby copy (Fig. 5c).

Negative-stain electron microscopy of full-length Bd2133 (residues 21–1,031) confirmed fibre formation, with dimensions commensurate with an AlphaFold^18^ model (Fig. 5d,h) and our partial structural determination, showing fibre conformational flexibility (Fig. 5d).

### Bd1334 shows chimeric MAT-tip architecture

To address the potential for domain-based diversification, we crystallized the tip of Bd1334 (which shares 75% identity with Bd2133 in the fibre region but then diversifies), from S818 and S914 onwards, diffracting to 2.6 Å and 1.4 Å resolution, respectively (Supplementary Table 2). The structure is composed of three subdomains (Fig. 5e–g): a partial β-helix (D823–A868), a full β-helix (T869–Q1011) and a flat β-rich tip (P1012–W1151). The tip of Bd1334 has no similarity to that of Bd2133, forming a β-sandwich of a completely different, square-like shape. Tip trimers are mediated by edge-to-edge contacts, burying 6,900 Å^2^ per monomer. A composite pocket is formed from two chains, making a tryptophan cage with W1078 and W1102 from one monomer and the terminal W1151 from the opposing monomer (Fig. 5g). W1102 has an unusual *cis* peptide bond often enriched in binding sites^20^. There are no structural homologues for Bd1334 in the PDB, and sequence homologues are limited, with sequence conservation patterns being non-informative. Thus, the Bd1334 data show that MAT tips can be swapped out in evolution (Fig. 5h) and that Bd1334 is probably specific for binding an epitope encountered and bound only by *B. bacteriovorus* bacteria.

### Extension of tip diversification into known adhesive folds

**Fig. 6:**
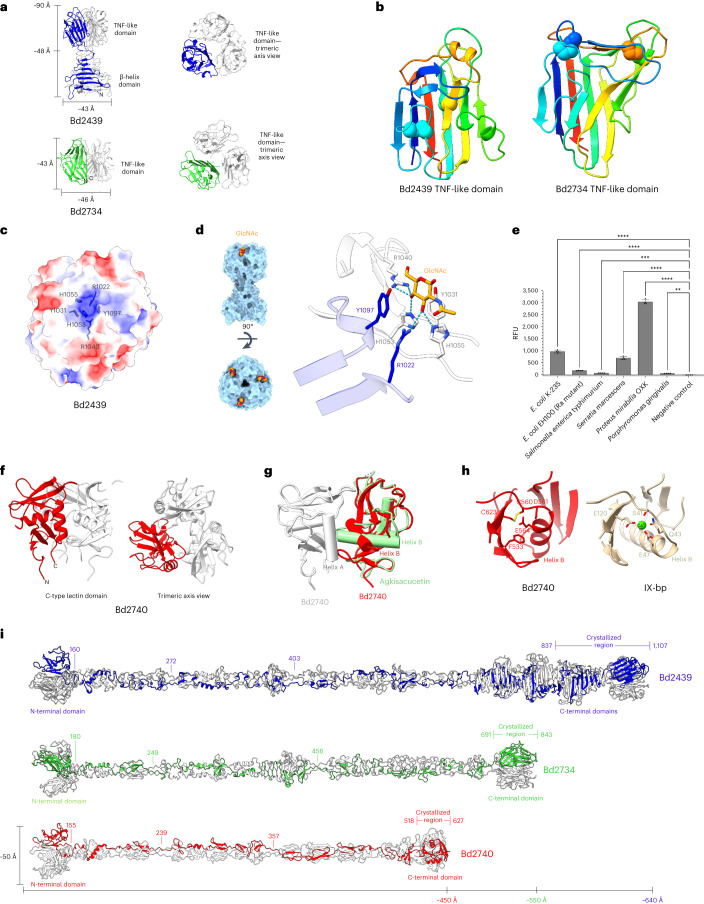
Utilization of known adhesive folds as MAT fibre tips. **a**, The crystal structures of Bd2439_837–1,107_ and Bd2734_691–843_ (hereafter referred to as Bd2439 and Bd2734, respectively) reveal the presence of TNF-like domains at the C-terminus of both fibres. **b**, The two TNF-like domains are shown in the same orientation and coloured as a Jones rainbow to highlight the structural homology of the two domains (disulphides shown as spheres). **c**, The electrostatic potential of the Bd2439 TNF-like domain trimer surface shows a positively charged pocket between two protomers. **d**, GlcNAc-bound Bd2439 (left). Two tyrosines flank the sugar moiety, which orientates its C3 and C4 hydroxyls into the charged pocket to hydrogen bond with two His and two Arg residues (right). **e**, Glycan array screening of Bd2439 against various bacterial LPS (lipopolysaccharide). The bars show the mean of four experiments ± s.d. Only assays in which binding was statistically significant against the negative control are shown (**P ≤ 0.01; ***P ≤ 0.001; ****P ≤ 0.0001 by one-way ANOVA with Dunnett’s test using GraphPad Prism 8.0; *n* = 4 technical replicates). Binding was significant for *E. coli* K235 (*P* < 0.0001), *E. coli* EH100 (Ra mutant), *Salmonella enterica typhimurium* (*P* = 0.006), *Serratia marcescens* (*P* < 0.0001) and *Porphyromonas gingivalis* (*P* = 0.0089), and particularly strong binding was observed to *Proteus mirabilis* LPS (*P* < 0.0001). **f**, The crystal structure of Bd2740_518–627_ (hereafter referred to as Bd2740), which uses a trimeric C-type lectin fold. **g**, Two protomers of Bd2740 are shown (red and white) superposed with the structurally homologous snake venom lectin agkisacucetin (PDB code 3UBU^51^). The fold shows good structural alignment with an RMSD of 2.48 Å over 96 residues, with the major difference being the shift of helix B, which is used by Bd2740 for the trimer interface. **h**, Comparison of Bd2740 (left) with IX-BP (right; the highest-similarity Ca^2+^-binding homologue; PDB code 1BJ3 (ref. ^30^)) shows that Bd2740 does not bind calcium owing to the alteration of helix B and the lack of calcium-binding residues in this region. **i**, Full-length fibres of Bd2439, Bd2734 and Bd2740 modelled using a composite of ColabFold^13^ models and the experimentally determined crystal structures. These models highlight the different lengths of the fibres, and both Bd2734 and Bd2740 lack the β-helical domains of the other fibres. Source data

We next characterized two more divergent NS74s, Bd2439 and Bd2734, obtaining a tip-only variant of Bd2734 (residues T691–L843, 1.8 Å) and a longer construct of Bd2439 (G837–D1107, 1.6 Å; Supplementary Table 2). The resulting structures reveal that both proteins use a related fold (Fig. 6a,b).

Bd2439 is modular, formed by a β-helix N850–V968, a R969 linker that coordinates the carboxy terminus and a compact β-domain tip from S970–D1107 (Fig. 6a). Structure searches with DALI^16^, FoldSeek^21^ and oligomer-sensitive TopSearch^22^ all confirm similarity to trimeric members of the tumour necrosis factor (TNF) family (for example, RMSD 2.3 A for human BAFF, 6FXN^23^), typically receptors and lectins^24^. The Bd2734 tip represents a divergent TNF fold, with large pockets between monomers (Fig. 6a,b). The modified fold of Bd2734 affords it closer similarity to a different TNF subgroup, with the best match to a fucose-specific lectin from *Burkholderia* (2WQ4 (ref. ^25^), 5% sequence identity, 2.3 Å RMSD) and a C1Q-like family protein (4QPY^26^, 15% identity, 2.7 Å RMSD). These two MAT tips are therefore more similar to other TNF proteins than they are to one another (RMSD 3.1 Å). Superimposing with a *Burkholderia* lectin–fucose complex^25^ shows how the Bd2734 pocket can accommodate a terminal monosaccharide (Supplementary Fig. 21). Mapping a sequence alignment (Supplementary Fig. 22) to Bd2439 identifies a conserved interfacial pocket (Y1031, R1040, E1044, H1053 and H1055 from one monomer; R1022 and Y1097 from another; Fig. 6c). These residues coordinate two hydroxyl groups from a bound ethylene glycol (Supplementary Fig. 23). Testing a variety of saccharides, we observed binding of Bd2439 to GlcNAc (from a GlcNAc–MurNAc disaccharide; 1.84 Å resolution dataset), revealing that the above residue cluster recognizes two adjacent ring hydroxyls (Fig. 6d). Thermal unfolding experiments confirm this specificity, revealing a biphasic unfolding whose second phase is shifted in the presence of sugars that contain these features (Supplementary Fig. 24).

We next determined the structure of Bd2740 (residues 517–626, 2.6 Å resolution), whose fold was previously cryptic. Our resulting structure reveals a variant of the C-type lectin fold (Fig. 6f), a widely dispersed superfamily named after its use of calcium to bind sugars, proteins and lipids^27^. The fold has many structural homologues (Fig. 6g,h), the closest being snake venom agkisacucetin (PDB code 1v4l^28^, 2.1 Å RMSD). TopSearch^22^ analysis indicates that the majority of C-type lectins use a different face for trimerization, and the only homologue to match Bd2740 is the major viral adhesion protein Mtd from *Bordetella* bacteriophage (PDB code 2iou^29^), which binds host pertactin on its upper surface. Bd2740, like agkisacucetin and Mtd, has dispensed with the Ca-binding motif (Fig. 6h; with the closest Ca-binding homologue IX-BP, PDB code 1BJ3 (ref. ^30^), 2.53 Å RMSD). The largest pockets and clefts are upwardly facing (distal to the fibre base) akin to Mtd. These observations reveal further potential for utilization of known adhesive folds at MAT fibre termini and show that these can diverge over time into distinct variants (Fig. 6i).

### Screening of adhesin specificity for common glycans

No previous identification linked any *B. bacteriovorus* protein and adhesin to specific prey; we therefore assayed 6 MAT constructs (fluorescently labelled tips of Bd3182, Bd2133, Bd1334, Bd2439 and Bd2734 plus full-length Bd2133_21–1,031_) against a commercial O-antigen array (26 glycans from 11 bacteria). Bd2439 showed strong binding to *Proteus mirabilis* OXK glycan, with weaker (but significant) binding to glycans from *E. coli* K-235 and *Serratia marcescens* (Fig. 6e and Supplementary Fig. 25). Our demonstration of a MAT binding pocket capable of recognizing two adjacent hydroxyls presumably explains part of this specificity, but the ubiquity of this feature in surface glycans and relative specificity of Bd2439 suggest that multiple flanking pockets will provide additional selectivity. This study has positively identified prey surface recognition proteins in predatory bacteria.

## Discussion

Using independent but complementary approaches, we identified and characterized a MAT superfamily of trimeric fibre proteins with diverse adhesive tips, able to fulfil the long-sought-after role of multiplicity of molecular recognition and handling of diverse epitopes (across a wide prey range), by the bacterial predator *B. bacteriovorus* (Supplementary Fig. 26). The MAT family proteins fulfil the logical expectations of a surface recognition system, which are as follows: (1) surface exposed on the predator and of sufficient length to project and contact prey, (2) present in numbers that scale to prey diversity, (3) mosaic in nature in which one ‘end’ is conserved for presentation and retention on the predator and the other is diversified for different prey, (4) relatively stable and robust for the extracellular environment and (5) able to undergo dynamic re-localization associated with prey interactions during invasive predation.

We postulate that MAT proteins are dynamically expressed on the predator surface as they interact with and enter prey—in some cases at the predator’s invasive pole and in other cases at the flagellar pole (Bd1334) or midcell, even with one or two foci per family member.

MAT adhesins may fulfil several roles during prey entry, from initial attachment through dynamic relocation to porthole resealing and positional stabilization of the predatory-associated vesicle inside prey. Multiple, dynamic locations for MAT members are in keeping with the various staged processes of the *Bdellovibrio* life cycle that require ever-changing (but distinct) coordination between the surfaces of predator and prey (Supplementary Fig. 26).

One adhesin could recognize several prey-surface types (shared chemistry), and one prey could be recognized by several adhesins, hence the MAT family’s large redundancy and diversity to cope with a wide prey range. Thus, it is expected that the deletion of individual MAT fibre genes rarely yielded mutants significantly defective in predation. However, a slight reduction in prey entry efficiency was seen for deletion mutants of Bd2734 and Bd2740, which are structurally divergent but have similar expression profiles and are encoded at nearby genetic loci^31,32^. Bd2734 and Bd2740 are two of the shorter MAT fibres (843 and 627 aa, respectively (Fig. 6i)), which are relevant given that one of the short MATs we were unable to crystallize (Bd2872, 626 aa) was previously identified in a screen for predation-deficient mutants^33^.

As the flagellum and sheath are re-absorbed back inside the periplasm of *B. bacteriovorus* upon invasion^6^, the predatory vesicle is probably bounded by a predator-derived membrane, and reorganization at the *Bdellovibrio* flagellar pole is likely to be associated with the laying down of our identified vesicle (Supplementary Fig. 26). Resealing the entry port could involve PBP1b–CpoB_Bd0635_-mediated events and would be accompanied by a septation-like membrane bifurcation event, pinching off the vesicle.

We were able to structurally characterize six separate MAT family members for *B. bacteriovorus* HD100, revealing that these typically large (~1,000 aa) proteins can terminate in an S74 motif characteristic of viral tailspikes^14^ (Bd3182), previously uncharacterized β-sandwiches (Bd2133 and Bd1334) and domains of known adhesive usage (the TNF-like domains of Bd2439 and Bd2734 and the C-type lectin fold of Bd2740). Other bacterial strains, predatory and otherwise, may use further domains still (a selection is provided in Supplementary Tables 3 and 4).

From our experimentally derived structural data on six independent MATs, plus usage of AlphaFold^18^ models, meandering trimerization appears to be an invariant property of the fibres, although none of the NS74 domains show strand swapping; that is, these capping regions fold independently; this independence may aid ‘tip swapping’ as predators diversify the MAT repertoire. The (predicted) N-terminal domain of the *B. bacteriovorus* MAT proteins is invariable in structure and would most probably sit proximal to the predator cell surface to productively position the variable C-terminal domains. The N-terminal domain has no structural homologues in the PDB but would presumably tightly bind a *B. bacteriovorus* surface element (whose identification awaits further study; potential interplay between adhesion and type IV pili^34,35^ may also be of interest). In light of our demonstration of surface localization, it is informative that the dimensions from our experimental Bd2133 electron micrographs and AlphaFold^18^ models are compatible with surface fibre-like features (some attached to prey) identified on tomograms^6^. Likewise, the features of the vesicle partly match those of a ‘bubble-like’ structure identified at the entry portal^6^.

We were able to confidently identify a binding partner for MAT protein Bd2439, which showed strong binding to glycans of the known prey species *Proteus mirabilis*^36^. The fact that none of the single-gene-deletion mutants were significantly defective in predation on *Proteus mirabilis* hints that there are multiple factors and several MAT proteins involved, but our GlcNAc-bound Bd2439 structure identifies a potential motif that may be used (in part) to select for saccharides in *Proteus mirabilis* and *Proteus mirabilis*-like glycans.

The multiplicity of the MAT family prevents exhaustive and absolute specificity determination (as suggested by our knockout phenotypes), but we use Bd2439 as a proof of principle to link to recognition of bacteria with *Proteus*-like O-antigen chemistries, revealing a molecular basis for *B. bacteriovorus* prey handling. Our observations provide a basis for future characterization and manipulation of the bacterial predator–prey relationship.

## Methods

### Bacterial strains and culture

*B. bacteriovorus* HD100, and fluorescently tagged or gene-deletion strains, were grown predatorily on stationary-phase *E. coli* S17-1 prey (16 h, 29 °C), in Ca–HEPES buffer (5.94 g l^−1^ HEPES free acid, 0.284 g l^−1^ calcium chloride dihydrate, pH 7.6), or on YPSC (Yeast Extract Peptone Sodium Acetate Calcium) overlay platesas plaques within a lawn of *E. coli* S17-1 prey, as described previously^37^. To provide selection for fluorescent-tag or gene-deletion constructs, 50 mg ml^−1^ of kanamycin sulphate was added to growth media where appropriate. *E. coli* S17-1 cells, for *B. bacteriovorus* prey or fluorescent-tagging and gene-deletion manipulations, were grown for 16 h in YT broth (5 g NaCl, 5 g Difco Yeast extract, 8 g Difco Bacto Tryptone per litre, pH adjusted to 7.5 with NaOH) at 37 °C with shaking at 200 rpm.

### Plasmid and strain construction

The primers used to generate fluorescently tagged or gene-deletion strains of *B. bacteriovorus* are documented in Supplementary Table 5, and the plasmid constructs used are documented in Supplementary Table 6. PCR amplification was performed with Phusion polymerase (New England Biolabs) according to the manufacturer’s instructions, using primers listed in Supplementary Table 5.

### Generation of markerless deletion mutants

To construct markerless gene deletions of *bd1334*, *bd2133*, *bd2439*, *bd2734*, *bd2740* and *bd3182*, between 750 bp and 1,000 bp of DNA upstream and downstream of the gene of interest were cloned into the suicide vector pK18*mobsacB* by Gibson assembly^38^ using the NEBuilder HiFi DNA assembly cloning kit (New England BioLabs). Gene-deletion vectors were introduced into *B. bacteriovorus* by conjugation (using donor *E. coli* S17-1 strains) and subsequently cured of the donor plasmid by sucrose suicide counter-selection, resulting in the integration of the gene knockout constructs via double-crossover homologous recombination. This process is further detailed and was described previously^9,39^. All gene deletions were verified by Sanger sequencing. To visualize any effect of the single-gene deletions *bd2734* and *bd2740*, during the initial stages of the predatory cycle, a C-terminal mCherry fusion to the constitutively expressed PilZ protein Bd0064 was introduced via single-crossover recombination, illuminating the *B. bacteriovorus* cell body, as described previously^40–42^.

### Generation of Bd3182 mutation of catalytic serine S782 to alanine

The S782A mutation of Bd3182 was introduced by amplifying the gene on either side of the S74 codon and introducing the mutation in the primers (Supplementary Table 4), followed by Gibson assembly as above.

### Fluorescence tagging of *B. bacteriovorus* gene products

Protein fluorophore tags (mCherry, mNeonGreen, mTFP or mCerulean3) were fused to the C-terminus of target genes by PCR amplification of the target gene, without its stop codon, and amplification of the fluorophore gene, followed by Gibson cloning using the NEBuilder HiFi or GeneArt assembly kits (New England Biolabs) according to the manufacturer’s instructions, into the mobilizable broad-host-range vector pK18mobsacB^43^. Each construct was conjugated into *B. bacteriovorus* HD100 as described previously^37^. In some cases, a particular fluorescent tag, such as mCherry for Bd2133, was not tolerated by a gene under study for reasons unknown. In such cases, a different colour combination of strains was used in experiments. Single-crossover constructs of Bd0635–mCherry and Bd0635–mTeal were obtained by standard cloning methods rather than by Gibson assembly (details in Supplementary Table 4).

For multiple fluorophore combinations, either the Bd0064 or the CpoB_Bd0635_ tagging constructs were made with 1,000 bp of flanking DNA and ex-conjugants of these were subjected to sucrose suicide selection to generate a double-crossover event, replacing the genomic copy of the gene with the tagged version, via the same methodology used to generate single-gene-deletion strains (above). The second fluorophore-tagged gene was then introduced, where required, as above by single crossover into the *B. bacteriovorus* genome, as before.

### Labelling of cell wall muropeptides with fluorescent d-amino acids and imaging

Pulse labelling of predator-modified prey peptidoglycan during early predation events with the ‘blue’ fluorescent d-amino acid HADA to label predatory porthole formation was carried out as described previously^1^. *B. bacteriovorus* HD100 cells were grown predatorily for 16 h at 29 °C on stationary-phase *E. coli* S17-1 prey, until the prey culture was lysed. The *B. bacteriovorus* were then filtered through a 0.45 µm filter (yielding ~2 × 10^8^ plaque-forming units (pfu) ml^−1^) and concentrated 30 times by centrifugation at 12,000 × *g* for 5 min. The resulting pellet was resuspended in Ca–HEPES buffer (2 mM CaCl_2_, 25 mM HEPES, pH 7.6). *E. coli* S17-1 cells were grown for 16 h in Luria–Bertani medium at 37°C with shaking at 200 rpm and were back diluted to an optical density at 600 nm (OD_600_) of 1.0 in Ca–HEPES buffer (yielding ~1 × 10^9^ colony-forming units (cfu) ml^−1^). A total of 50 μl of this *B. bacteriovorus* culture was mixed with 40 µl of the pre-labelled *E. coli* and 30 µl of Ca–HEPES buffer and incubated at 30 °C. For pulse labelling, 1.2 µl of a 50 mM stock of HADA in DMSO was added 10 min before the sampling time point for microscopy and returned to 30 °C incubation. At each time point, all of the 120 µl predator–prey sample was transferred to 175 µl ice-cold ethanol and incubated at −20 °C for at least 15 min to fix the cells. The cells were pelleted by centrifugation at 17,000 × *g* for 5 min, washed with 500 µl PBS and resuspended in 5 µl Slowfade (Molecular Probes), and stored at −20 °C before imaging. Samples (2 µl) were imaged using a Nikon Ti-E inverted fluorescence microscope equipped with a Plan Apo 100× 1.45 Ph3 objective, a DAPI filter cube and an Andor Neo sCMOS camera using DAPI settings for detection of HADA (emission maximum 450 nm).

### *B. bacteriovorus* predation on *E. coli* in liquid culture

Assays were based on, and modified from, those detailed in a previous study^11^. In summary, *B. bacteriovorus* gene-deletion mutants were grown predatorily (as above). The pfu inputs for each gene-deletion strain were matched using the SYBR Green DNA stain, to ensure that equal titres of *B. bacteriovorus* for each strain were used as starting inputs. Briefly, *B. bacteriovorus* were incubated with SYBR Green dye for 90 min (300 rpm double orbital, in darkness, in triplicate), before fluorescence was measured using a FLUOstar Omega plate reader (BMG Labtech; excitation 485 nm, emission 520 nm, gain 800), with fluorescence values being interpolated into relative pfu per ml counts using a pfu–SYBR Green fluorescence correlation curve. *E. coli* S17-1 cells were grown as above and subsequently back diluted to OD_600_ 1.0 (approximately 1 × 10^9^ cfu ml^−1^) in dilute nutrient broth. Predatory cultures (containing approximately 1 × 10^9^ cfu ml^−1^ *E. coli* prey and 1 × 10^8^ pfu ml^−1^ *B. bacteriovorus* WT or mutant) were inoculated in triplicate into a black OptiPlate (Corning), along with media-only, *B. bacteriovorus*-only (no prey) and prey-only (no *B. bacteriovorus*) controls. The OD_600_ of the predatory culture was measured every 20 min, for 18 h (200 rpm, double orbital, in triplicate) to give a prey survival curve, in which a drop in OD_600_ is indicative of successful predation and prey lysis, because prey cells, but not predators, are large enough to produce an optical density at 600 nm.

OD_600_ data were exported to Excel 2016 and then analysed using CurveR, according to the method documented previously^11^ to analyse prey cell lysis and predation dynamics. *R*_max_ indicates the maximum rate of prey cell lysis. *S* indicates the inflection point, the time at which the maximum rate of prey cell lysis (*R*_max_) occurs, that is, the steepest point on the curve.

To further assess the effect of deletion in early stages of the predatory cycle, synchronous predatory infections of *B. bacteriovorus* HD100:Bd0064mCherry:Δ*bd2734* and HD100: Bd0064mCherry:Δ*bd2740* on *E. coli* S17-1 PZMR100 were set up as described above, with aliquots removed at 30 min after the mixing of predator and prey. Cells were immobilized on a thin 1% Ca–HEPES buffer agarose pad and imaged as above. Images were minimally processed using the sharpen and smooth tools (ImageJ), with adjustments to brightness and contrast to ensure clarity. Full fields of view were visually scored for complete *B. bacteriovorus* entry as described previously^44^. The percentage of bdelloplasts with a fully entered *B. bacteriovorus* at 30 min was derived from manual inspection of the total number of bdelloplasts with *B. bacteriovorus* fully entered at 30 min divided by the total number of bdelloplasts visually characterized. Images were analysed from three biological repeats. Each deletion mutant strain was imaged and analysed alongside *B. bacteriovorus* HD100 (control) within the same experiment.

### Phase-contrast and epifluorescence microscopy

The in vivo fluorescence of MAT protein–XFP tags (XFP meaning any of the different fluorescent protein tags) during the predatory life cycle was examined. Approximately synchronous predation of *E. coli* S17-1 PZMR100 by *B. bacteriovorus* strains was prepared by combining a 10-times-concentrated *B. bacteriovorus* predatory culture with *E. coli* S17-1 PZMR100 (standardized to an OD_600_ of 1) and Ca–HEPES at a ratio of 5:4:3, respectively, as described previously^32,45^. Progress through the predatory life cycle (and position of the fluorescently tagged protein under investigation) was visualized via fluorescence microscopy (10 s exposure) at the following time points (in minutes): 0, 15, 30, 45, 60, 120, 180 and 240, by withdrawing 10 μl of the culture and immobilizing on a thin 1% Ca–HEPES buffer agarose pad. Cells were visualized with a Nikon Ti-E inverted epifluorescence microscope equipped with a Plan Apo 100× Ph3 oil objective lens (Numerical Aperture 1.45) and the following filters: mCherry (excitation 550–600 nm, emission 610–665 nm), GFP, green fluorescent protein, (for mNeonGreen; excitation 460–500 nm, emission 515–530 nm), CFP, cerulean fluorescent protein, (mCerulean; excitation 420–450 nm, emission 470–490 nm) and TFP, teal fluorescent protein, (mTeal; excitation 420–450 nm, emission 515–530 nm).

Images were acquired using an Andor Neo sCMOS camera with Nikon NIS software and analysed using ImageJ (Fiji). Images were minimally processed using the sharpen and smooth tools, with adjustments to brightness and contrast. Where stated, false colour was used in channels for display purposes.

### Image analysis

Images were manipulated with ImageJ (Fiji distribution) software using the sharpen and smooth tools, and by duplication of the region of interest for presentation. Images were analysed using the MicrobeJ plug-in for ImageJ^46^, which automates the detection of bacteria within an image. Attack-phase *B. bacteriovorus* cells were detected with parameters as default, with fluorescence detected by the foci method with default parameters and associated with parent bacteria with a tolerance of 0.1 µm.

For the analysis of CpoB–mCherry foci with Bd0064–mCerulean cytoplasmically labelled *B. bacteriovorus*, prey *E. coli* were detected as bacteria, *B. bacteriovorus* cells were detected using the medial axis method in the mCerulean channel and associated with bacteria with a tolerance of 0.1 µm, and CpoB–mCherry foci were detected using the fit shape as circle method in the mCherry channels with a maximum area of 0.25 µm^2^ and associated with *B. bacteriovorus* cells with a tolerance of 1 µm.

Manual inspection of the analysed images confirmed that the vast majority of cells and foci were correctly assigned. The shape measurements including the angularity, area, aspect ratio, circularity, curvature, length, roundness, sinuosity, solidity and width were measured for each type of cell.

### Co-localization analysis

Images were analysed using ImageJ software (Fiji), using the cell counter plug-in. Adjustments to brightness and contrast for whole images were made until CpoB_Bd0635_ (mCherry: Bd2133–mNeonGreen or mTeal (Bd2439–mCherry or Bd2740–mCherry)) and Bd2133–mNeonGreen, Bd2439–mCherry or Bd2740–mCherry foci, if present, were visible. Bdelloplasts were then manually (visually) scored for the presence of a CpoB_Bd0635_ focus. Of the bdelloplasts that contained a CpoB_Bd0635_ focus, the number of bdelloplasts for which a Bd2133, Bd2439 or Bd2740 focus was coincident was scored for approximately 60 bdelloplasts, originating from two biological replicates.

### Visualization of external mCherry-tagged MAT protein expression

Semi-synchronous predation experiments were set up as above. Samples of 1.4 ml were pre-fixed in 0.25% paraformaldehyde and recovered by centrifugation at 17,000 × *g* for 2 min, then fixed in 2.5% paraformaldehyde in Dulbecco’s PBS for 10 min at 37 °C. Cells were recovered by centrifugation at 17,000 × *g* for 2 min, washed twice in 100 µl blocking solution (2% bovine serum albumin, BSA, in Dulbecco’s phosphate buffered saline, PBS) and then incubated in blocking solution for 45 min. Samples were further incubated in anti-mCherry antibody (Invitrogen PA5-34974) diluted 1:1,000 in blocking solution for 1 h. Cells were recovered by centrifugation at 17,000 × *g* for 2 min, then washed twice in blocking solution before a final incubation with secondary antibody of goat anti-rabbit IgG with Alexa Fluor plus 488 (Invitrogen A32731). Cells were then washed twice with blocking solution before imaging with a Nikon Ti-E microscope as described above. Some samples were further stained with FM46-4 by resuspension in 10 mM stain in water. Images were analysed using ImageJ software (Fiji). Adjustments to brightness and contrast for whole images were made until FM46-4-stained flagella and anti-mCherry foci were visible. Cells were then manually (visually) scored for the presence of flagella (FM46-4 red channel) and the presence and positioning of anti-mCherry foci (GFP channel).

### Microscopic statistical analysis

Statistical analysis was performed in Prism 8.2.0 (GraphPad). Data were first tested for normality and then analysed using the appropriate statistical test. The number of biological repeats, *n* values for cell numbers and the statistical test applied are described within each figure legend.

### Cloning, expression and purification

Bd2133_21–1,031_ was synthesized and inserted into pET29a between NdeI and XhoI (Twist Bioscience), producing a construct with an N-terminal PelB leader sequence and a hexa-His tag. Bd2133_662–1,031_ and Bd3182_668–922_ were cloned into pCold1 using NdeI and XhoI restriction sites. The final constructs contained an N-terminal 24-amino-acid tag containing a transcription-enhancing element, a hexa-His tag, a factor Xa cleavage site and a Tobacco Etch Virus (TEV) cleavage site. Bd2133_910–1,031_ was produced by Q5 (New England Biolabs) deletion using the Bd2133_662–1,031_ plasmid as a template. Bd3182_668–922_S782A_ was produced by Q5 mutagenesis using Bd3182_632–922_ as a template. Bd1334_818–1,031_ and Bd1334_914–1,151_ were cloned into pCold1 using XhoI and HindIII restriction sites. The final constructs contained an N-terminal 30-amino-acid tag containing a transcription-enhancing element, a hexa-His tag, a factor Xa cleavage site and a TEV cleavage site. Bd2734_691–843_ was cloned into pET26b using NcoI and XhoI restriction sites. The final construct contained a pelB leader sequence, Met-Ala and a hexa-His tag. Bd2439_837–1,107_ was cloned into pET26b using BamHI and XhoI restriction sites. The final construct contained a pelB leader sequence, Met-Asp-Ile-Phe-Ile-Asn-Ser-Asp-Pro and a hexa-His tag. Bd2740_518–627_ was synthesized and inserted into pET29a between NdeI and XhoI (Twist Bioscience), producing a construct with an N-terminal PelB leader sequence, a hexa-His tag and an Ala-Ser linker.

Constructs were expressed in *E. coli* BL21 RIPL (DE3). Cells were grown to an OD_600_ of 0.5–0.7 and induced with 0.5 mM IPTG (isopropyl ß-D-1-thiogalactopyranoside) at 18 °C for 16 h. Cells were harvested by centrifugation and resuspended in 50 mM HEPES, 500 mM NaCl and 20 mM imidazole, pH 7.5. The cells were lysed by sonication on ice with the exception of Bd2133_21–1,031_, which was lysed by three passages through an EmulsiFlex C3 high-pressure homogenizer (Avestin). The lysates were loaded onto a 5 ml Ni-NTA column (GE Healthcare) and washed with 10 column volumes of lysis buffer. The protein was eluted with a gradient of 20–500 mM imidazole. TEV was added to constructs containing cleavage sites that were subsequently dialyzed into 150 mM NaCl, 20 mM HEPES and 20 mM imidazole, pH 8.0, overnight at 4 °C. The protein was then passed through a 1 ml Ni-NTA column to remove TEV and uncleaved protein. The proteins were finally passed through a Superdex 200 26/60 column (GE Healthcare), which was pre-equilibrated with 20 mM HEPES and 150 mM NaCl, pH 7.5.

To express selenomethionine-labelled Bd2133_662–922_, a 60 ml overnight culture of BL21 RIPL (DE3) cells in LB was centrifuged and resuspended in minimal media supplemented with kanamycin and chloramphenicol to a final volume of 1 l. At OD_600_ 0.4, 0.1 g of lysine, 0.1 g of threonine, 0.1 g of phenylalanine, 0.05 g leucine, 0.05 g of isoleucine, 0.05 g of valine and 0.06 g of selenomethionine were added to the culture. After 15 min, IPTG was added to 1 mM. Cells were harvested by centrifugation after 16 h at 18 °C and stored at −20 °C. SDS-PAGE (sodium dodecyl sulfate polyacrylamide gel electrophoresis) gels were imaged using Quantity One v4.6.8.

### Crystallization and data collection

For crystallization, proteins were concentrated to 3–10 mg ml^−1^ using a spin concentrator. Protein was mixed with precipitant in a 1:1 ratio, with drop sizes of 0.8–4 μl, and crystallized using vapour diffusion at 18 °C.

Crystals of Bd3182_632–922_ formed in Morpheus screen C1 (0.03 M sodium nitrate, 0.03 M sodium phosphate dibasic, 0.03 M ammonium sulphate, 0.1 M imidazole and MES (2-ethanesulfonic acid), pH 6.5, 20% v/v PEG (polyethylene glycol) 500 MME (monomethylether), 10% w/v PEG 20000) in spacegroup P2_1_, Morpheus screen G12 (1.0 M sodium citrate tribasic dihydrate, 0.1 M HEPES, pH 7.0) with spacegroup I2 and Proplex screen E3 (0.1 M magnesium acetate tetrahydrate, 0.1 M MES, pH 6.5, 10% w/v PEG 10000) in a different P2_1_ spacegroup. Crystals were subject to diffraction at Diamond Light Source at 100 K at wavelength 0.970–0.976 Å on beamline IO3. The phases were solved using Phenix MR^47^ with a low sequence homology (<10% sequence ID) ensemble of pruned T5 phage-tail fibre (PDB code 4UW8 (ref. ^14^)) and GP12 (PDB code 3GW6 (ref. ^10^)).

Crystals of Bd2734_691–843_ grew in JCSG+ screen A3 (0.2 M ammonium citrate dibasic, 20% w/v PEG 3350) in spacegroup P2_1_. Crystals were subject to diffraction at Diamond Light Source at 100 K at wavelength 0.98 Å on beamline IO4. The phases were solved using Phenix MR^47^ with a homology model generated by ColabFold^13^.

Crystals of Bd1334_914–1,151_ formed in PACT screen B9 (0.2 M lithium chloride, 0.1 M MES, pH 6.0, 20% w/v PEG 6000) in spacegroup R3. Crystals were subject to diffraction at Diamond Light Source at 100 K at wavelength 0.98 Å on beamline IO4. Crystals of Bd1334_818–1,151_ grew in Morpheus screen C1 (0.03 M sodium nitrate, 0.03 M sodium phosphate dibasic, 0.03 M ammonium sulphate, 0.1 M imidazole and MES, pH 6.5, 20% v/v PEG 500 MME, 10% w/v PEG 20000) in spacegroup C2. Crystals were subject to diffraction at Diamond Light Source at 100 K at wavelength 1 Å on beamline IO4. The phases of the Bd1334_818–1,151_ crystals were solved using Phenix MR^47^ with a homology model generated by ColabFold^13^. The phases of the Bd1334_914–1,151_ crystals were solved using Phenix MR^47^ with a fragment of the Bd1334_818–1,151_ structure.

Crystals of Bd2439_837–1,107_ grew in multiple conditions: PACT H3 (0.2 M sodium iodide, 0.1 M Bis–Tris propane, pH 8.5, 20% w/v PEG 3350) in spacegroup P2_1_22_1_ and MIDASplus E9 (0.1 M lithium sulphate, 0.1 M tris, pH 8.0, 25% v/v Jeffamine ED-2003) in spacegroup P4. Crystals were cryocooled in the mother liquor with an addition of 25% ethylene glycol. Crystals were subject to diffraction at the European Synchrotron Radiation Facility at 100 K at wavelength 0.976 Å on beamlines id30a and id30b. The phases were solved using Phenix MR^47^ with a homology model generated by ColabFold^13^. Bd2439_837–1,107_–GlcNAc–MurNAc crystals were grown in PACT H3 as above, supplemented with 100 mM GlcNAc–MurNAc. To prevent ethylene glycol entering the binding pocket, these crystals were cryoprotected with 25% PEG 400.

Crystals of native and selenomethionine-labelled Bd2133_662–1,031_ grew in Morpheus screen B3 (0.03 M sodium fluoride, 0.03 M sodium bromide, 0.03 M sodium iodide, 0.1 M imidazole and MES, pH 6.5, 20% v/v glycerol, 10% w/v PEG 4000) in spacegroup P6_3_22. Crystals were subject to diffraction at Diamond Light Source at 100 K at wavelength 0.98 Å (native) on beamline IO4-1 and 0.89 Å (selenomethionine protein) on beamline IO3. The phases of the Bd2133_662–1,031_ crystals were solved using experimental phasing. Selenium sites of the selenomethione-labelled protein crystals were identified via SAD by SHELX^48^, and Phenix^47^ Autosol was used to phase the data via SIRAS using the selenomethionine and native datasets. For model building, the symmetry was dropped to p63 to allow asymmetric modelling of the N-terminal beta strands. Molecular replacement of the selenomethioine-labelled protein structure was used to solve the phases of the native dataset. Crystals of Bd2133_910–1,031_ grew in Morpheus screen A4 (0.03 M magnesium chloride, 0.03 M calcium chloride, 0.1 M imidazole and MES, pH 6.5, 12.5% v/v MPD (2-methyl-2,4-pentanediol), 12.5% PEG 1000, 12.5% w/v PEG 3350) in spacegroup P2_1_2_1_2_1_. Crystals were subject to diffraction at Diamond Light Source at 100 K at wavelength 0.75 Å on beamline IO4 and solved using a fragment of the larger model.

Bd2740_518–627_ crystallized in MIDAS screen F1 (0.1 M HEPES, pH 6.5, 40% polypropylene glycol bisaminopropylether 2000) in space group P2_1_2_1_2_1_. Crystals were subject to diffraction at Diamond Light Source at 100 K at wavelength 0.979 Å on beamline IO4 and solved using molecular replacement with a ColabFold^13^ model.

All structures were manually completed and altered in CCP4i2 v1.1.0 and COOT 0.9.8.1 (ref. ^49^), and refined using Phenix 1.20.1-4487 (ref. ^47^). Statistics for data collection and refinement are presented in Supplementary Table 2.

### Glycan arrays

Glycan arrays were performed as a service by Z Biotech. The array was blocked for 30 min using glycan array blocking buffer (Z Biotech item number 10106). Alexafluor-555-labelled samples were diluted in glycan array assay buffer (Z Biotech item number 10107) to the desired concentrations and then applied directly to the array. The array was covered with an adhesive film and shaken at 80 rpm for 1 h at room temperature. The array was then washed three times with glycan array assay buffer and two times with MilliQ water. The array was read using an Innopsys InnoScan 710 Microarray Scanner with a high-power laser at 1× photomultiplier gain. Proprietary software was used to detect each spot on the array and calculate the relative fluorescence unit (RFU) intensity for each spot. Background RFU was subtracted from each spot’s RFU value. The median of four repeat spots was determined for each glycan.

### Electron microscopy

To visualize a full-length fibre, Bd2133_21–1,031_ was subjected to negative-stain electron microscopy. Formvar and carbon grids (EMResolutions) were glow discharged with an Elmo glow discharge system (Cordouan) for 1 min at 3 mA. The protein was diluted to 1 μg ml^−1^ in 150 mM NaCl and 20 mM HEPES, pH 7.5, and 5 μl of protein was added followed by 5 μl of 2% uranyl acetate. Micrographs were taken using a 200 kV LaB6 Jeol 2100Plus 200 kV with a Gatan OneView IS at 50,000× magnification.

### Reporting summary

Further information on research design is available in the Nature Portfolio Reporting Summary linked to this article.

### Supplementary information

Supplementary Information Supplementary Notes 1 and 2, Figs. 1–26 and Tables 1–7.

Reporting Summary

### Source data

Source Data Fig. 4 Unprocessed gel for Fig. 4a.

Source Data Fig. 6 Source data for Fig. 6e (glycan array).

## Data Availability

Atomic coordinates have been deposited in the PDB with accession codes 8ONC (10.2210/pdb8ONC/pdb; Bd3182_632–922_ form 1), 8OJN (10.2210/pdb8OJN/pdb; Bd3182_632–922_ form 2), 8ONB (10.2210/pdb8ONB/pdb; Bd3182_632–922_ form 3), 8OND (10.2210/pdb8OND/pdb; Bd2133_662–1,031_), 8OK3 (10.2210/pdb8OK3/pdb; Bd2133_910–1,031_), 8OML (10.2210/pdb8OML/pdb; Bd1334_818–1,151_), 8ON4 (10.2210/pdb8ON4/pdb; Bd1334_914–1151_), 8OL4 (10.2210/pdb8OL4/pdb; Bd2439_837–1,107_ with GlcNAc–MurNAc), 8ONF (10.2210/pdb8ONF/pdb; Bd2439_837–1,107_ with ethylene glycol), 8OKW (10.2210/pdb8OKW/pdb; Bd2734_691–843_) and 8OKS (10.2210/pdb8OKS/pdb; Bd2740_518–627_). The mass spectrometry proteomics data have been deposited at the Oxford Research Archive (ORA) repository (https://ora.ox.ac.uk/objects/uuid:e8eff929-e8d9-4a8c-baf5-eccaf5cd7926). Molecular replacement models include the PDB 4UW8 T5 tail fibre (10.2210/pdb4UW8/pdb) and the PDB 3GW6 intramolecular chaperone (10.2210/pdb3GW6/pdb). Source data are provided with this paper.
