# Supplementary material for: Bdellovibrio bacteriovorus uses chimeric fibre proteins to recognize and invade a broad range of bacterial hosts
Source: Nat Microbiol. 2024 Jan 4;9(1):214–27. doi: 10.1038/s41564-023-01552-2 (PMC10769870; doi:10.1038/s41564-023-01552-2)
Supplement: Supplementary file 1 — Supplementary Notes 1 and 2, Figs. 1–26 and Tables 1–7. [file 41564_2023_1552_MOESM1_ESM.pdf]

nature microbiology

Article https://doi.org/10.1038/s41564-023-01552-2

Bdellovibrio bacteriovorus uses chimeric fibre proteins to recognize and invade a broad range of bacterial hosts

In the format provided by the authors and unedited

Supplementary Material

Supplementary Note 1- Enrichment of predatory vesicles and proteomics

As $CpoB_{Bd0635}$-mCherry foci associated with debris were small (<0.2 µm), we enriched 24 h predatory cultures in which virtually all prey cells had been lysed, by predators, with different experimental approaches involving differential centrifugation but yielding similar vesicle enrichments as in Supplementary Figure 8.

Enrichment 1- Percoll gradient

Two 1 litre predatory cultures of *B. bacteriovorus* $CpoB_{Bd0635}$-mCherry were grown for 24 hours at 29˚C with 200 rpm shaking (1 L Ca/HEPES buffer + 60 ml *E. coli* S17-1 grown in YT broth for 24 h, at 37˚C with shaking at 200 rpm + 50 ml of predatory *B. bacteriovorus* HD100 CpoB-mCherry culture in Ca/HEPES). The majority of cells were pelleted by centrifugation at 5,000 x *g* for 30 minutes. The remaining cells, cell debris and vesicles were then pelleted at 15,000 x *g* for 30 min. This pellet was resuspended in Percoll separation solution (24.7 ml Percoll + 5 ml 3 M NaCl + 20.3 ml H_2O) and centrifuged at 17,000 x *g* for 30 min to separate the vesicles and prey debris from the remaining cells. A band of debris formed near the top of the Percoll gradient, and this was resuspended in 20 ml Percoll separation solution and the gradient spun again. This process was repeated one more time, and then the purified band was mixed 1:4 with H_2O and spun at 5,000 x *g* to remove the Percoll. The resulting pellet was resuspended in 100 µl TE.

Enrichment 2; preps 2 and 3- Filtration and ultracentrifugation

One litre of a predatory culture of *B. bacteriovorus* CpoB-mCherry was grown for 24 hours at 29˚C with 200 rpm shaking (1 L Ca/HEPES buffer + 60 ml *E. coli* S17-1 or *P. putida* grown in YT broth for 24 h, at 37˚C with shaking at 200 rpm + 50 ml of predatory *B. bacteriovorus* HD100 CpoB-mCherry culture in Ca/HEPES). The majority of "contaminating" whole cells were pelleted by centrifugation at 5,000 x *g* for 30 minutes. The remaining cells were removed by filtering through 0.2 µm filters (Sartorius; one filter per 50 ml supernatant). Eight samples of 20 ml of this filtrate were centrifuged at 160,000 x *g* for 30 minutes. The supernatant was removed, and a further 20 ml filtrate was added to each tube, repeating twice further (a total of 80 ml filtrate was therefore pelleted into each of 8 tubes). All 8 pellets were pooled and resuspended in a final volume of 100 µl TE for *E. coli* or 400 µl TE for *P. putida*.

Proteomic analysis by LC/MS/MS

Proteomic analyses of the vesicle samples prepared above were carried out in the Chemistry Research Laboratory Department in the Mass Spectrometry Facility, working on a subcontract from the Oxford University Advanced Proteomics Unit Oxford UK.

50 µl samples of the final vesicle samples prepared in the enrichment processes above were digested by trypsin and lysC according to the Filter-Aided Sample Preparation (FASP) method (Wisniewski et al., 2009), a protocol using Microcon 30k centrifugal ultrafiltration units operated at 10,000 g.

In the ultrafiltration unit, 50 μg of total protein was mixed with 200 μL of 8 M urea in 0.1 M Tris/HCl, pH 8.5 (buffer 1), then centrifuged at 20 °C for 15 min. The eluates were discarded; 100 μL of buffer 1 was pipetted into the filtration unit, and the units were centrifuged again. Flow-through from the collection tube was discarded, and a reduction buffer of 100mM TCEP in buffer 1 was added to achieve a final concentration of 10mM and incubated for 30 minutes at room temperature. Then, 50 μL of 0.05 M iodoacetamide in buffer 1 was added to the filters, and samples were incubated in darkness for 20 min. Filters were washed twice with 100 μL of buffer 1 followed by two washes with 100 μL of 25mM ammonium bicarbonate, pH 8.5 (digestion buffer- buffer 2). 2.5ul of LysC (0.2 µg / µl) in 50 µL buffer 2 to filter and incubate for 4 h at 37ºC. 10 µL trypsin (0.1 µg / µl) in 300 µL buffer 2 was added to the filter and incubated overnight at 37ºC. Recovered peptides were filtered and collected in a separate tube. Peptides were purified on C18 ZipTip columns.

2 ul of resulting tryptic peptides were analysed on a NanoAcquity-UPLC system (Waters) connected to a Q-Exactive HF Hybrid Quadrupole-Orbitrap mass spectrometer possessing an EASY-Spray nano-electrospray ion source (Thermo Fischer Scientific). The peptides were trapped on an in-house packed guard column (75 μm i.d. x 20 mm, Acclaim PepMap C18, 3μm, 100 Å) using solvent A (0.1% Formic Acid in water) at a pressure of 140 bar. The peptides were separated on an EASY-spray Acclaim PepMap® analytical column (75 μm i.d. × 50 mm, RSLC C18, 3 μm, 100 Å) using a linear gradient (length: 90 minutes, 3 % to 60 % solvent B (0.1% formic acid in acetonitrile), flow rate: 300 nL/min). The separated peptides were electrospray directly into the mass spectrometer, operating in a data-dependent mode using a CID-based method. Full scan MS spectra (scan range 350-2000 m/z, resolution 120000, AGC target 1e6, maximum injection time 250 ms) and subsequent HCD MS/MS spectra (AGC target

5e4, maximum injection time 100 ms) of 10 most intense peaks were acquired. HCD fragmentation was performed at 35 % of normalised collision energy, and the signal intensity threshold was kept at 500 counts.

Data Processing- Protein identification

Rationale -The protein analysis aimed to discover *B. bacteriovorus* proteins present at significant levels in the vesicle enriched preparations, which contained both predator and prey (*E. coli* or *P. putida*) materials, as they derived from invaded prey bdelloplasts. This proteomic approach was not an endpoint identification in itself. It was used to generate a list of potential candidate *B. bacteriovorus* proteins, which could then be tagged with mCherry and tested for cellular location microscopically to see if they did indeed reside in the vesicle *in vivo* during predation.

Data Processing -The analysis was performed with Peaks 8.5 software (Bioinformatics Solutions Inc). The raw MS file was first searched against the whole UniProt Database. LysCTrypsin with a maximum of 3 missed cleavages and one unspecific end was selected as the protease. Carbamidomethylation (Cysteine) was set as a fixed modification, and Oxidation (Methionine) and Deamination (Asparagine, Glutamine) were set as variable modifications. Precursor mass tolerance was set as 10 ppm. Fragment mass tolerances for HCD were set to 0.02 Da, respectively. All spectra were manually validated. All peptides present at -10lgP> 20, and spectra were manually checked, validated, or disqualified. PEAKS DB measures the quality of the Peptide-Spectrum Match (PSM) internally with a Linear Discriminative Function (LDF) score. To identify the protein, LDF considers the matching of fragment ions and spectrum peaks and the similarity between the de novo sequencing peptide and the universal database peptide, amongst other factors. The LDF score is converted to -10lgP to facilitate assessment. Peptides present at a p-value of 1% were selected for PSM validation with a Target Decoy PSM Validator node based on q-values at a 5% false discovery rate (FDR). Only validated peptides were used in protein database searches. A PEAKS PTM search was performed after a PEAKS DB search finished. PEAKS PTM analyses spectra with good de novo sequences that remain unidentified by PEAKS DB. The default setting for PEAKS PTM is to search with all the built-in modifications in the "Common" and "Uncommon" lists (more than 300 in-built modifications), which include all the natural modifications and mutations in the Unimod database.

To further investigate the protein associations, filtered data searches were conducted using Uniprot bacteria, the *E. coli* database and the *Pseudomonas putida* databases (considered prey "contaminant" proteins) and the *B. bacteriovorus* HD100 database for proteins from the predators.

The raw data are presented in Supplementary data. **Supplementary Figure 9A** shows the numbers of proteins identified as prey or *Bdellovibrio* in each prep. **Supplementary Figure 9B** shows the overlap in the datasets and identifies a core of 65 proteins in common between the two enrichment methods we took to be vesicle protein candidates for testing (by mCherry tagging and microscopy). These data are presented in the "overlapping lists" tab in Supplementary data. The datasets had many contaminating prey proteins, as expected. Some differences between the *Bdellovibrio* protein candidates found in the datasets were likely due to the use of a modified protocol (as detailed in gradient vesicle enrichment section above) for vesicles from predation on *E. coli* data set 1 versus that used for the vesicle enrichment from predation on *E. coli* data set 2 & predation on *P. putida* dataset.

We didn't seek to resolve these differences, because we were using the process to identify putative *Bdellovibrio* protein candidates for validation via experimental testing, not as an experimental endpoint. We noted a strong enrichment (14 of the 65 proteins) of a group of proteins annotated as "cell wall anchor/YapH-like/phage-related tail fibre" proteins and concentrated attention on this group.

Supplementary Note 2- fluorescent tagging of MAT proteins

Three initial sampled (“cell wall anchor/YapH-like/phage-related tail fibre”) proteins, from the vesicle proteomes: Bd3182, Bd2133, and Bd2439, were labelled by gene fusion with a fluorescent C-terminal tag and tested for fluorescence location in the bdelloplast alone (Supplementary Data Fig. 10), and in combination with CpoB Bd0635 tagged with a different coloured fluorophore to test for vesicle co-location (Fig. 2A and Supplementary Data Fig. 11). Bd2133 and Bd2439 were found to locate in the same deposited vesicle, in the bdelloplast, as contained fluorescently tagged Bd0635 (CpoB) from 30-180 minutes of predation (Fig. 2A and Supplementary Data Fig. 11-12). Sequence examination of Bd2133-mNeonGreen strain showed that a natural mutation I888T had arisen in the fluorescently tagged variant and that the original I888 version did not permit a fluorescent tag. An mCherry tag was not tolerated by Bd2133, so we used a C-terminal mNeonGreen tag to follow the localisation of this protein. Other tested proteins with fluorescent tags discussed in this study permitted C-terminal fluorescent tags without mutation.

Fluorescence from S74 protein Bd3182-mCherry was not seen deposited in the predatory vesicle, but as a bright separate focus of Bd3182-mCherry fluorescence deposited elsewhere distally from CpoB in the prey bdelloplast, and only clearly seen at a later stage, approximately 120-180 minutes into predation (red focus, Fig. 2A Supplementary Data Figs. 11 and 15). In a Bd3182(S782A)-mCherry strain (Supplementary Data Fig 15), engineered to substitute the active site serine residue (proteolysis activity via Ser782 can be confidently predicted from UniProt annotation, and is confirmed in our structural studies herein), *in-vivo* fluorescent mCherry foci were scarcely detectible. These data suggest that S74-mediated cleavage of the chaperone domain happens around 120-180 minutes into predation to allow access to the bdelloplast binding site of Bd3182 only at this point and serendipitously allows unrestricted fluorescence of the chaperone-bound mCherry. Linkage of the mCherry tag at the C-terminal

end of the chaperone infers that we follow the released chaperone domain in the wildtype S74 MAT proteins. This potential complication is not an issue for the NS74 MAT members as they do not have the protease site to allow auto cleavage.

We expanded our fluorescent tagging and microscopic experimentation into other protein family members that had not come up in the proteome of our initial vesicle preparation, mindful that the relative level of expression of "cell wall anchor/YapH-like/phage-related tail fibre" proteins was only being tested proteomically at the end of predation (300 minutes and later into predation) by our sampling of vesicles in the lysed prey debris. Base strain and plasmid details are shown in Supplementary Table 7.

NS74 proteins Bd1334, Bd2734 and Bd2740 were chosen as they represented different protein family subgroupings, with potentially different functionalities (as identifiable from differing C-terminal folds at the end of the fibre, details of these are provided in the main text). We found that Bd2740mCherry did locate to the $CpoB_{Bd0635}$-containing vesicle, deposited during predation, while Bd1334mCherry and Bd2734mCherry fluorescence was diffuse throughout the *B. bacteriovorus* cell (Fig. 2A and Supplementary Data Fig. 11). Frequencies of co-localisation of Bd2133, Bd2439 and Bd2740 with CpoB throughout the timecourse of predation are presented in Supplementary Data Fig. 12.

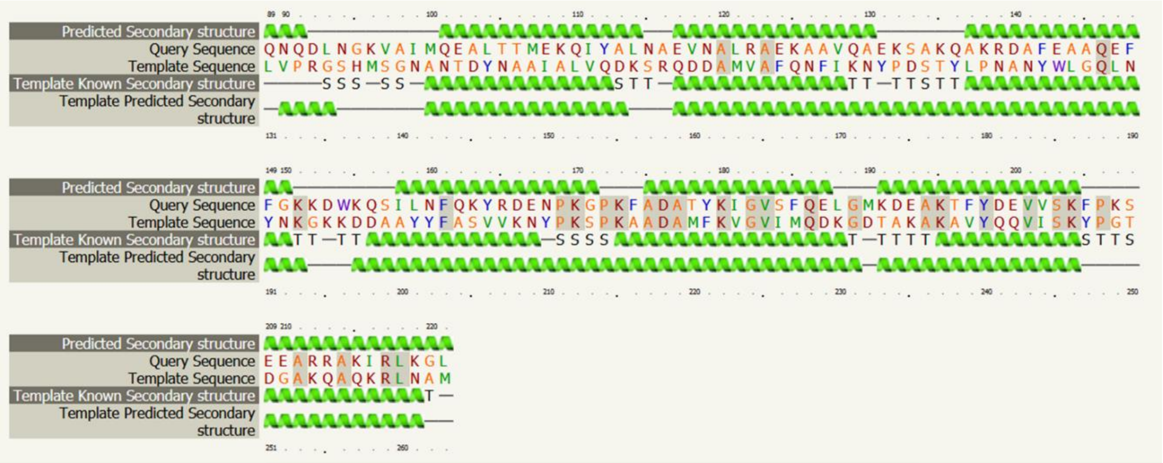

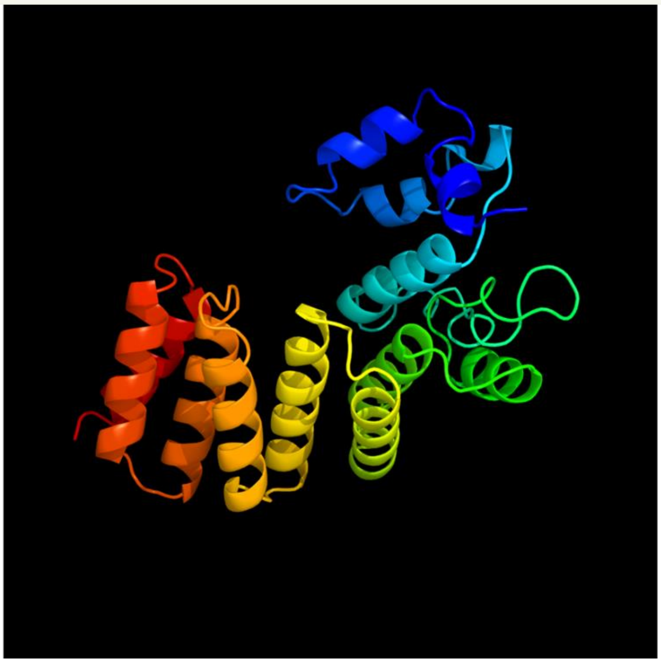

Supplementary Figure 1. Predicted Features of *Bdellovibrio* CpoB Phyre2 output shows that the predicted structure of CpoB from *B. bacteriovorus* HD100 (query sequence) is highly conserved and maps to the *E. coli* K12 CpoB structure (template sequence) with a high confidence score (99.8). Alpha helices are in green, T is a hydrogen-bonded turn, and S is a bend.

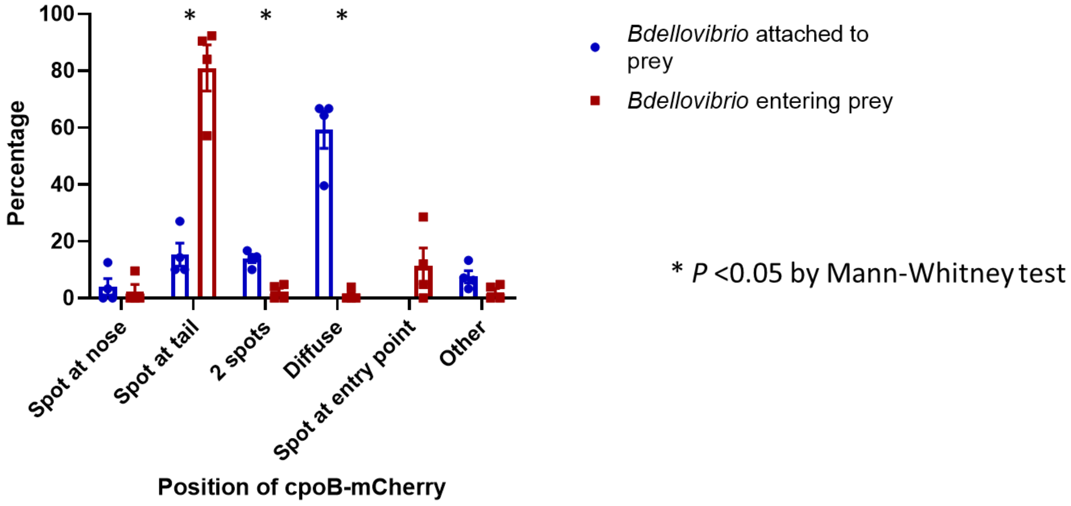

Supplementary Figure 2. Distribution of CpoB-mCherry patterns of *B. bacteriovorus* HD100 attached to prey cells compared to those entering prey cells. Four independent data points were obtained by using 15 min and 20 min timepoint data from two independent experiments. Means and independent data points are presented. Error bars are SEM. * denotes significance p<0.05 by the two-tailed Mann-Whitney test Exact P = 0.286 for all three.

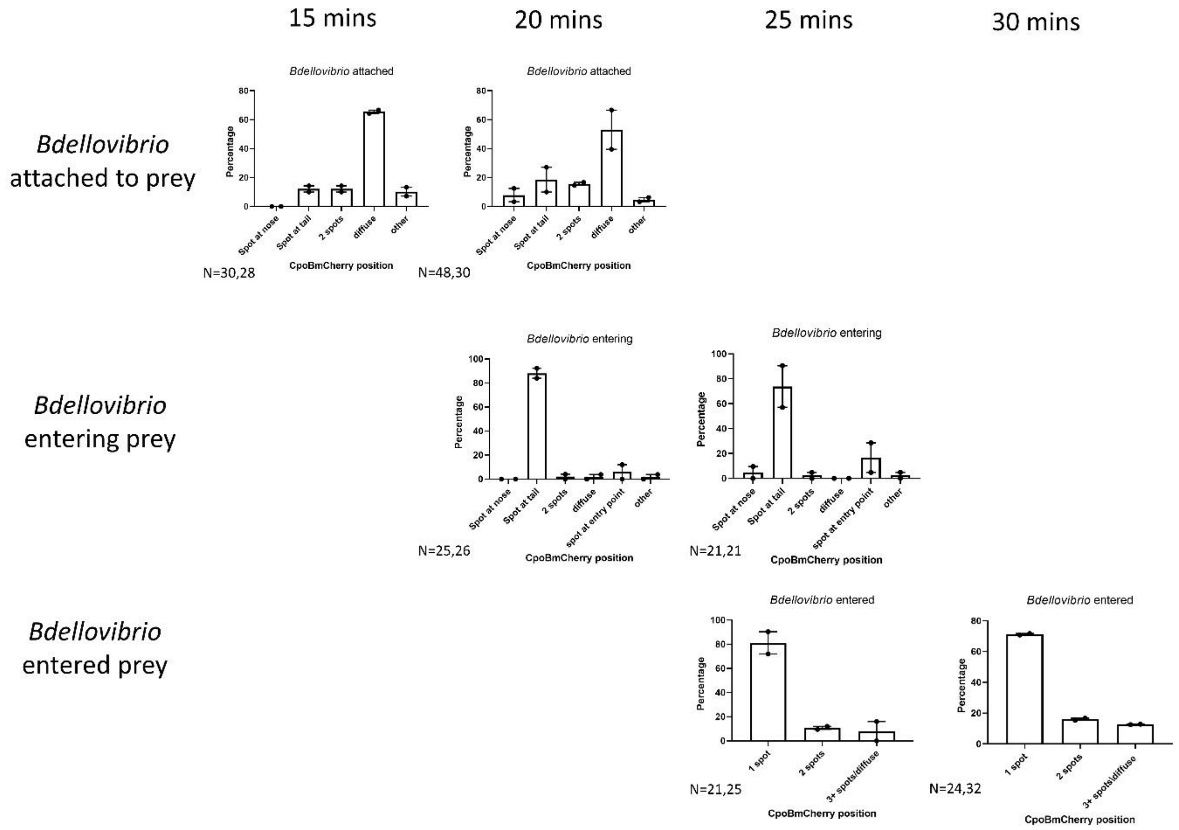

Supplementary Figure 3. CpoB-mCherry Distributions. Distribution of fluorescent CpoB-mCherry patterns in wild type *B. bacteriovorus* HD100 attached to (15-20 min), entering (20-25 min), or fully entered (25-30 min) into prey cells. Means and data points of two independent experiments are presented. Error bars are SEM. Values of n (Bdellovibrio cells analysed) are shown for each experiment and time point.

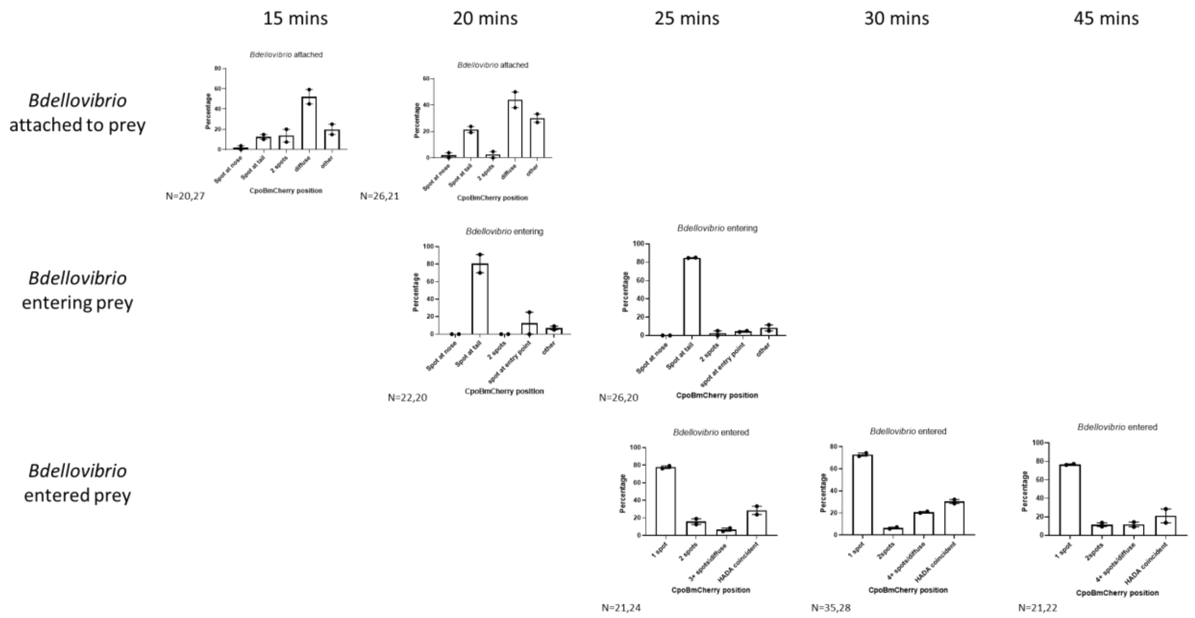

Supplementary Figure 4. Distribution of fluorescent CpoB-mCherry patterns in mutant *B. bacteriovorus Δbd0886Δbd1176* (double LD-transpeptidase deletion mutant background). Mutant strain attached to (15-20 min), entering (20-25 min), or fully entered and establishing within the bdelloplast (25-45 min) prey cells. These data are to validate the use of this strain versus data in Supplementary Figure 3 for wild-type *B. bacteriovorus* (in Supplementary Figure 5) to show up the HADA-labelled porthole in a bdelloplast background with less additional lateral incorporation of other D-amino acids by LD-transpeptidation). Means and data points of two independent experiments are presented. Bdelloplasts were scored for when a focus of CpoB-mCherry spot appeared to be co-localised with a HADA spot (HADA coincidence). Error bars are SEM. Values of n (Bdellovibrio cells analysed) are shown for each experiment and time point.

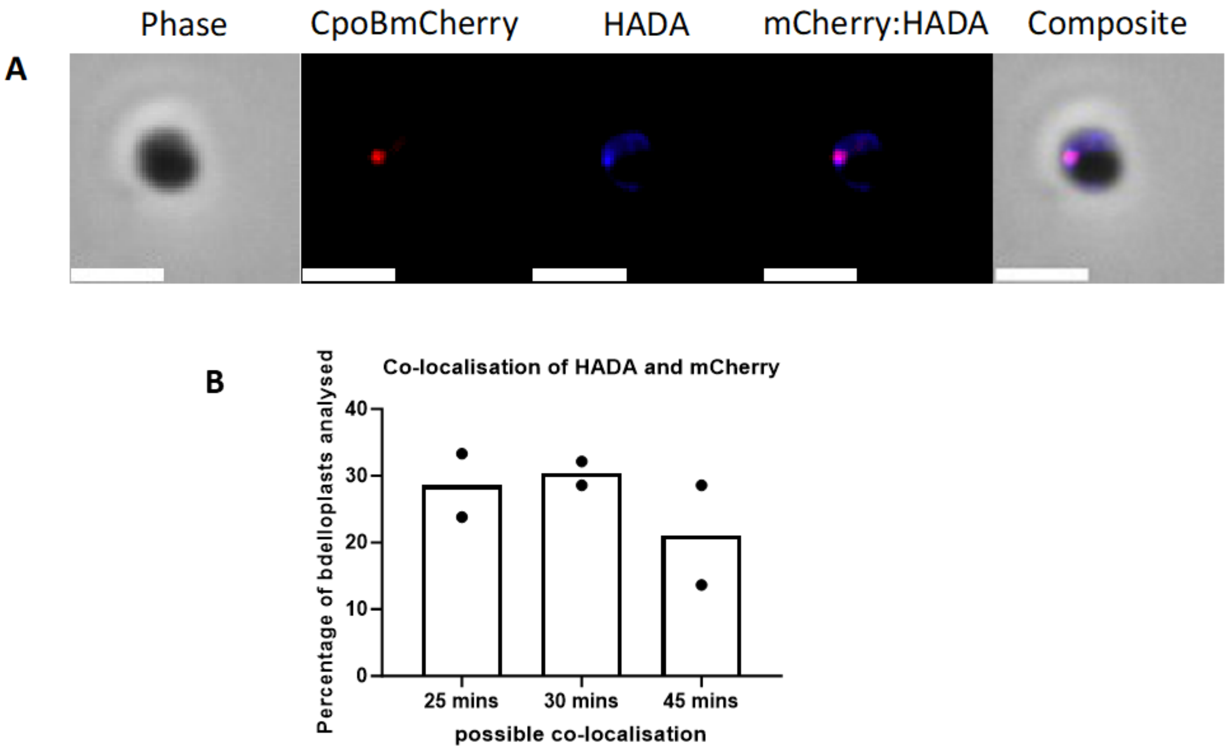

Supplementary Figure 5. CpoB: HADA Co-localization. A Phase and epifluorescence microscope images demonstrating a fluorescent CpoB-mCherry focus (red) co-locates in the same place as the HADA “porthole” focus (blue) in the wall of a bdelloplast formed by predation by *B. bacteriovorus Δbd0886Δbd1176* (CpoB-mCherry) and pulse-labelled with HADA. Individual phase and fluorescence channels and merges are presented. Images are representative of two independent experiments. Scale bars are 2 µm. **B** Plot of the number of bdelloplasts analysed in two independent experiments that displayed a focus of CpoB-mCherry in the same position as a focus of HADA. Means and independent data points are presented.

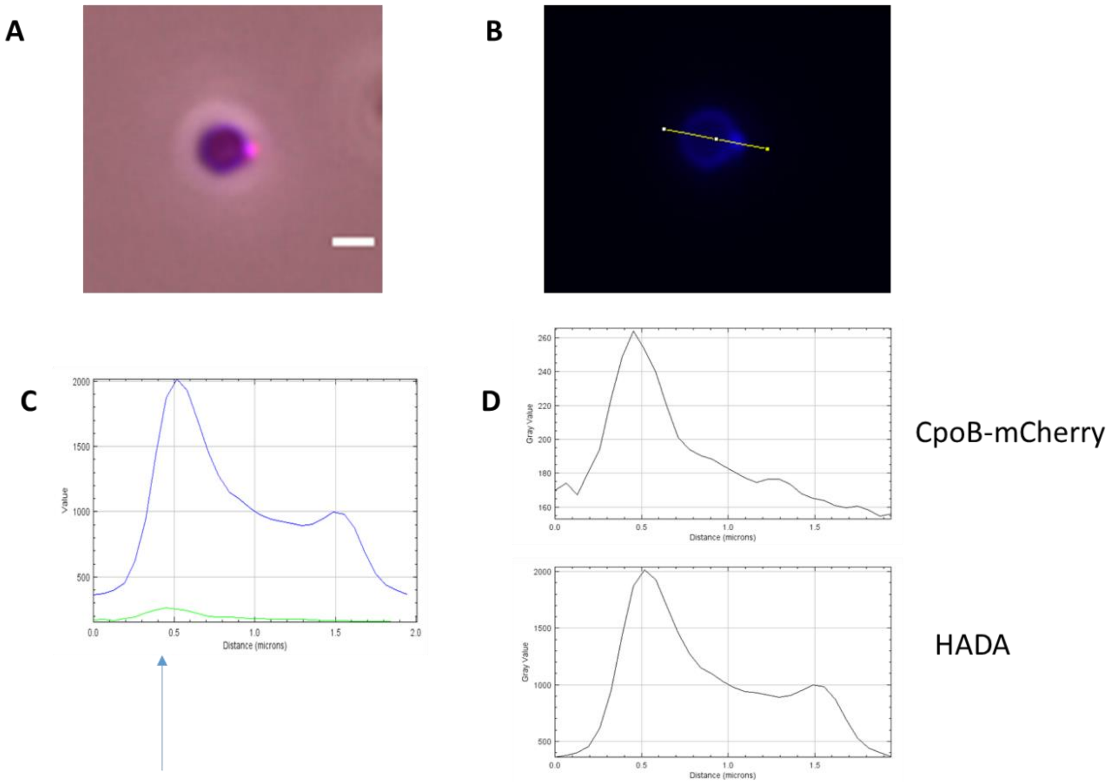

Supplementary Figure 6. Bdelloplast CpoB-mCherry: HADA Relationship. A Composite phase and epifluorescent microscope image showing a bdelloplast with co-incident CpoB-mCherry and HADA foci. The scale bar is 1 µm. **B** Line drawn through the bdelloplast traversing the co-incident foci. **C** Plot of fluorescence values along the line drawn in B showing co-incident peaks (arrow) of CpoB-mCherry (green) and HADA (blue). **D** Plots, as in C, scaled individually. An example typical of 40 analysed bdelloplasts is shown from two independent experiments.

1- a ROI around the whole bdelloplast

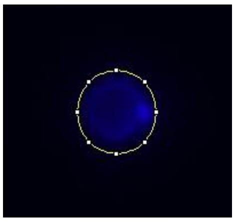

R= 0.82
P= 1.0

2- a ROI around the spot

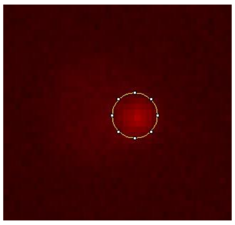

R= 0.66
P= 0.95

3. A control of the bdelloplast circle moved to a region of background was also tested

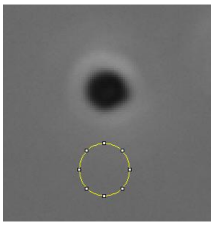

R= 0.22
P= 0.78

Pearson's image correlation coefficient R and averaged Costes P calculated by 1000 rounds of Costes randomisation:

	**Whole bdelloplast**		**Spot only**		**Control**	
	**R**	**P**	**R**	**P**	**R**	**P**
**25 mins**	0.68±0.09	0.99±0.009	0.71±0.14	0.92±0.07	0.09±0.09	0.47±0.43
**Total n**	13	13	13	13	13	13
**N for P>0.95**	13	13	7	7	0	0
**30 mins**	0.71±0.09	0.99±0.006	0.72±0.14	0.95±0.05	0.12±0.11	0.66±0.12
**Total n**	18	18	18	18	18	18
**N for P>0.95**	18	18	13	13	0	0
**45 mins**	0.68±0.09	0.99±0.01	0.72±0.12	0.93±0.06	0.06±0.08	0.52±0.22
**Total n**	9	9	9	9	9	9
**N for P>0.95**	9	9	5	5	0	0

Supplementary Figure 7. Co-localisation Analysis for HADA and CpoB mCherry protein fluorescence. Analysis was carried out using the coloc2 plugin for ImageJ by either choosing an ROI around the whole bdelloplast (1) or an ROI around the CpoB-mCherry focus (2). A control ROI was made by moving the bdelloplast ROI to a nearby background region (3). Coloc2 was run with Manders' correlation algorithm and Costes' significance test with 1000 randomisations. An example typical of 40 analysed bdelloplasts is shown. The table shows the correlation R and significance P for these different analyses. Both methods at all timepoints demonstrated significant colocalisation (P>0.95), but never for the control.

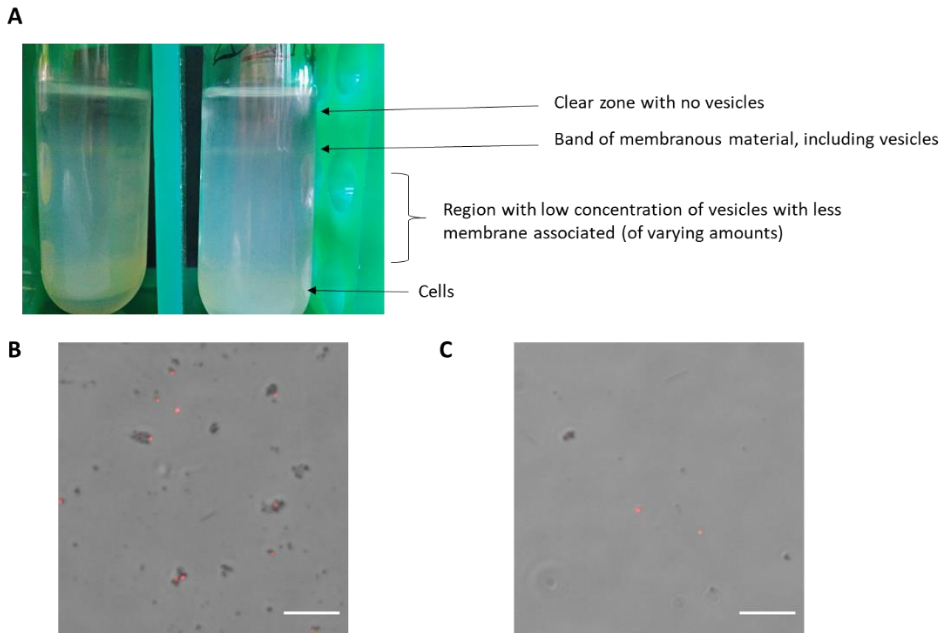

Supplementary Figure 8. Vesicle Enrichment. A Percoll gradient to enrich for predatory vesicles. The remaining cells were pelleted at the bottom of the gradient. **B** Phase and epifluorescence composite of typical band contents visible in Percoll gradient. Pieces of debris are likely to be the remnants of the prey outer layers, and some display a fluorescent predatory vesicle within. **C** Phase and epifluorescence composite of typical band contents visible in Percoll gradient after 4 rounds of Percoll purification. Fluorescent predatory vesicles are enriched without extensive prey debris, although some of this remains. Scale bars are 5 µm. Images are representative of the vesicles found in three independent enrichments.

A

Prep 1 – Enrichment 1 with *E. coli* prey
Prep 2 – Enrichment 2 with *E. coli* prey
Prep 3 – Enrichment 2 with *P. putida* prey

Prep	1	2	3
Prey hits	664	280	571
Bdellovibrio hits	686	127	563

B

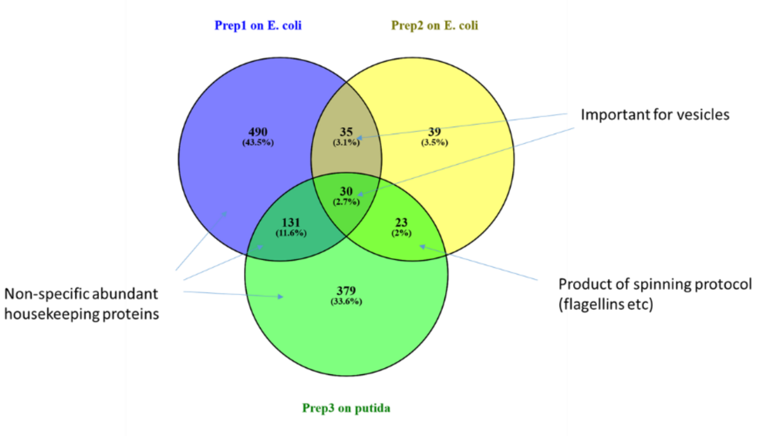

Supplementary Figure 9. Vesicle Proteomics Details. Several proteins identified from prey and *Bdellovibrio* for each prep. **B** Venn diagram showing overlap of proteins identified in the different preps.

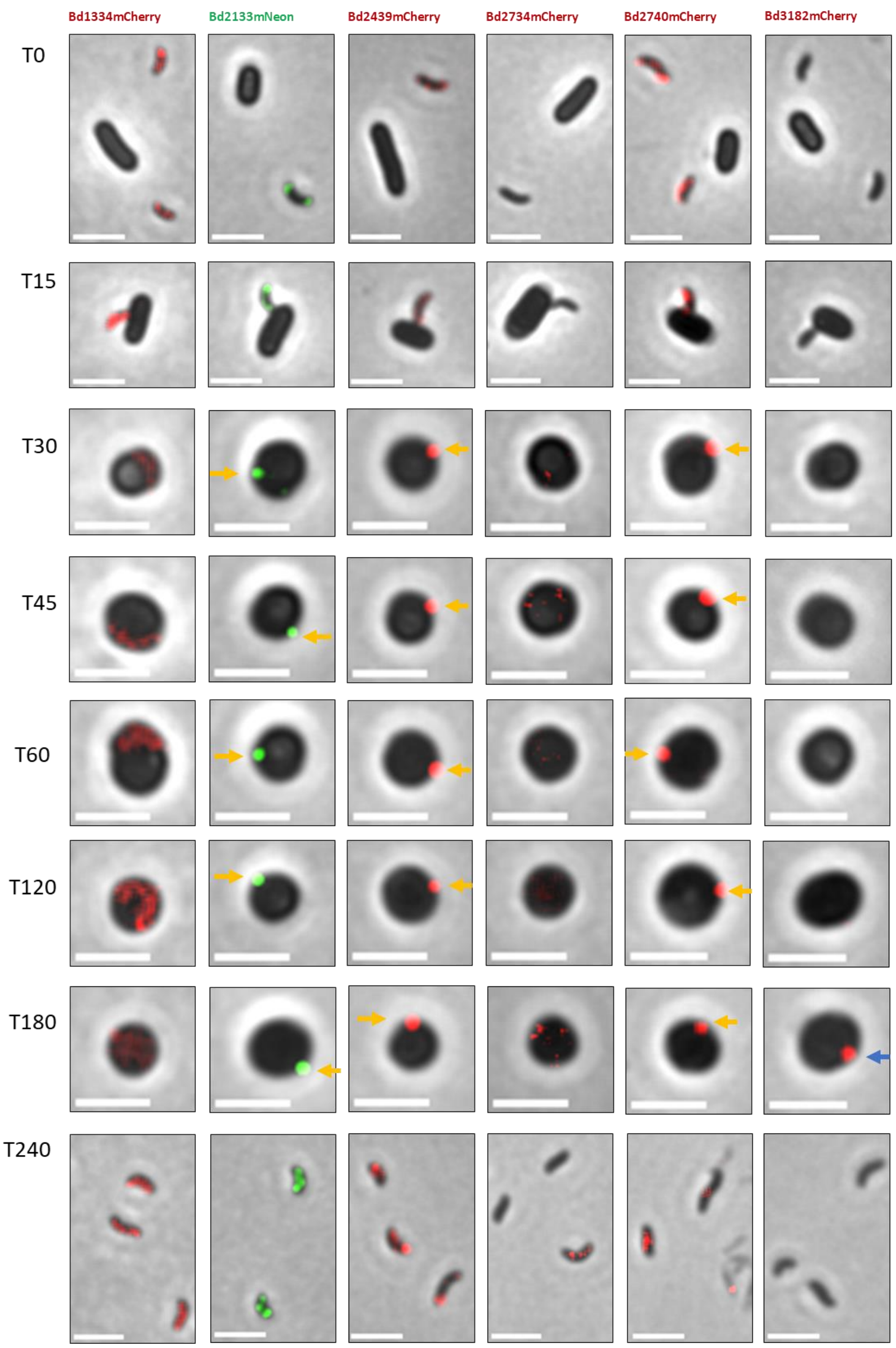

Supplementary Figure 10. Fluorescent MAT protein deposition moving from predator to prey. This is seen in *B. bacteriovorus* HD100, (expressing single MAT proteins fluorescently C terminally tagged), invading, growing inside and exiting *E. coli* S17-1 prey (for Bd2734) or *E. coli* S17-1 (pZMR100) prey for other MAT proteins. Fluorescence is initially seen in predators and then prey bdelloplasts. *B. bacteriovorus* strains express the following chromosomal fusions: Bd1334mCherry, Bd2133mNeon, Bd2439mCherry, Bd2734mCherry, Bd2740mCherry and Bd3182mCherry. Bd2133mNeon (green), Bd2439mCherry and Bd2740mCherry fluorescence (red) can be seen to form in a vesicle in the prey (bdelloplast) wall (yellow arrows) from 30 minutes, which can also be observed left over in prey debris. Bd3182mCherry fluorescence (red) can be seen to form in a vesicle in the prey (bdelloplast) wall from 180 minutes (blue arrow). T = minutes elapsed since predators and prey were mixed. Scale bars = 2 µm. Images are representatives of cells from 3 biological repeats.

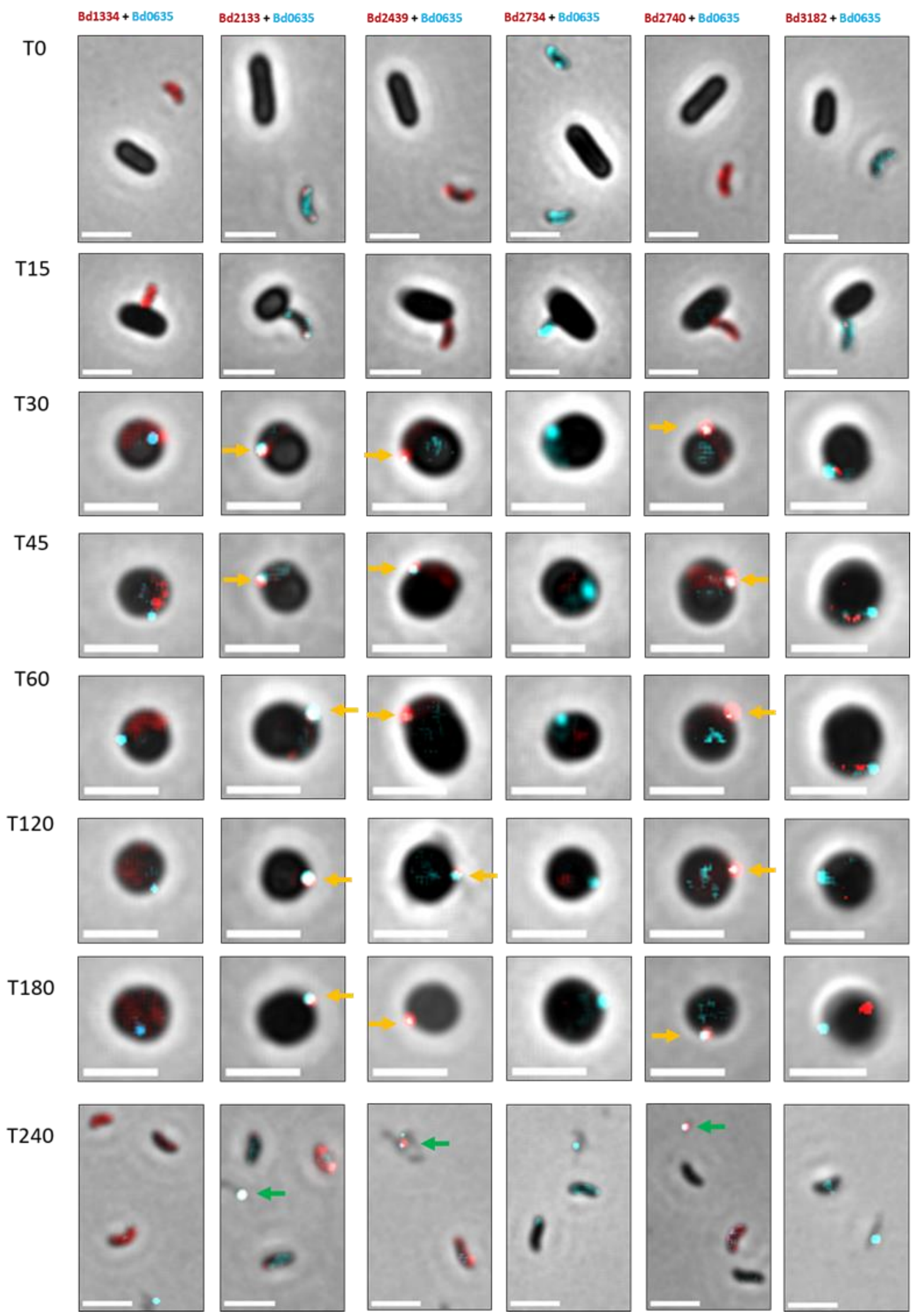

Supplementary Figure 11. Predatory invasion, growth, and eventual prey lysis by *B. bacteriovorus* HD100 strains containing chromosomal fusions of both CpoB$_{Bd0635}$ and MAT proteins to different C terminal fluorescent proteins, inside *E. coli* S17-1 (pZMR100) prey bdelloplasts. Bd1334mCherry with CpoB$_{Bd0635}$mTeal, Bd2133mNeon with CpoB$_{Bd0635}$mCherry, Bd2439mCherry with CpoB$_{Bd0635}$mTeal, Bd2734mTeal with CpoB$_{Bd0635}$mCherry, Bd2740mCherry with CpoB$_{Bd0635}$mTeal and Bd3182mNeon with CpoB$_{Bd0635}$mCherry are shown. For comparison purposes, CpoB$_{Bd0635}$ is false coloured cyan and all MAT proteins false coloured red in all images. For Bd2133, Bd2439 and Bd2740, co-incidence with C-terminally fluorescently tagged CpoB$_{Bd0635}$ is observed as a white spot, and indicated by a yellow arrow, and can also be observed in prey envelope debris (grey material), after predation was completed (green arrows). T = minutes elapsed since predators and prey were mixed. Scale bars = 2 µm. Images are representatives of cells from 3 biological repeats.

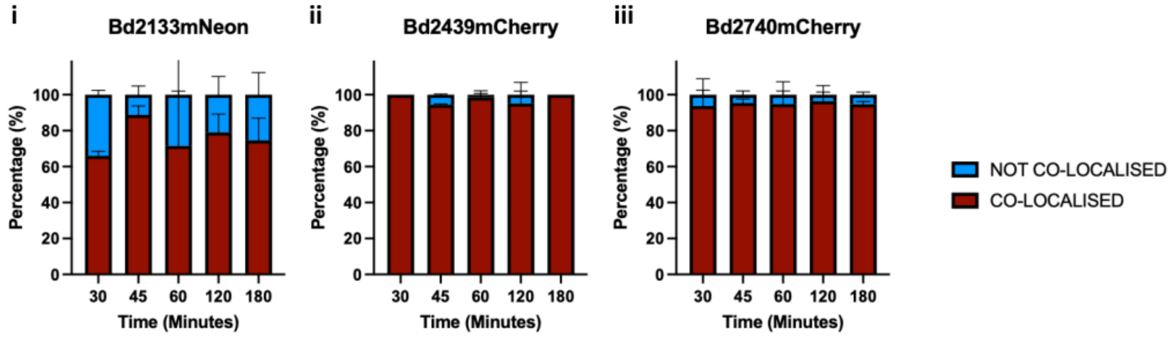

iv

Time (mins)	Bd2133mNeonCpoB$_{Bd0635}$mCherry		Bd2439mCherryCpoB$_{Bd0635}$mTeal		Bd2740mCherryCpoB$_{Bd0635}$mTeal	
	Percentage (%)		Percentage (%)		Percentage (%)	
	Co-localised	Not Co-localised	Co-localised	Not Co-localised	Co-localised	Not Co-localised
30	66% (n=45)	34% (n=45)	100% (n=44)	0% (n=44)	94% (n=47)	6% (n=47)
45	89% (n=54)	11% (n=54)	94% (n=36)	6% (n=36)	95% (n=66)	5% (n=66)
60	72% (n=41)	28% (n=41)	98% (n=62)	2% (n=62)	95% (n=65)	5% (n=65)
120	72% (n=62)	28% (n=62)	95% (n=60)	5% (n=60)	96% (n=64)	4% (n=64)
180	66% (n=61)	34% (n=61)	100% (n=59)	0% (n=59)	95% (n=56)	5% (n=56)

Supplementary Figure 12. Co-localisation analysis of Bd2133, Bd2439 and Bd2740 protein tag fluorescence with CpoB$_{Bd0635}$. Fluorescent foci of tagged Bd2133, Bd2439 and Bd2740 proteins co-localise with CpoB$_{Bd0635}$ foci within bdelloplasts between 30 and 180 minutes of predation. Individual bdelloplasts were selected for the presence of a CpoB$_{Bd0635}$ focus (CpoBmCherry: Bd2133mNeon or CpoBmTeal: Bd2439mCherry and Bd2740mCherry) and correlated with the co-incident expression of a Bd2133mNeon, Bd2439mCherry or Bd2740mCherry focus. The percentage of Bd2133mNeon (i), Bd2439mCherry (ii) or Bd2740mCherry (iii) foci co-incident with CpoB$_{Bd0635}$ foci are presented with standard deviation and values of n (number of bdelloplasts scored). Error bars are standard deviation (iv). Data are from two biological repeats. Images were analysed using ImageJ software (Fiji), using the cell counter plug-in. Adjustments to brightness and contrast for whole images were made until CpoB$_{Bd0635}$ (mCherry: Bd2133mNeon or mTeal (Bd2439mCherry or Bd2740mCherry) and Bd2133mNeon, Bd2439mCherry or Bd2740mCherry foci were visible. Bdelloplasts were then manually (visually) scored for the presence of a CpoB$_{Bd0635}$ focus. Of the bdelloplasts that contained a CpoB$_{Bd0635}$ focus, the number of bdelloplasts for which a Bd2133, Bd2439 or Bd2740 focus was co-incident was scored.

A

Protein	Bd1334	Bd2439	Bd2734	Bd2740	Bd3182
Percentage	21.77 ± 4.2	32.7 ± 7.7	3.5 ± 2.1	35.0 ± 8.1	12.5 ± 5.7
n	9577	6253	7334	5580	6457

B

	Immunofluorescence position				
Protein	None	Anterior	Posterior	Middle	Flagellum
Bd1334	168	7	0	0	7
Bd2439	130	3	2	3	1
Bd2734	798	6	2	0	2
Bd2740	170	18	16	6	4
Bd3182	446	0	0	0	0

Supplementary Figure 13. MAT Protein FITC- anti-mCherry Immunofluorescence Detection Statistical Analysis. A Percentage of attack phase *B. bacteriovorus* cells with detectible external immunofluorescent spot, using the MicrobeJ plugin, and values of n. **B** position and number of immunofluorescence observations relative to the flagellum in FM46-4 stained cells (as in Fig 2C), which had an observable flagellum.

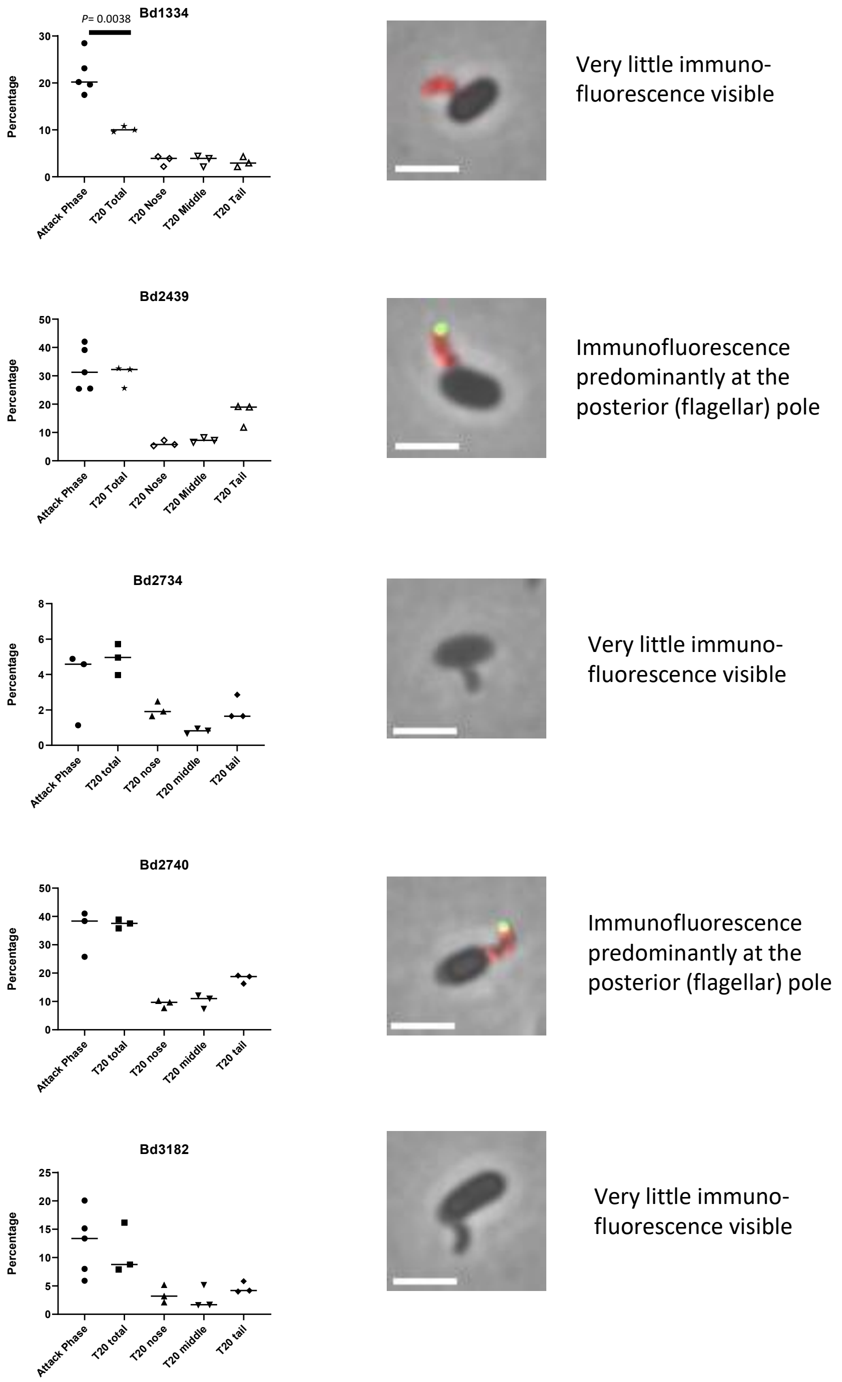

Values of n:

	Bd1334mC	Bd2439mC	Bd2734mC	Bd2740mC	Bd3182mC
**Experiment 1**	271	242	242	314	251
**Experiment 2**	230	344	303	264	241
**Experiment 3**	134	152	105	176	155

Supplementary Figure 14. Positions of extracellular immunofluorescence detected with FITC anti-mCherry on *B. bacteriovorus* cells which are invading *E. coli* prey. Cells attached to prey at 20 minutes post-mixing of predator and prey were manually scored for immunofluorescence spot position. Representative merges of epifluorescence and phase are shown. Scale bars are 2μm. Table indicates the number of cells examined in each experiment. For Bd1334, P = 0.0038 by unpaired, two-sided t-test.

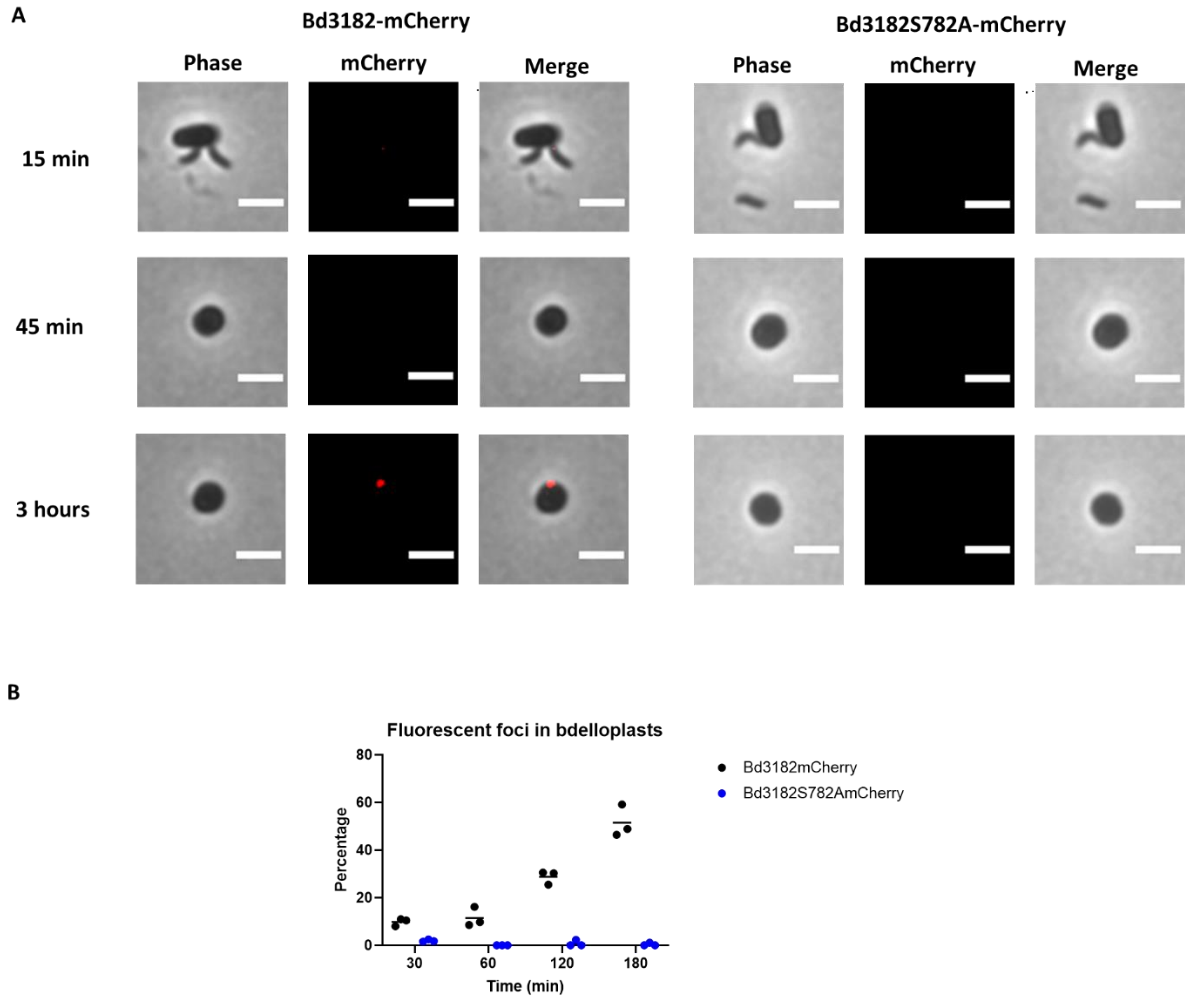

Supplementary Figure 15. Expression of MAT protein Bd3182mCherry *in vivo* inside *E. coli* bdelloplasts. A Phase contrast and epifluorescence microscopy of *B. bacteriovorus* Bd3182-mCherry and Bd3182S782A-mCherry preying upon *E. coli* S17-1. I*n vivo* fluorescence of mCherry is rarely seen in any samples except at the 2 and 3 hour timepoint for the Bd3182-mCherry, where a spot is often visible at the periphery of the bdelloplast. **B** Plot of percentage of bdelloplasts with a visible fluorescence spot of Bd3182-mCherry and Bd3182S782A-mCherry. Values and means from three independent experiments are presented. Scale bars are 1 µm.

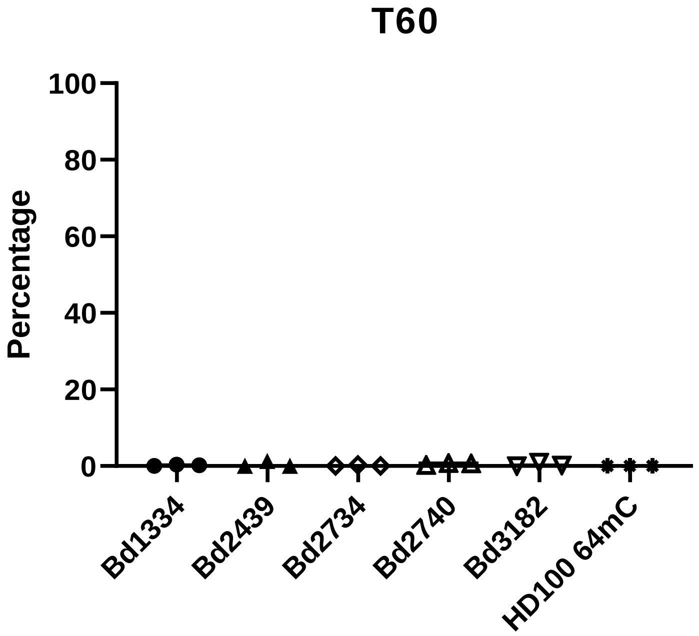

	Bd1334mC	Bd2439mC	Bd2734mC	Bd2740mC	Bd3182mC
Experiment 1	279	242	1184	582	371
Experiment 2	421	506	414	171	445
Experiment 3	174	152	142	498	212

Supplementary Figure 16. FITC Immunofluorescence probing shows no significant mCherry on bdelloplasts containing an invaded predator at 60 minutes post predator-prey mixing. Percentage of bdelloplasts with detectible FITC anti-mCherry immunofluorescence spot at 60 minutes post-mixing of predator and prey and values of n for each experiment. There was no significant difference from the negative control (HD100 with the cytoplasmic protein Bd0064 tagged with mCherry) for any strain by one-way ANOVA. Bdelloplasts and fluorescent foci were detected by the microbeJ plugin. Values of n (number of bdelloplasts detected) are presented in the table for each of three biological repeats.

A

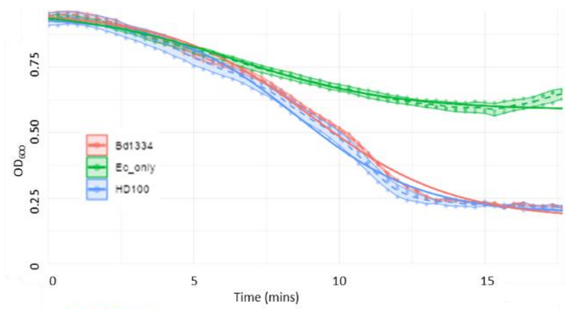

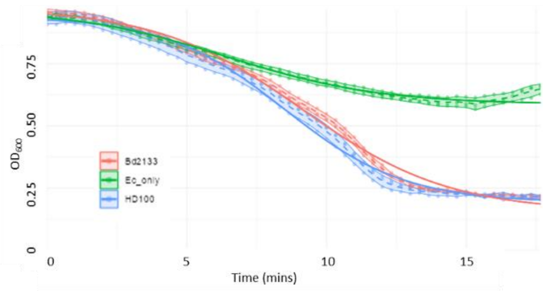

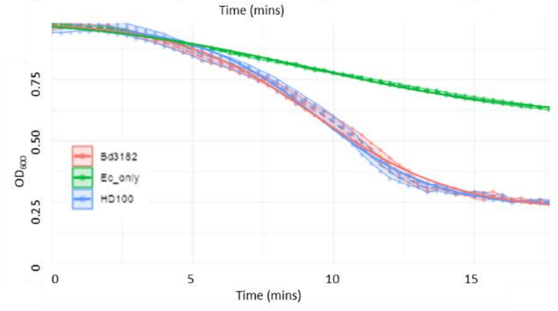

B

(i)

Experiment number	Strain	
	HD100	ΔBd2734
1	94.7% (n=321)	90.1% (n=303)
2	97.5% (n=163)	89.7% (n=146)
3	96.6% (n=235)	93.3% (n=255)
4	97.8% (n=134)	91.4% (n=151)

(ii)

Experiment number	Strain	
	HD100	ΔBd2740
1	97.6% (n=209)	93.1% (n=247)
2	96.3% (n=160)	85.3% (n=129)
3	96.8% (n=155)	94.2% (n=154)
4	96.6% (n=275)	89.4% (n=265)

Supplementary Figure 17. Predation Assays. A Monitoring reductions in prey cell OD_{600} as a result of prey lysis by predator MAT mutants (red) relative to wild-type (blue). Predation by ΔBd1334, ΔBd2133 and ΔBd3182 mutant strains is shown. None of these strains demonstrated a significantly reduced predation rate relative to wild-type. Prey only control is shown in green. **B** Total number of cells counted and percentage of bdelloplasts visually scored for a fully entered *Bdellovibrio* at 30 minutes for each biological repeat for data in Figure 3C-D. Total cells counted (i) HD100 $n = 853$ cells and ΔBd2734 n = 855 (ii) HD100 $n = 799$ cells and ΔBd2740 n = 795 cells.

```
Bd3182 656-669     RVGVGTTAPTSALH
Bd3182 752-765     FFGINTAAPAYNIH
Bd2133 684-697     NLGLGTAAPTSALH
Bd2133 798-811     LVGINTAAPLQKLH
Bd2133 870-883     NVGIGATAPTAKLE
Bd1334 853-866     LVGIGTPAPAVPLQ
Bd1334 911-924     NVGVGVGAPTAKMD
Bd2439 941-954     NVGIGTTTTTEKLN
Bd0884 584-597     NVGIGATSPSAKLQ
Bd0884 666-679     LVGIGTATPAAPLH
Bd0884 784-797     AVGIGTTNPSGILH
Bd0884 894-907     YIGUQADTPRQPLE
Myrf   559-572     RVGINTDRPDEALV
                    .*  .      :
```

Supplementary Figure 18. Alignment of sequences used to create Weblogo (Crooks et al., 2004) for capping loop consensus motif. HD100 MAT identification and AA positions provided; Myrf from *Mus musculus* Isoform 2 of Myelin regulatory factor, PDB 7DC3 (Wu et al., 2021).

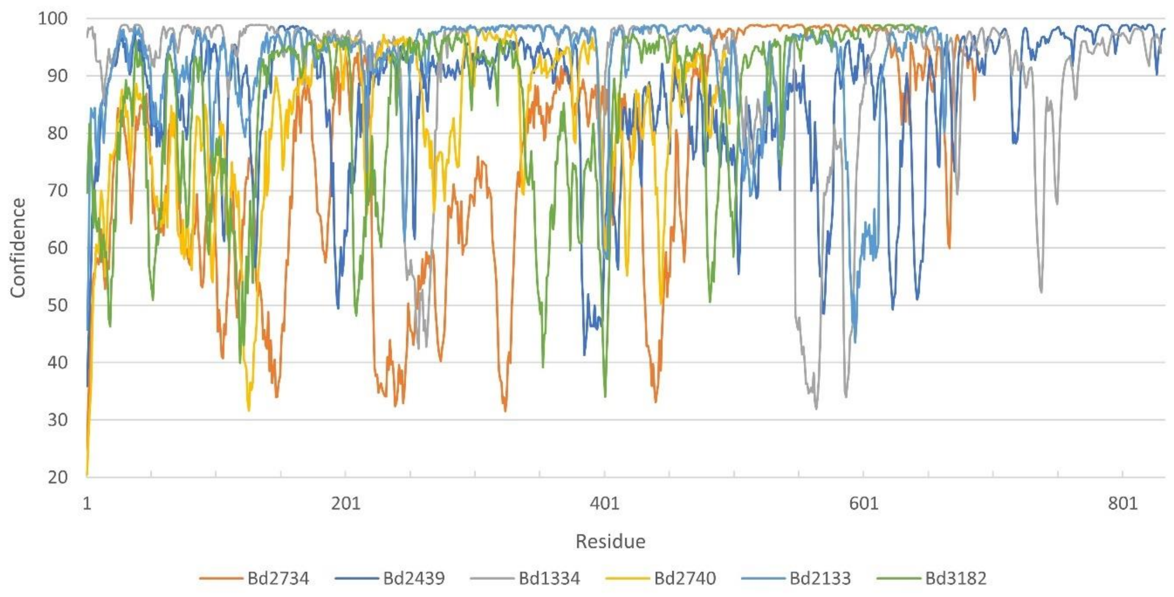

Supplementary Figure 19. Confidence plots of the Collabfold fibre models. Confidence values (direct from Collabfold (Mirdita et al., 2022) output) plotted against residue number for each of the characterized MAT proteins (key below plot).

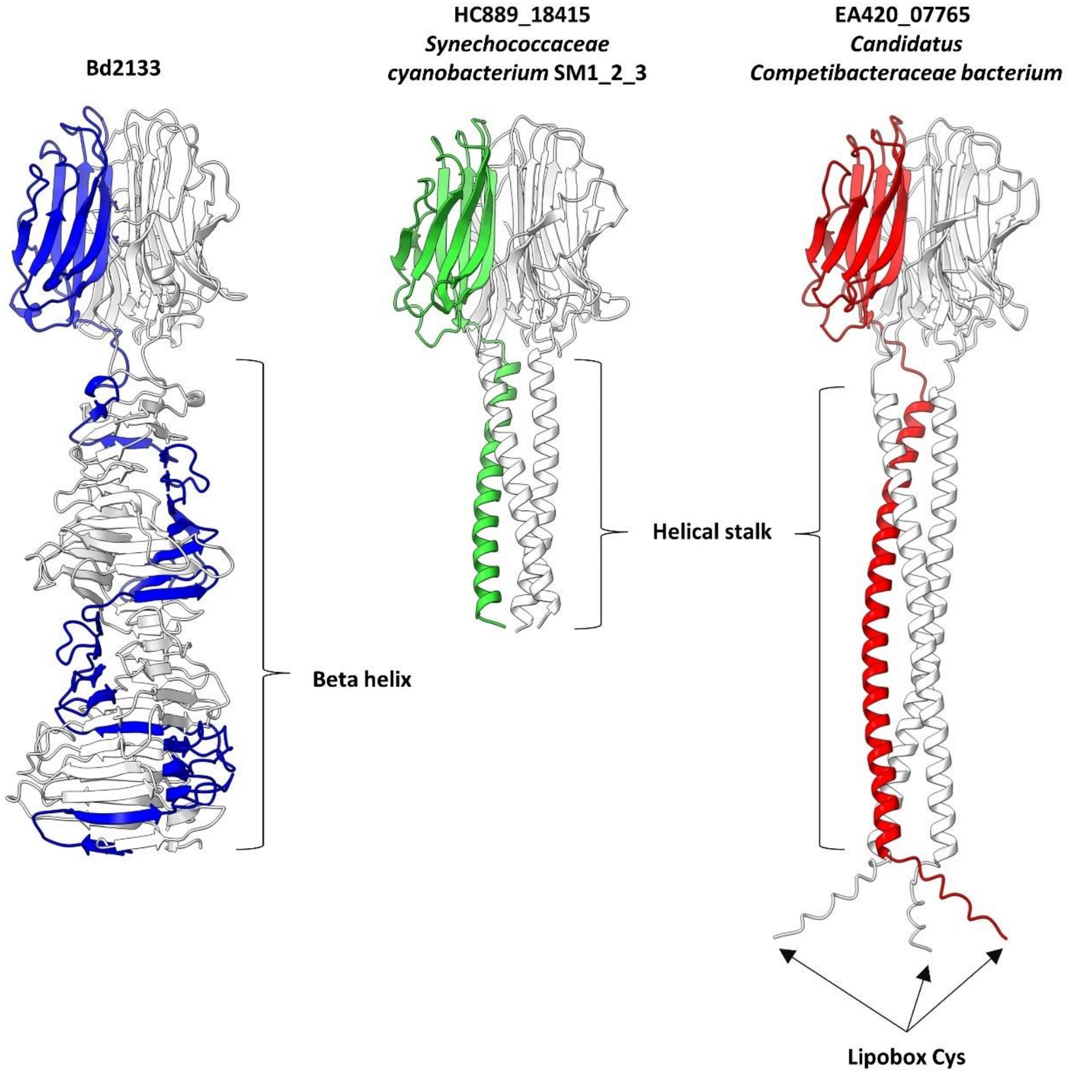

Supplementary Figure 20. Bd2133 Structural Homologues. Comparison of experimental model of Bd2133 (left), with Alphafold (Jumper et al., 2021) models derived from top FoldSeek (van Kempen et al., 2022) hits (left and right) from *Synechococcaceae* and *Competibacter* strains (one chain from each trimer coloured).

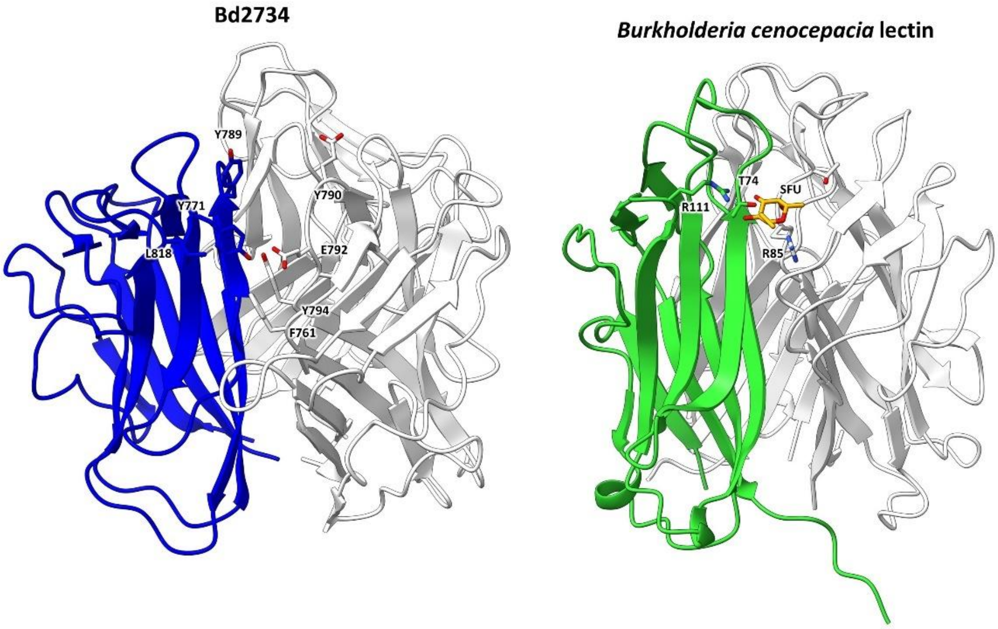

Supplementary Figure 21. **Bd2734 Structural Comparison.** Bd2734 (left) is shown in same orientation as the *Burkholderia cenocepacia* lectin (right; PDB code 2wq4 (Legrand et al., 2016)). The structure of the *Burkholderia cenocepacia* lectin is in complex with methyl 1-seleno-alpha-L-fucopyranoside (SFU), and the same cleft of Bd2734 shows a completely different set of residues.

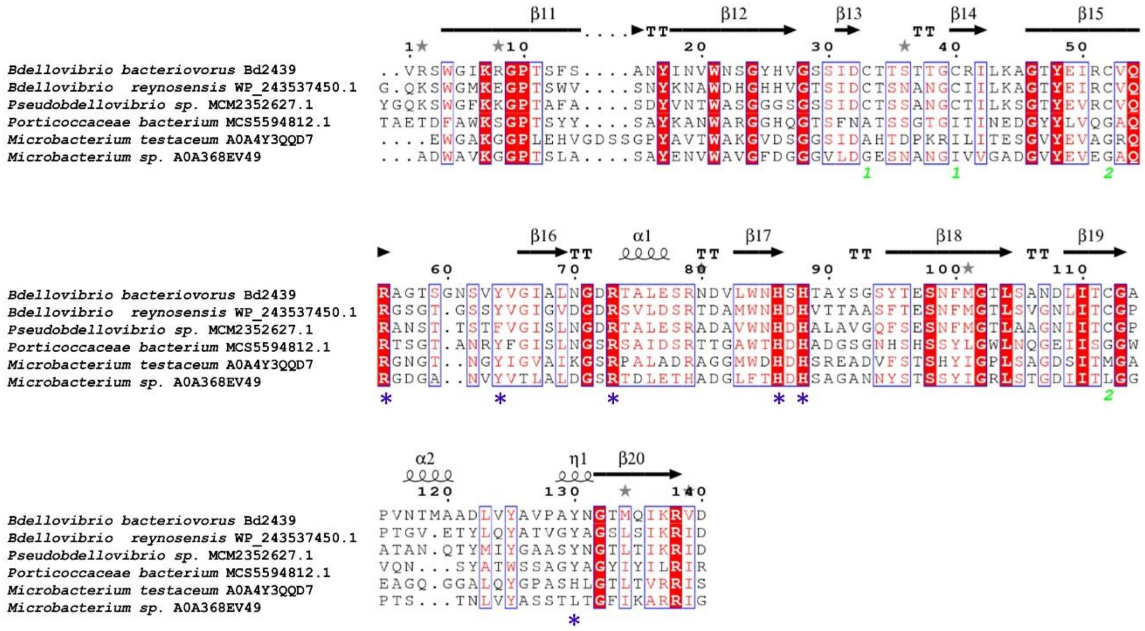

Supplementary Figure 22. Bd2439 Homologue Sequence Conservation. Diverse homologues identified by FoldSeek (van Kempen et al., 2022) were aligned to the crystallised region of Bd2439 using ESPRIPT (Robert and Gouet, 2014). Disulphide-bonding position marked in green, conserved residues in red background and binding pocket residues marked with an asterisk.

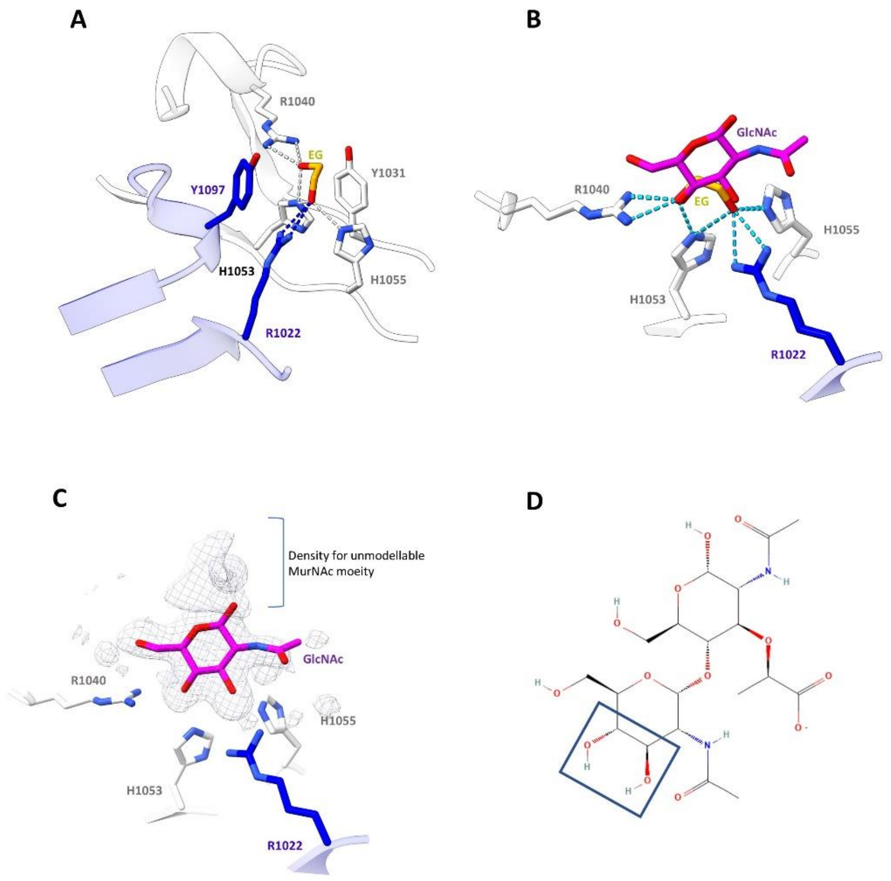

Supplementary Figure 23. Bd2439 ligand binding. A When used as a cryoprotectant, ethylene glycol (EG; orange) occupies the same pocket as GlcNAc-MurNAc. **B** Superposition of GlcNAc (magenta) shows similar binding modes to ethylene glycol (orange). **C** Fo-Fc GlcNAc omit map at 2 σ within 4 Å of GlcNAc. Clear density is observed for the GlcNAc moiety, especially the interacting vicinal hydroxyls. Density is observed above the GlcNAc, which is likely to be the MurNAc moiety. However, this was not modellable, possibly due to the lack of interactions and consequent flexibility. **D** The 2D structure of GlcNAc-MurNAc explains why the GlcNAc moiety can be confidently modelled into the density observed, as there are only two vicinal hydroxyls in the GlcNAc-MurNAc molecule (blue box).

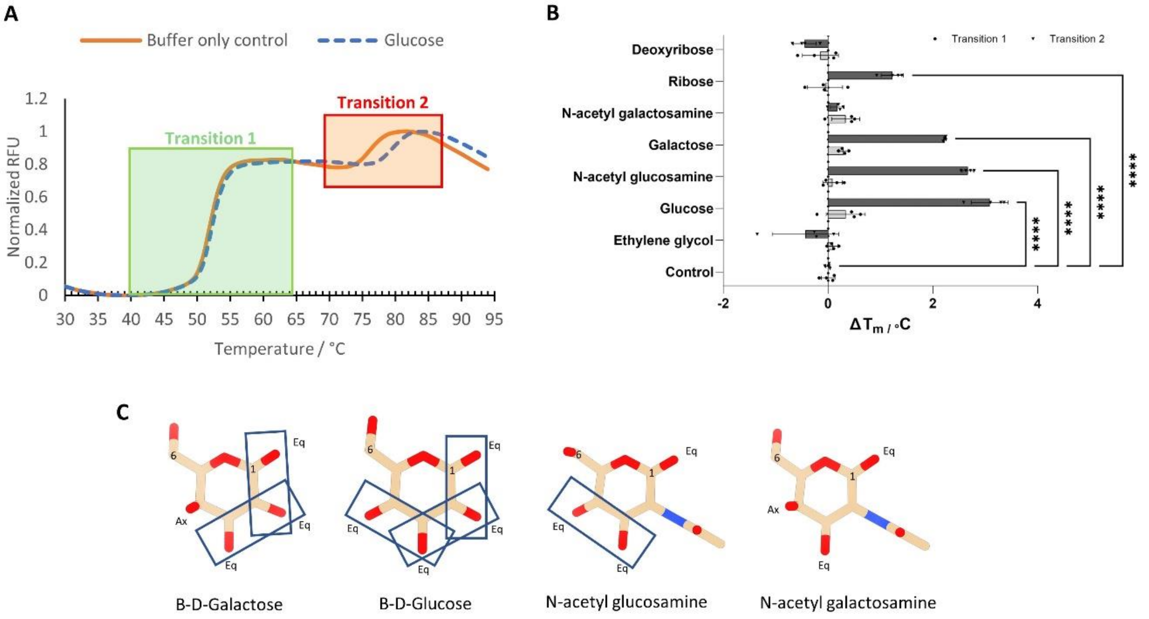

Supplementary Figure 24. Thermal unfolding of Bd2439 in the presence of saccharides. **A** Two normalised thermal unfolding curves of Bd2439. The protein shows a double transition, indicating two unfolding events, with a shift in the temperature at which transition 2 occurs upon the addition of 100 mM glucose. **B** Increases in melting temperature of transition 2 occur upon the addition of 100 mM glucose ($p \leq 0.0001$), N-acetyl glucosamine ($p \leq 0.0001$), galactose ($p \leq 0.0001$) or ribose ($p \leq 0.0001$). One-way ANOVA with Dunnett's test was performed to compare each transition to its control independently using GraphPad Prism 8.0, n=4 technical replicates. Bars show the mean of 4 experiments ± SD. No shift is observed with ethylene glycol, N-acetyl galactosamine or deoxyribose. All transition 1 melting temperatures show non-significant changes from the no sugar control. **C** Bd2439 thermal shift occurs upon binding to pyranoses with vicinal equatorial hydroxyls (blue boxes). The change from an equatorial C4 hydroxyl in N-acetyl glucosamine to axial in N-acetyl galactosamine prevents binding of the latter to Bd2439, as evidenced by similar melting temperatures of transition 2 to the buffer-only control.

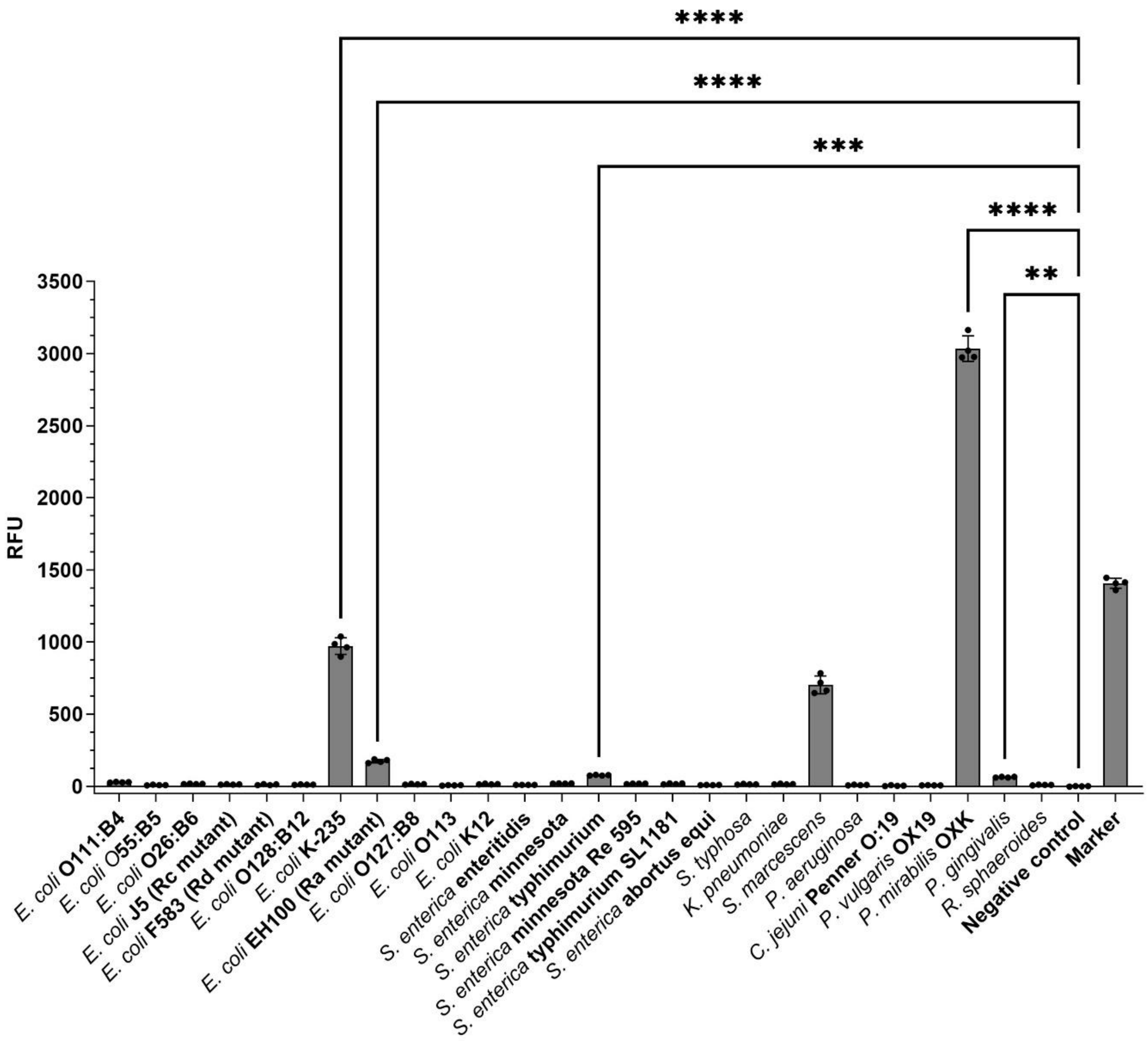

Supplementary Figure 25. Full glycan array screening for Bd2439 showing all 20 species tested. Bd2439 showed binding to *E. coli* K235 (p-value <0.0001), *E. coli* EH100 (Ra mutant) (p-value <0.0001), *S. enterica* Typhimurium (p-value = 0.0006), *S. marcescens* (p-value <0.0001), *P. mirabilis* OXK (p-value <0.0001) and *P. gingivalis* (p-value = 0.0089) by one-way ANOVA with Dunnett's test using GraphPad Prism 8.0, n=4 technical replicates. All other assays show non-significant change from the negative control. Bars show the mean of 4 experiments ± SD.

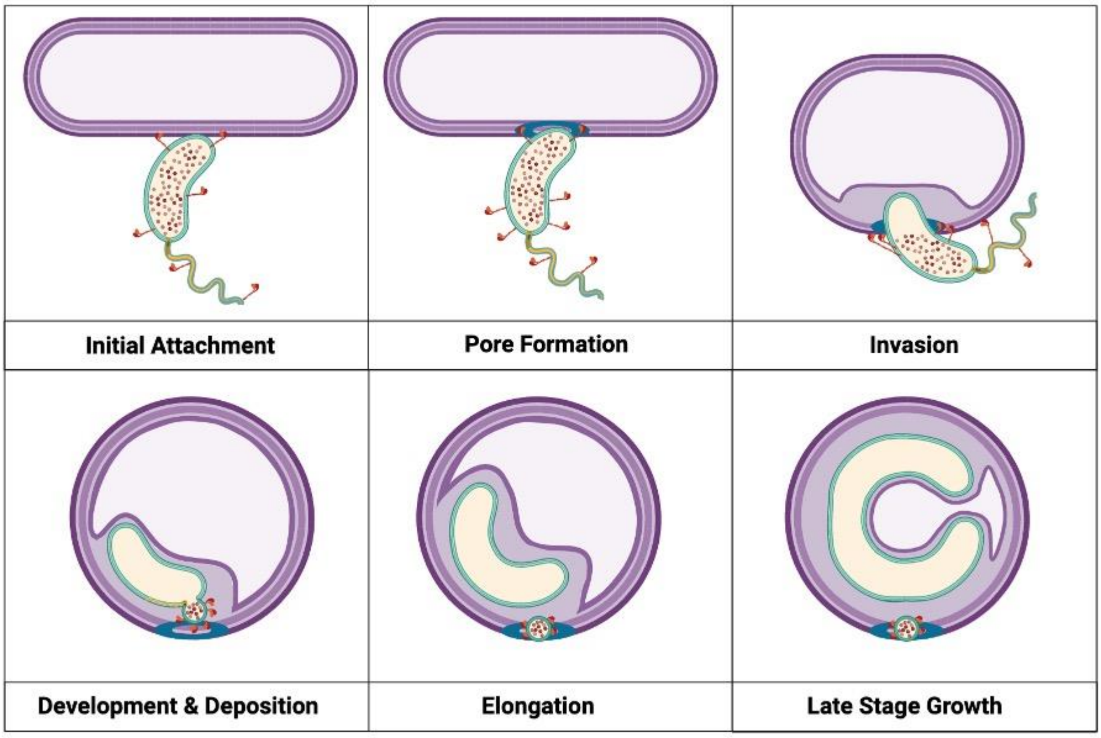

Supplementary Figure 26: **A model for MAT utilization in predation**. The MAT proteins in this study (several associated with an in-prey-deposited $CpoB_{Bd0635}$-containing vesicle), Bd1334, Bd2133, Bd2439, Bd2734, Bd2740 and Bd3182 (shown in red), are surface exposed and localise to the surface of attack phase *B. bacteriovorus* prior to prey encounter, whereby they assist with prey recognition and initiation of predation. To instigate invasion, *B. bacteriovorus* must enter the inner periplasm of the prey cell. This requires attachment to the outer surface of the prey, with subsequent extensive modification of the prey outer envelope layers, the generation of a traversable pore in the peptidoglycan cell wall and outer membrane (represented by a blue disc, indicative of HADA- stained muropeptide amino acid modification). Upon prey attachment, some MAT protein distribution shifts towards the flagellar pole, with the exception of those proteins present on the nose of the *B. bacteriovorus* that aid initial attachment ("Pore Formation": at approximately 20 minutes after predator-prey mixing). *B. bacteriovorus* enters the prey cell, potentially aided by the adhesion of additional MAT proteins, and further modifies the prey cell, filling in the invasion porthole ("Invasion" at approximately 25 minutes). $CpoB_{Bd0635}$, dynamically moves to the "tail pole" of the predator during entry, and predator flagellar reabsorption (yellow spiral, "Development & Deposition") subsequently forming a focus and discrete vesicle compartment that remains associated with the inside of the invasive pore. A subset of the MAT proteins in this study (Bd2740, Bd2439, Bd2133) mirror this shift in distribution, becoming and remaining associated with the $CpoB_{Bd0635}$-containing vesicle ("Development & Deposition": from approximately 30 minutes). This $CpoB_{Bd0635}$-associated vesicle, also containing a subset of MAT proteins, is subsequently deposited at the periphery of the prey periplasm, prior to *B. bacteriovorus* filament growth, again with those MAT proteins facilitating adherence at the now closed entry porthole ("Elongation": approximately 60 minutes; "Late-Stage Growth": approximately 120-180 minutes). This vesicle-closed porthole structure can represent a potential escape hatch for predators if full prey lysis fails at the end of predation, likely due to its different composition [8].

Uniprot ID	Gene Name	Length (AA)	Tip Domain	Notes
Q6MPY4	*bd0704*	1365	S74	Likely mis-annotation of IPR008979
Q6MPY3	*bd0705*	1267	S74	
Q6MPH0	*bd0884*	1148	S74	
Q6MNV0	*bd1130*	1492	S74	
Q6MMI8	*bd1641*	760	S74	Missed start codon in Uniprot
Q6MMC4	*bd1712*	1365	S74	
Q6MLC4	*bd2088*	1258	S74	
Q6MK63	*bd2548*	1660	S74	
Q6MK48	*bd2565*	1164	S74	
Q6MK32	*bd2582*	1416	S74	
Q6MIH5	*bd3182*	922	S74	
Q6MIA1	*bd3266*	1567	S74	
Q6MIA0	*Bd3267*	1507	S74	
Q6MPW3	*bd0726*	1959	ß-rich domain	Structural similarity of tip domain to bacteriophage CBA120 tailspike protein 4 XD1 domain (PDB code 7REJ (Chao et al., 2022))
Q6MNC5	*bd1334*	1151	Novel ß-rich domain	
Q6ML84	*bd2133*	1031	Novel ß-rich domain	
Q6MKF5	*bd2439*	1107	TNF-like	
Q6MJN8	*bd2734*	843	TNF-like	
Q6MJN2	*bd2740*	627	C-type lectin	
Q6MJA9	*bd2872*	626	Novel ß-rich domain	
Q6MHU4	*bd3442*	653	B30.2/SPRY	

Supplementary Table S1. ***Bdellovibrio bacteriovorus*** **HD100 identified MAT proteins.**

	Bd3182$_{632-922}$ (8ONC)	Bd3182$_{632-922}$ (8OJN)	Bd3182$_{632-922}$ (8ONB)	Bd2133$_{662-1031}$ (8OND)	Bd2133$_{910-1031}$ (8OK3)	Bd1334$_{818-1151}$ (8OML)	Bd1334$_{914-1151}$ (8ON4)	Bd2439$_{837-1107}$ with GlcNAc-MurNAc (8OL4)	Bd2439$_{837-1107}$ with ethylene glycol (8ONF)	Bd2734$_{691-843}$ (8OKW)	Bd2740$_{518-627}$ (8OKS)
**Collection statistics**											
**Wavelength**	0.97623	0.97627	0.969991	0.9795	0.9795	1	0.9795	0.979	0.97625	0.9795	0.979
**Resolution range**	79.41 - 2.0 (2.072 - 2.0)	47.47 - 2.51 (2.6 - 2.51)	84.35 - 1.12 (1.16 - 1.12)	42.33 - 2.5 (2.589 - 2.5)	28.07 - 1.501 (1.555 - 1.501)	47.39 - 2.565 (2.657 - 2.565)	31.72 - 1.41 (1.46 - 1.41)	45.13 - 1.841 (1.907 - 1.841)	45.34 - 1.53 (1.585 - 1.53)	45.17 - 1.841 (1.907 - 1.841)	44.11 - 2.094 (2.169 - 2.094)
**Space group**	I 1 2 1	P 1 21 1	P 1 21 1	P 63	P 21 3	C 1 2 1	R 3 :H	P 21 21 2	P 21 21 2	P 1 21 1	P 21 21 21
**Unit cell**	111.457 48.1533 172.20490 107.108 90	93.219 48.133 95.595 90 96.691 90	86.561 48.397 92.964 90 102.984 90	65.3084 65.3084 319.187 90 90 120	68.76 68.76 68.76 90 90 90	196.738 81.863 58.85 90 98.112 90	82.94 82.94 67.67 90 90 120	84.219 160.345 52.075 90 90 90	84.368 161.278 52.067 90 90 90	52.161 87.682 52.754 90 118.881 90	51.084 69.329 87.427 90 90 90
**Total reflections**	366270	251784	1197209	1130646	58792	49730	273126	585270	341050	71987	62661
**Unique reflections**	59589 (5900)	39815 (1992)	176668 (1476)	26645 (2990)	17418 (1691)	14282 (714)	30502 (1320)	49745 (2487)	104485 (9141)	23686 (1184)	12529 (626)
**Multiplicity**	6.1 (5.6)	6.3 (4.7)	6.8 (6.4)	42.4 (42.7)	3.4 (3.4)	3.5 (3.6)	9.0 (3.7)	11.8 (7.0)	3.3 (2.0)	3.0 (1.4)	5 (3.2)
**Completeness spherical (%)**	99.79 (99.58)	78.9 (26.1)	61.3 (11.6)	100.0 (99.7)	98.92 (97.86)	47.5 (7.6)	91.1 (53.0)	80.2 (20.2)	96.9 (79.3)	65.1 (13.9)	64.9 (14.1)
**Completeness ellipsoidal (%)**	N/A	85.8 (45.7)	93.1 (80.9	N/A	N/A	86.3 (53.0)	N/A	91.5 (48.9)	N/A	70.6 (20.0)	85.8 (34.6)
**Mean I/sigma(I)**	9.0 (2.8)	9.2 (1.2)	16.4 (1.8)	24.5 (3.6)	10.3 (2.5)	8.5 (1.6)	22.2 (0.7)	12.3 (1.5)	11.2 (1.1)	20.1 (3.6)	7.0 (1.4)
**Wilson B-factor**	22.58	48.16	14.03	59.04	13.78	37.75	22.44	24.28	17.76	11.96	20.13
**R-merge**	0.104 (0.510)	0.121 (1.086)	0.042 (0.846)	0.119 (1.351)	0.059 (0.382)	0.115 (0.763)	0.037 (1.128)	0.132 (1.005)	0.055 (0.553)	0.036 (0.146)	0.299 (0.930)
**CC1/2**	0.997 (0.879)	0.994 (0.4)	0.999 (0.706)	1.00 (0.896)	0.996 (0.815)	0.998 (0.587)	1.00 (0.395)	0.998 (0.664)	0.998 (0.638)	0.999 (0.961)	0.974 (0.349)
**Refinement statistics**											
**Reflections used in refinement**	59481	23795	176660	26482	17416	14262	29936	49729	104472	23675	12517
**Reflections used for R-free**	3052	1212	8753	1330	828	728	1530	2473	5221	1206	615
**R-work**	0.1763	0.1955	0.1499	0.2063	0.1504	0.2144	0.1966	0.1656	0.1615	0.1393	0.2263
**R-free**	0.2036	0.2505	0.172	0.2435	0.1594	0.2554	0.2243	0.1975	0.1823	0.1698	0.2607
**Number of non-hydrogen atoms**	6918	5837	6042	5201	1120	7117	1706	6580	6746	3981	2475
**macromolecules**	6344	5760	5415	5102	982	7014	1522	5886	5781	3477	2435
**ligands**	4	0	0	6	8	30	0	113	70	0	0
**solvent**	570	77	627	93	130	73	184	581	937	504	40
**Protein residues**	874	804	728	695	121	966	204	768	769	454	331
**RMS(bonds)**	0.003	0.006	0.01	0.007	0.012	0.007	0.008	0.007	0.007	0.012	0.004
**RMS(angles)**	0.67	0.82	1.19	0.98	1.22	1.19	1.02	0.94	0.95	1.19	0.74
**Ramachandran favoured (%)**	98.14	96.72	97.77	95.78	100	94.25	98.02	97.51	97.9	96.88	97.23
**Ramachandran allowed (%)**	1.62	3.16	2.23	3.64	0	5.65	1.98	2.49	2.1	3.12	2.77
**Ramachandran outliers (%)**	0.23	0.13	0	0.58	0	0.1	0	0	0	0	0
**Rotamer outliers (%)**	0.45	0.17	0	0.37	0.92	2.23	1.91	0.64	0.65	0	0
**Clashscore**	2.79	9.72	2.88	7.49	3.59	8.48	2.68	4.97	4.19	1.46	4.24
**Average B-factor**	29.78	47.65	20.58	64.91	17.49	32.72	30.62	25.55	21.62	13.23	23.76
**macromolecules**	29.1	47.72	18.55	65.08	15.65	32.64	29.52	24.57	19.9	11.74	23.78
**ligands**	37.39			48.14	19.39	66.33		39.66	26.71		
**solvent**	37.29	43.09	38.13	56.83	31.24	25.94	39.76	32.76	32.09	23.49	22.93
**Number of TLS groups**	1	1		1	1	1	5	1	8	20	1

Statistics for the highest-resolution shell are shown in parentheses.

Supplementary Table S2. Data collection and refinement statistics

**Uniprot ID**	**Gene Name**	**Organism**	**Taxonomy**	**Length (AA)**	**Tip Domain**	**Notes**
M4VS45	A11Q_1788	*Pseudobdellovibrio exovorus* JSS	Oligoflexia	1621	TNF-like	Epibiotic predator
E1X3N0	BMS_2153	*Halobacteriovorax marinus*	Oligoflexia	1915	S74	Marine predator
A0A0B8WIH4	OM95_08955	*Bdellovibrio* sp. ArHS	Oligoflexia	1001	FGE	Thermophilic predator
A0A5S9IRI7	UABAM_04682	*Candidatus Uabimicrobium amorphum*	PVC group	837	S74-like	Endocytosis-like predator
G2KMH7	MICA_2368	*Micavibrio aeruginosavorus* (strain ARL-13)	Alphaproteobacteria	654	S74-like	Unrelated epibiotic predator
A0A3D8MTN3	DV096_14225	Bradymonadaceae bacterium TMQ3	Deltaproteobacteria	434	Beta helix	Predator Grouping
A0A7Y5LMZ2	HUU55_07175	Myxococcales bacterium	Deltaproteobacteria	559	Beta helix	Wolfpack predator Grouping
A0A7Y5J594	HUU01_15465	Saprospiraceae bacterium	FCB group	704	Collagen-like	Ixotrophy predator Grouping
A0A0S2HW82	L21SP5_00641	*Salinivirga cyanobacteriivorans*	FCB group	854	Beta sandwich	Predator of cyanobacteria
A9E803	KAOT1_09059	*Kordia algicida* OT-1	FCB group	592	Beta sandwich	Predator of algae
A0A5J4FZN9	ULMS_06990	*Patiriisocius marinistellae*	FCB group	417	Invasin / intimin	-
A0A239D1M7	SAMN06265376_109101	*Dokdonia pacifica*	FCB group	481	S74-like	-
A0A2H0LB00	COV76_08125	*Candidatus Omnitrophica* bacterium	OP3	713	S74-like	OP3 enrichment of predators and Symbionts (Seymour et al., 2023)

Supplementary Table S3 A selection of diverse MAT proteins from predator-associated lifestyles.

HD100 gene	109J locus tag	109J protein ID	Seq ID
bd0704	EP01_17790	AHZ86772.1	99.85
bd0705	EP01_17795	AHZ86773.1	100
bd0726	EP01_17895	AHZ86793.1	98.98
bd0884	EP01_14555	AHZ86143.1	99.74
bd1130	EP01_15685	AHZ86365.1	96.86
bd1334	EP01_16575	AHZ86538.1	100
bd1641	EP01_04410	AHZ84185.1	98.41
bd1712	EP01_04670	AHZ84236.1	99.49
bd2088	EP01_06465	AHZ84579.1	98.49
bd2133	EP01_06955	AHZ84675.1	98.64
bd2439			
bd2548	EP01_08885	AHZ85052.1	99.76
bd2565	EP01_08955	AHZ85066.1	99.38
bd2582	EP01_09035	AHZ85081.1	98.42
bd2734	EP01_09815	AHZ85231.1	98.45
bd2740	EP01_09840	AHZ85236.1	99.52
bd2872	EP01_10470	AHZ85359.1	100
bd3182	EP01_01205	AHZ83568.1	99.78
bd3266	EP01_01575	AHZ83642.1	99.72
bd3267	EP01_01580	AHZ83643.1	95.16
bd3442	EP01_02400	AHZ83799.1	98.77

Supplementary Table S4. MAT genes in *B. bacteriovorus* strain 109J. Homologous genes to all adhesins found in strain HD100 are shown for strain 109J. All HD100 genes were blasted against the 109J genome using tblastn. Alignments were made using Clustal Omega, with the sequence identity for each shown. Interestingly, although most adhesins have homologues with high sequence identity, *bd2439* is lost in 109J (highlighted red).

Supplementary Table S5- Primers and Plasmids used in this study

Primer	Sequence (5' to 3')	Purpose
bd0635-tag-F	CGGAGCGGTACCGTTTGAAGGGTTTAAAAAAG	Contains six random 5' bases then a EcoRI site that is followed by the bd0635 ORF
bd0635-tag-R	TCACTGGAATTCATGAAGTTGATCATTCTTGT	Contains the 3' region of the bd0635 ORF minus stop codon followed by a KpnI site and six random 3' bases for cutting efficiency
Bd0635upmC_fwd	CGTTGTAAAACGACGGCCAGTGCCAGCCAAGCT GTAAACTGCG	Bd0635-mCherry double crossover fusion<br><br>and<br><br>Bd0635-mTeal double crossover fusion
Bd0635upmC_rev	CCTTGCTCACCATCTTTTTTAAACCCTTCAAACG	
mCherryBd0635_fwd	GGGTTTAAAAAAGATGGTGAGCAAGGGCGAG	
mCherryBd0635_rev	TTCAATCACAGTATTACTTGTACAGCTCGTCCAT	
Bd0635downmC_fwd	GCTGTACAAGTAATACTGTGATTGAAAGAGTTCTC G	
Bd0635downmC_rev	GGAAACAGCTATGACCATGATTACGATCCAAAGCC GACACTTC	
Bd1334mC_F	CGTTGTAAAACGACGGCCAGTGCCAATGGGAAAA CAGTACGTAACG	Bd1334-mCherry single crossover fusion
Bd1334mC_R	CCTTGCTCACCATCCAGCAGGTGAAATTTGTATTG	
1334mCherry_F	TTTCACCTGCTGGATGGTGAGCAAGGGCGAG	
1334mCherry_R	GGAAACAGCTATGACCATGATTACGTTACTTGTAC AGCTCGTCCATG	
Bd2133mN_F	CGTTGTAAAACGACGGCCAGTGCCAATGAGGCC CTTGAACGTAC	Bd2133-mNeon single crossover fusion
Bd2133mN_R	CTTTCGAAACCATTTTGTATCCGGTGATATAAAAG	
2133mNeon_F	ACCGGATACAAAATGGTTTCGAAAGGAGA	
2133mNeon_R	GGAAACAGCTATGACCATGATTACGTCACTTATAG AGTTCATCCATACC	
Bd2439mC_F	CGTTGTAAAACGACGGCCAGTGCCATTGCGTGTT TTAGCTGTACC	Bd2439-mCherry single crossover fusion
Bd2439mC_R	CCTTGCTCACCATGTCCACGCGTTTGATTTG	
2439mCherry_F	CAAACGCGTGGACATGGTGAGCAAGGGCGAG	
2439mCherry_R	GGAAACAGCTATGACCATGATTACGTTACTTGTAC AGCTCGTCCATG	
Bd2734mT_UP_F	CGTTGTAAAACGACGGCCAGTGCCACAGGTTTTA CGTGTGCCAG	Bd2734-mTeal double crossover fusion<br><br>and<br><br>Bd2734-mCherry double crossover fusion
Bd2734mT_UP_R	CCTTGCTCACCATCAGCTTCTTCAAATAGATTTGC	
Bd2734mT_MID_F	TTTGAAGAAGCTGATGGTGAGCAAGGGCGAG	

Bd2734mT_MID_R	CGTATTCAACCAGTTACTTGTACAGCTCGTCCATG	
Bd2734mT_DN_F	GCTGTACAAGTAACTGGTTGAATACGTGGAAAAG	
Bd2734mT_DN_R	GGAAACAGCTATGACCATGATTACGAGACGGCAAAGCGTCCTC	
Bd2740mC_F	CGTTGTAAAACGACGGCCAGTGCCAATGAAACCAAAGCACGTTCGTTATCC	Bd2740-mCherry single crossover fusion
Bd2740mC_R	CCTTGCTCACCATGCGGCGGCCCTTACAGCA	
2740mCherry_F	TAAGGGCCGCCGCATGGTGAGCAAGGGCGAG	
2740mCherry_R	GGAAACAGCTATGACCATGATTACGTTACTTGTACAGCTCGTCCATG	
Bd3182mC_F	CGTTGTAAAACGACGGCCAGTGCCAATGAAAAATCTGATTCCCTCCC	Bd3182-mCherry single crossover fusion
Bd3182mC_R	CCTTGCTCACCATCTGAAGGCCGAGTTTGGC	
3182mCherry_F	ACTCGGCCTTCAGATGGTGAGCAAGGGCGAG	
3182mCherry_R	GGAAACAGCTATGACCATGATTACGTTACTTGTACAGCTCGTCCATG	
Bd3182mC_F	CGTTGTAAAACGACGGCCAGTGCCAATGAAAAATCTGATTCCCTCCC	Bd3182_S782A-mCherry single crossover fusion
Bd3182_S74A_R	TTGAGGCGCGCATCCGCTGCGACAGT	
Bd3182_S74A_F	ACTGTCGCAGCGGATGCGCGCCTCAA	
Bd3182mC_R	CCTTGCTCACCATCTGAAGGCCGAGTTTGGC	
3182mCherry_F	ACTCGGCCTTCAGATGGTGAGCAAGGGCGAG	
3182mCherry_R	GGAAACAGCTATGACCATGATTACGTTACTTGTACAGCTCGTCCATG	
Bd3182mN_F	CGTTGTAAAACGACGGCCAGTGCCAATGAAAAATCTGATTCCCTCCC	Bd3182-mNeon single crossover fusion
Bd3182mN_R	CTTTCGAAACCATCTGAAGGCCGAGTTTGGC	
3182mNeon_F	CTCGGCCTTCAGATGGTTTCGAAAGGAGAG	
3182mNeon_R	GGAAACAGCTATGACCATGATTACGTCACTTATAGAGTTCATCCATACC	
Bd1334_UP_F	CGTTGTAAAACGACGGCCAGTGCCATGATCGTCTTTACGATCTTG	Deletion of *Bd1334*
Bd1334_UP_R	AACATATGCTCCATCCCATGGGATACTTATCG	
Bd1334_DN_F	GTATCCCATGGGATGGAGCATATGTTCGGCC	
Bd1334_DN_R	GGAAACAGCTATGACCATGATTACGTGCCGTGGTTATCTCGCAC	
Bd2133UP_F	CGTTGTAAAACGACGGCCAGTGCCAATCCATGTCGGCCGTGTG	Deletion of *Bd2133*

Bd2133UP_R	GCATTTATTTGTACCTCATGCTGTCCTTTTCGG	
Bd2133DN_F	GGACAGCATGAGGTACAAATAAATGCCTAGTTTCTG	
Bd2133DN_R	GGAAACAGCTATGACCATGATTACGAAGCACGAACACCAAAAG	
Bd2439_UP_F	CGTTGTAAAACGACGGCCAGTGCCATGATCCTGATCACGAACTTC	Deletion of *Bd2439*
Bd2439_UP_R	GCGGGAACCGCATACGCAAGAATGCTGACTTAC	
Bd2439_DN_F	AGCATTCTTGCGTATGCGGTTCCCGCCTATAATG	
Bd2439_DN_R	GGAAACAGCTATGACCATGATTACGATAAAAACATTCCGACGACGTTC	
2734KO_UP_F	CGTTGTAAAACGACGGCCAGTGCCACACCGGAAGGGCTGGGGG	Deletion of *Bd2734*
2734KO_UP_R	CCAGCTACAGCTTGTTCCTCACATGACTTGAAAGCGTATGTTTATTTC	
2734KO_DN_F	TCATGTGAGGAACAAGCTGTAGCTGGTTGAATACGTG	
2734KO_DN_R	GGAAACAGCTATGACCATGATTACGTGACGGCAAAGCGTCCTC	
Bd2740_UP_F	CGTTGTAAAACGACGGCCAGTGCCAAGTCCCTGTGTCCTTGTC	Deletion of *Bd2740*
Bd2740_UP_R	TTAATTAGCGGCGTTTCATTCCACTATATATTCGG	
Bd2740_DN_F	TAGTGGAATGAAACGCCGCTAATTAATAATTTAAAAAC	
Bd2740_DN_R	GGAAACAGCTATGACCATGATTACGAAACTTTTTGAAATTCACAATCAG	
Bd3182UP_F	CGTTGTAAAACGACGGCCAGTGCCACCTGGGTTACGGCAGCCTC	Deletion of *Bd3182*
Bd3182UP_R	GAGATTACTGAAGACGGCAACACCTGACAAAATC	
Bd3182DN_F	CAGGTGTTGCCGTCTTCAGTAATCTCATATCGAAACTG	
Bd3182DN_R	GGAAACAGCTATGACCATGATTACGTTGGGTTTCAAAGGAGGC	

Supplementary Table 5. Plasmids and Primers used in this research study.

Supplementary Table 5A Plasmids

Plasmid	Description	Source
pK18*mobsacB*	Suicide vector (kanR, *lacZ*a, *sacB*) used for crossovers into the *B. bacteriovorus* genome	Schafer *et al.* 1994
pAKF04-mTeal	Template for *mTeal* gene	Fenton *et al.* 2010
pAKF56-mCherry	Template for *mCherry* gene. Cloning vector for Bd0635mCherry	Fenton *et al.* 2010
pAKF220-mNeon	Template for *mNeon* gene	Makowski et al., 2019
pAKF56::bd0635	Bd0635 ORF amplified from B. bacteriovorus HD100 genomic DNA using primers Bd0635tag-F and Bd0635tag-R (minus the stop codon). The PCR product was ligated into pAKF56 cut with EcoRI and KpnI, upstream of the mCherry ORF in the plasmid	This study
p0064-mCh_DXO	Full length Bd0064-mCherry fusion (generates double-crossover, replaces wild type gene in genome) Bd0064 PilZ protein used as a fluorescent cytoplasmic marker	Willis et al., 2016
p0064-mCh_SXO	Full length Bd0064-mCherry fusion (can be added as single-crossover into another genomically tagged strain) Bd0064 PilZ protein used as a fluorescent cytoplasmic marker	Willis et al., 2016
p0064-mC3_DXO	Full length Bd0064-mCerulean3 fusion (generates double-crossover, replaces wild type gene in genome) Bd0064 PilZ protein used as a fluorescent cytoplasmic marker	Raghunathan et al., 2019
pdelta0886	Upstream and downstream fragments around *bd0886* gene to generate unmarked gene deletion	Kuru et al., 2017
pdelta1176	Upstream and downstream fragments around *bd1176* gene to generate unmarked gene deletion	Kuru et al., 2017
pK18::bd0635-mCherry	Bd0635-mCherry fragment from pAKF56:: bd0635 plasmid cloned using EcoRI and XbaI and ligated into pK18mobsacB cut with the same restriction enzymes.	This study
pBd0635-mCh_SXO	Bd0635-mCherry (single crossover)	This study
p0635-mCh_DXO	Full length Bd0635-mCherry fusion (double-crossover	This study
p0635-mT_DXO	Full length Bd0635-mTeal fusion (double-crossover)	This study
p1334-mCh SXO	Full length Bd1334-mCherry fusion (single-crossover	This study
p2133-mN SXO	Full length Bd2133-mNeon fusion (single-crossover	This study
p2439-mCh SXO	Full length Bd2439-mCherry fusion (single-crossover	This study
p2734-mT DXO	Full length Bd2734-mTeal fusion (double-crossover)	This study
p2734-mCh DXO	Full length Bd2734-mCherry fusion (double-crossover)	This study
p2740-mCh SXO	Full length Bd2740-mCherry fusion (single-crossover)	This study
p3182-mCh SXO	Full length Bd3182-mCherry fusion (single-crossover)	This study
P3182_S782A-mCh SXO	Full length Bd3182 with point mutation S782A-mCherry fusion (single-crossover)	This study
p3182-mN SXO	Full length Bd3182-mNeon fusion (single-crossover)	This study

pΔ1334	Upstream and downstream fragments of *bd1334* gene for unmarked gene deletion	This Study
pΔ2133	Upstream and downstream fragments of *bd2133* gene for unmarked gene deletion	This Study
pΔ2439	Upstream and downstream fragments of *bd2439* gene for unmarked gene deletion	This Study
pΔ2734	Upstream and downstream fragments of *bd2734* gene for unmarked gene deletion	This Study
pΔ2740	Upstream and downstream fragments of *bd2740* gene for unmarked gene deletion	This Study
pΔ3182	Upstream and downstream fragments of *bd3182* gene for unmarked gene deletion	This Study

Supplementary Table 5B. Primers used in this research study.

**Primer**	**Sequence (5' to 3')**	**Purpose**
bd0635-tag-F	CGGAGCGGTACCGTTTGAAGGGTTTAAAAAAG	Contains six random 5' bases then a EcoRI site that is followed by the bd0635 ORF
bd0635-tag-R	TCACTGGAATTCATGAAGTTGATCATTCTTGT	Contains the 3' region of the bd0635 ORF minus stop codon followed by a KpnI site and six random 3' bases for cutting efficiency
Bd0635upmC_fwd	CGTTGTAAAACGACGGCCAGTGCCAGCCAAGCTGTAAACTGCG	Bd0635-mCherry double crossover fusion<br><br>and<br><br>Bd0635-mTeal double crossover fusion
Bd0635upmC_rev	CCTTGCTCACCATCTTTTTAAACCCTTCAAACG	
mCherryBd0635_fwd	GGGTTTAAAAAAGATGGTGAGCAAGGGCGAG	
mCherryBd0635_rev	TTCAATCACAGTATTACTTGTACAGCTCGTCCAT	
Bd0635downmC_fwd	GCTGTACAAGTAATACTGTGATTGAAAGAGTTCTCG	
Bd0635downmC_rev	GGAAACAGCTATGACCATGATTACGATCCAAAGCCGACACTTC	
Bd1334mC_F	CGTTGTAAAACGACGGCCAGTGCCAATGGGAAAACAGTACGTAACG	Bd1334-mCherry single crossover fusion
Bd1334mC_R	CCTTGCTCACCATCCAGCAGGTGAAATTTGTATTG	
1334mCherry_F	TTTCACCTGCTGGATGGTGAGCAAGGGCGAG	
1334mCherry_R	GGAAACAGCTATGACCATGATTACGTTACTTGTACAGCTCGTCCATG	
Bd2133mN_F	CGTTGTAAAACGACGGCCAGTGCCAATGAGGCCCTTGAACGTAC	Bd2133-mNeon single crossover fusion
Bd2133mN_R	CTTTCGAAACCATTTTGTATCCGGTGATATAAAAG	
2133mNeon_F	ACCGGATACAAAATGGTTTCGAAAGGAGA	

2133mNeon_R	GGAAACAGCTATGACCATGATTACGTCACTTATAGAGTTCATCCATACC	
Bd2439mC_F	CGTTGTAAAACGACGGCCAGTGCCATTGCGTGTTTTAGCTGTACC	Bd2439-mCherry single crossover fusion
Bd2439mC_R	CCTTGCTCACCATGTCCACGCGTTTGATTTG	
2439mCherry_F	CAAACGCGTGGACATGGTGAGCAAGGGCGAG	
2439mCherry_R	GGAAACAGCTATGACCATGATTACGTTACTTGTACAGCTCGTCCATG	
Bd2734mT_UP_F	CGTTGTAAAACGACGGCCAGTGCCACAGGTTTTACGTGTGCCAG	Bd2734-mTeal double crossover fusion<br><br>and<br><br>Bd2734-mCherry double crossover fusion
Bd2734mT_UP_R	CCTTGCTCACCATCAGCTTCTTCAAATAGATTTGC	
Bd2734mT_MID_F	TTTGAAGAAGCTGATGGTGAGCAAGGGCGAG	
Bd2734mT_MID_R	CGTATTCAACCAGTTACTTGTACAGCTCGTCCATG	
Bd2734mT_DN_F	GCTGTACAAGTAACTGGTTGAATACGTGGAAAAG	
Bd2734mT_DN_R	GGAAACAGCTATGACCATGATTACGAGACGGCAAAGCGTCCTC	
Bd2740mC_F	CGTTGTAAAACGACGGCCAGTGCCAATGAAACCAAAGCACGTTCGTTATCC	Bd2740-mCherry single crossover fusion
Bd2740mC_R	CCTTGCTCACCATGCGGCGGCCCTTACAGCA	
2740mCherry_F	TAAGGGCCGCCGCATGGTGAGCAAGGGCGAG	
2740mCherry_R	GGAAACAGCTATGACCATGATTACGTTACTTGTACAGCTCGTCCATG	
Bd3182mC_F	CGTTGTAAAACGACGGCCAGTGCCAATGAAAAATCTGATTCCCTCCC	Bd3182-mCherry single crossover fusion
Bd3182mC_R	CCTTGCTCACCATCTGAAGGCCGAGTTTGGC	
3182mCherry_F	ACTCGGCCTTCAGATGGTGAGCAAGGGCGAG	
3182mCherry_R	GGAAACAGCTATGACCATGATTACGTTACTTGTACAGCTCGTCCATG	
Bd3182mC_F	CGTTGTAAAACGACGGCCAGTGCCAATGAAAAATCTGATTCCCTCCC	Bd3182_S782A-mCherry single crossover fusion
Bd3182_S74A_R	TTGAGGCGCGCATCCGCTGCGACAGT	
Bd3182_S74A_F	ACTGTCGCAGCGGATGCGCGCCTCAA	
Bd3182mC_R	CCTTGCTCACCATCTGAAGGCCGAGTTTGGC	
3182mCherry_F	ACTCGGCCTTCAGATGGTGAGCAAGGGCGAG	
3182mCherry_R	GGAAACAGCTATGACCATGATTACGTTACTTGTACAGCTCGTCCATG	

Bd3182mN_F	CGTTGTAAAACGACGGCCAGTGCCAATGAAAAATCTGATTCCCTCCC	Bd3182-mNeon single crossover fusion
Bd3182mN_R	CTTTCGAAACCATCTGAAGGCCGAGTTTGGC	
3182mNeon_F	CTCGGCCTTCAGATGGTTTCGAAAGGAGAG	
3182mNeon_R	GGAAACAGCTATGACCATGATTACGTCACTTATAGAGTTCATCCATACC	
Bd1334_UP_F	CGTTGTAAAACGACGGCCAGTGCCATGATCGTCTTTACGATCTTG	Deletion of *Bd1334*
Bd1334_UP_R	AACATATGCTCCATCCCATGGGATACTTATCG	
Bd1334_DN_F	GTATCCCATGGGATGGAGCATATGTTCGGCC	
Bd1334_DN_R	GGAAACAGCTATGACCATGATTACGTGCCGTGGTTATCTCGCAC	
Bd2133UP_F	CGTTGTAAAACGACGGCCAGTGCCAATCCATGTCGGCCGTGTG	Deletion of *Bd2133*
Bd2133UP_R	GCATTTATTTGTACCTCATGCTGTCCTTTTCGG	
Bd2133DN_F	GGACAGCATGAGGTACAAATAAATGCCTAGTTTCTG	
Bd2133DN_R	GGAAACAGCTATGACCATGATTACGAAGCACGAACACCAAAAG	
Bd2439_UP_F	CGTTGTAAAACGACGGCCAGTGCCATGATCCTGATCACGAACTTC	Deletion of *Bd2439*
Bd2439_UP_R	GCGGGAACCGCATACGCAAGAATGCTGACTTAC	
Bd2439_DN_F	AGCATTCTTGCGTATGCGGTTCCCGCCTATAATG	
Bd2439_DN_R	GGAAACAGCTATGACCATGATTACGATAAAAACATTCCGACGACGTTC	
2734KO_UP_F	CGTTGTAAAACGACGGCCAGTGCCACACCGGAAGGGCTGGGGG	Deletion of *Bd2734*
2734KO_UP_R	CCAGCTACAGCTTGTTCCTCACATGACTTGAAAGCGTATGTTTATTTC	
2734KO_DN_F	TCATGTGAGGAACAAGCTGTAGCTGGTTGAATACGTG	
2734KO_DN_R	GGAAACAGCTATGACCATGATTACGTGACGGCAAAGCGTCCTC	
Bd2740_UP_F	CGTTGTAAAACGACGGCCAGTGCCAAGTCCCTGTGTCCTTGTC	Deletion of *Bd2740*
Bd2740_UP_R	TTAATTAGCGGCGTTTCATTCCACTATATATTCGG	
Bd2740_DN_F	TAGTGGAATGAAACGCCGCTAATTAATAATTTAAAAAC	
Bd2740_DN_R	GGAAACAGCTATGACCATGATTACGAAACTTTTTGAAATTCACAATCAG	
Bd3182UP_F	CGTTGTAAAACGACGGCCAGTGCCACCTGGGTTACGGCAGCCTC	Deletion of *Bd3182*
Bd3182UP_R	GAGATTACTGAAGACGGCAACACCTGACAAAATC	

Bd3182DN_F	CAGGTGTTGCCGTCTTCAGTAATCTCATATCGAAACTG	
Bd3182DN_R	GGAAACAGCTATGACCATGATTACGTTGGGTTTCAAAGGAGGC	

Construct	Method	Plasmid	Forward primer	Reverse primer	N-terminal tag
Bd2133 21-1031	Gene synthesis and Restriction cloning 5' NdeI and 3' XhoI	Pet29a			MKYLLPTAAAGLLLLAAQPAMAHHHHHH
Bd2133 662-1031	Restriction cloning 5' NdeI and 3' XhoI	Pcold1	ATTACATATGGAAAACCTGTATTTTCAGGGCAATTCCTCAGGAGCTC	TATACTCGAGTTATTTGTATCCGGTGATATAAAAGTTG	MNHKVHHHHHHIEGRHMENLYFQG
Bd2133 910-1031	Mutagenesis	Pcold1	AGCCGCCACGTGGACAAGC	GAGCCGCCCTGAAAATACAGGTTTTC	MNHKVHHHHHHIEGRHMENLYFQG
Bd2734 691-843	Restriction cloning 5' NcoI and 3' XhoI	Pet26b	AAAACCATGGCGCACCACCATCACCATCATACAAGCACCCTGATGGGG	TATACTCGAGTTATTTGTATCCGGTGATATAAAAGTTG	MKYLLPTAAAGLLLLAAQPAMAMAHHHHHH
Bd1334 818-1151	Restriction cloning 5' XhoI and 3' HindIII	Pcold1	TATACTCGAG GAAAACCTGTATTTTCAGGGCTCTTCGGCTGAAACAG	TATA AAGCTT TTA CCA GCA GGT GAA ATT TGT ATT G	MNHKVHHHHHHIEGRHMELGTLEENLYFQG
Bd1334 914-1151	Restriction cloning 5' XhoI and 3' HindIII	Pcold1	TATACTCGAG GAA AAC CTG TAT TTT CAG GGC AGT GCC GGA ACT CTC G	TATA AAGCTT TTA CCA GCA GGT GAA ATT TGT ATT G	MNHKVHHHHHHIEGRHMELGTLEENLYFQG
Bd2439 837-1107	Restriction cloning 5' BamHI and 3' XhoI	Pet26b	TCTT GGATCC G CAC CAC CAT CAC CAT CAT GGTGGTTTGGATATCGTG	AAAA CTCGAG CTA GTC CAC GCG TTT GAT TTG	MKYLLPTAAAGLLLLAAQPAMAMDIGINSDPHHHHHH
Bd3182 632-922	Restriction cloning 5' NdeI and 3' XhoI	Pcold1	ATTACATATGGAAAACCTGTATTTTCAGGGCGGTACGAACGATGCTTTC	TATA CTCGAG TTACTGAAGGCCGAGTTTGG	MNHKVHHHHHHIEGRHMENLYFQG
Bd3182 632-922 S782A	Mutagesis	Pcold1	CTGCGTGGACTGTCGCAGCTGATGCGCGCCTCAAG	CTTGAGGCGCGCATCAGCTGCGACAGTCCACGCAG	MNHKVHHHHHHIEGRHMENLYFQG
Bd2740	Gene synthesis and Restriction cloning 5' NdeI and 3' XhoI	Pet29a			KYLLPTAAAGLLLLAAQPAMAHHHHHHAS

Supplementary Table S6 Protein Expression primers and vectors.

Supplementary Table 7 Bacterial Strains Used in Microscopy and Phenotypic Studies

**Strains**	**Description**	**Source**
E. coli DH5a	*E. coli* cloning strain (*fhuA2Δ(argF-lacZ)U169 phoA glnV44 Φ80Δ(lacZ)M15 gyrA96 recA1 relA1*)	New England Biolabs (C2987)
E. coli TOP10	*E. coli* cloning strain (F- *mcrA* Δ(*mrr-hsd*RMS-*mcr*BC) Φ80*lac*ZΔM15 Δ *lac*X74 *rec*A1 *ara*D139 Δ(*araleu*)7697 *gal*U *gal*K *rps*L (StrR) *end*A1 *nup*G)	Thermo Fisher (C404003)
E. coli S17-1	*E. coli* strain (thi, pro, hsdR-, hsdM+, recA; integrated plasmid RP4- Tc::Mu-Kn::tn)	Hanahan D., 1983
E. coli S17-1: pZMR100	*E. coli* strain containing the plasmid pZMR100 (kanR)	Rogers M, *et al.*, 1986
B. bacteriovorus HD100	*B. bacteriovorus* Type strain, genome-sequenced, wild-type	Rendulic S, *et al.*, 2004
B. bacteriovorus HD100 Bd0635mCh_DXO	HD100 containing a double-crossover, full length Bd0635- mCherry fusion as the sole copy of the gene	This study
B. bacteriovorus HD100 Bd0635mT_DXO	HD100 containing a double-crossover, full length Bd0635- mTeal fusion as the sole copy of the gene	This study
B. bacteriovorus HD100 Bd2734mT_DXO	HD100 containing a double-crossover, full length Bd2734- mTeal fusion as the sole copy of the gene	This study
B. bacteriovorus HD100 Bd2734mCh_DXO	HD100 containing a double-crossover, full length Bd2734- mCherry fusion as the sole copy of the gene	This study
B. bacteriovorus HD100 Bd0635mCh_SXO	HD100 containing a single-crossover, full length Bd0635- mCherry fusion	This study
B. bacteriovorus HD100 Bd1334mCh_SXO	HD100 containing a single-crossover, full length Bd1334- mCherry fusion	This study
B. bacteriovorus HD100 Bd2439mCh_SXO	HD100 containing a single-crossover, full length Bd2439- mCherry fusion	This study
B. bacteriovorus HD100 Bd2740mCh_SXO	HD100 containing a single-crossover, full length Bd2740- mCherry fusion	This study
B. bacteriovorus HD100 Bd3182mCh_SXO	HD100 containing a single-crossover, full length Bd3182- mCherry fusion	This study
B. bacteriovorus HD100 Bd2133mN_SXO	HD100 containing a single-crossover, full length Bd2133- mNeon fusion	This study
B. bacteriovorus HD100 Bd3182_S782AmCh_SXO	HD100 containing a single-crossover, full length Bd3182 with point mutation S782A- mCherry fusion	This study
B. bacteriovorus HD100 Bd3182mN_SXO	HD100 containing a single-crossover, full length Bd3182- mNeon fusion	This study
B. bacteriovorus HD100 Bd0635mCh_SXO plus Bd2734mT_DXO	HD100 containing a single-crossover, full length Bd0635- mCherry fusion and a double-crossover, full length Bd2734- mTeal fusion	This study
B. bacteriovorus HD100 Bd1334mCh_SXO plus Bd0635mT_DXO	HD100 containing a single-crossover, full length Bd1334- mCherry fusion and a double-crossover, full length Bd0635- mTeal fusion	This study

B. bacteriovorus HD100 Bd2439mCh_SXO plus Bd0635mT_DXO	HD100 containing a single-crossover, full length Bd2439- mCherry fusion and a double-crossover, full length Bd0635- mTeal fusion	This study
B. bacteriovorus HD100 Bd2740mCh_SXO plus Bd0635mT_DXO	HD100 containing a single-crossover, full length Bd2740- mCherry fusion and a double-crossover, full length Bd0635- mTeal fusion	This study
B. bacteriovorus HD100 Bd3182mCh_SXO plus Bd0635mT_DXO	HD100 containing a single-crossover, full length Bd3182- mCherry fusion and a double-crossover, full length Bd0635- mTeal fusion	This study
B. bacteriovorus HD100 Bd2133mN_SXO plus Bd0635mCh_DXO	HD100 containing a single-crossover, full length Bd2133- mNeon fusion and a double-crossover, full length Bd0635- mCherry fusion	This study
B. bacteriovorus HD100 Bd3182mN_SXO plus Bd0635mCh_DXO	HD100 containing a single-crossover, full length Bd3182- mNeon fusion and a double-crossover, full length Bd0635- mCherry fusion	This study
B. bacteriovorus HD100 *Δbd0886* plus *Δbd1176*	*B. bacteriovorus* containing an in-frame unmarked deletions of both *bd0886* and *bd1176*	Kuru et al., 2017
B. bacteriovorus HD100 *Δbd1334*	*B. bacteriovorus* containing an in-frame unmarked deletion of *bd1334*	This study
B. bacteriovorus HD100 *Δbd2133*	*B. bacteriovorus* containing an in-frame unmarked deletion of *bd2133*	This study
B. bacteriovorus HD100 *Δbd2439*	*B. bacteriovorus* containing an in-frame unmarked deletion of *bd2439*	This study
B. bacteriovorus HD100 *Δbd2734*	*B. bacteriovorus* containing an in-frame unmarked deletion of *bd2734*	This study
B. bacteriovorus HD100 *Δbd2740*	*B. bacteriovorus* containing an in-frame unmarked deletion of *bd2740*	This study
B. bacteriovorus HD100 *Δbd3182*	*B. bacteriovorus* containing an in-frame unmarked deletion of *bd3182*	This study
B. bacteriovorus HD100 Bd2734mT_DXO plus Bd0635mCh_SXO	HD100 containing a single-crossover, full length Bd0635- mCherry fusion and a double-crossover, full length Bd2734- mTeal fusion	This study
B. bacteriovorus HD100 Bd2734mT_DXO plus Bd0064mCh_SXO	HD100 containing a single-crossover, full length Bd0064- mCherry fusion and a double-crossover, full length Bd2734- mTeal fusion	This study

References

CHAO, K. L., SHANG, X. R., GREENFIELD, J., LINDEN, S. B., ALREJA, A. B., NELSON, D. C. & HERZBERG, O. 2022. Structure of Escherichia coli O157:H7 bacteriophage CBA120 tailspike protein 4 baseplate anchor and tailspike assembly domains (TSP4-N). Scientific Reports, 12.

CROOKS, G. E., HON, G., CHANDONIA, J. M. & BRENNER, S. E. 2004. WebLogo: A sequence logo generator. Genome Research, 14, 1188-1190.

FENTON AK, KANNA M, WOODS RD, AIZAWA SI, SOCKETT RE. Shadowing the actions of a predator: backlit fluorescent microscopy reveals synchronous nonbinary septation of predatory Bdellovibrio inside prey and exit through discrete bdelloplast pores. J. Bacteriol. 192, 6329-6335 (2010).

HANAHAN D. Studies on transformation of Escherichia coli with plasmids. J. Mol. Biol. 166, 557-580 (1983).

JUMPER, J., EVANS, R., PRITZEL, A., GREEN, T., FIGURNOV, M., RONNEBERGER, O., TUNYASUVUNAKOOL, K., BATES, R., ZIDEK, A., POTAPENKO, A., BRIDGLAND, A., MEYER, C., KOHL, S. A. A., BALLARD, A. J., COWIE, A., ROMERA-PAREDES, B., NIKOLOV, S., JAIN, R., ADLER, J., BACK, T., PETERSEN, S., REIMAN, D., CLANCY, E., ZIELINSKI, M., STEINEGGER, M., PACHOLSKA, M., BERGHAMMER, T., BODENSTEIN, S., SILVER, D., VINYALS, O., SENIOR, A. W., KAVUKCUOGLU, K., KOHLI, P. & HASSABIS, D. 2021. Highly accurate protein structure prediction with AlphaFold. Nature, 596, 583-589.

KURU, E., LAMBERT, C., RITTICHIER, J. et al. Fluorescent D-amino-acids reveal bi-cellular cell wall modifications important for Bdellovibrio bacteriovorus predation. Nat Microbiol 2, 1648–1657 (2017). https://doi.org/10.1038/s41564-017-0029-y

LEGRAND, P., COLLINS, B., BLANGY, S., MURPHY, J., SPINELLI, S., GUTIERREZ, C., RICHET, N., KELLENBERGER, C., DESMYTER, A., MAHONY, J., VAN SINDEREN, D. & CAMBILLAU, C. 2016. The Atomic Structure of the Phage Tuc2009 Baseplate Tripod Suggests that Host Recognition Involves Two Different Carbohydrate Binding Modules. Mbio, 7.

MAKOWSKI Ł, TROJANOWSKI D, TILL R, LAMBERT C, LOWRY R, SOCKETT RE, ZAKRZEWSKA-CZERWIŃSKA J. Dynamics of Chromosome Replication and Its Relationship to Predatory Attack Lifestyles in Bdellovibrio bacteriovorus. Appl Environ Microbiol. 2019 Jul 1;85(14):e00730-19. doi: 10.1128/AEM.00730-19. PMID: 31076424; PMCID: PMC6606864.

MIRDITA, M., SCHUTZE, K., MORIWAKI, Y., HEO, L., OVCHINNIKOV, S. & STEINEGGER, M. 2022. ColabFold: making protein folding accessible to all. Nature Methods, 19, 679-+.

RAGHUNATHAN D, RADFORD PM, GELL C, NEGUS D, MOORE C, TILL R, TIGHE PJ, WHEATLEY SP, MARTINEZ-POMARES L, SOCKETT RE, TYSON J. Engulfment, persistence and fate of Bdellovibrio bacteriovorus predators inside human phagocytic cells informs their future therapeutic potential. Sci Rep. 2019 Mar 12;9(1):4293. doi: 10.1038/s41598-019-40223-3. PMID: 30862785; PMCID: PMC6414686.

RENDULIC S, et al. A predator unmasked: life cycle of Bdellovibrio bacteriovorus from a genomic perspective. Science 303, 689-692 (2004).

ROBERT, X. & GOUET, P. 2014. Deciphering key features in protein structures with the new ENDscript server. Nucleic Acids Res, 42, W320-4.

ROGERS M, EKATERINAKI N, NIMMO E, SHERRATT D. Analysis of Tn7 transposition. Mol Gen Genet 205, 550-556 (1986).

SCHAFER A, TAUCH A, JAGER W, KALINOWSKI J, THIERBACH G, PUHLER A. Small mobilizable multi-purpose cloning vectors derived from the Escherichia coli plasmids pK18 and pK19: selection of defined deletions in the chromosome of Corynebacterium glutamicum. Gene 145, 69-73 (1994).

SEYMOUR, C. O., PALMER, M., BECRAFT, E. D., STEPANAUSKAS, R., FRIEL, A. D., SCHULZ, F., WOYKE, T., ELOE-FADROSH, E., LAI, D. X., JIAO, J. Y., HUA, Z. S., LIU, L., LIAN, Z. H., LI, W. J., CHUVOCHINA, M., FINLEY, B. K., KOCH, B. J., SCHWARTZ, E., DIJKSTRA, P., MOSER, D. P., HUNGATE, B. A. & HEDLUND, B. P. 2023. Hyperactive nanobacteria with host-dependent traits pervade Omnitrophota. Nature Microbiology.

VAN KEMPEN, M., KIM, S., TUMESCHEIT, C., MIRDITA, M., GILCHRIST, C., L,M, SÖDING, J. & STEINEGGER, M. 2022. Foldseek: fast and accurate protein structure search. BioRXiv.

WILLIS AR, MOORE C, MAZON-MOYA M, KROKOWSKI S, LAMBERT C, TILL R, MOSTOWY S, SOCKETT RE. Injections of Predatory Bacteria Work Alongside Host Immune Cells to Treat Shigella Infection in Zebrafish Larvae. Curr Biol. 2016 Dec 19;26(24):3343-3351. doi: 10.1016/j.cub.2016.09.067. Epub 2016 Nov 23. PMID: 27889262; PMCID: PMC5196024.

WISNIEWSKI, J. R., ZOUGMAN, A., NAGARAJ, N. & MANN, M. 2009. Universal sample preparation method for proteome analysis. Nature Methods, 6, 359-U60.

WU, P., ZHEN, X. K., LI, B. W., YU, Q., HUANG, X. C. & SHI, N. 2021. Crystal structure of the MyRF ICA domain with its upstream 13-helical stalk reveals the molecular mechanisms underlying its trimerization and self-cleavage. International Journal of Biological Sciences, 17, 2931-2943.
