# Supplementary figures and images for: Bdellovibrio bacteriovorus uses chimeric fibre proteins to recognize and invade a broad range of bacterial hosts
Source: Nat Microbiol. 2024 Jan 4;9(1):214–27. doi: 10.1038/s41564-023-01552-2 (PMC10769870; doi:10.1038/s41564-023-01552-2)

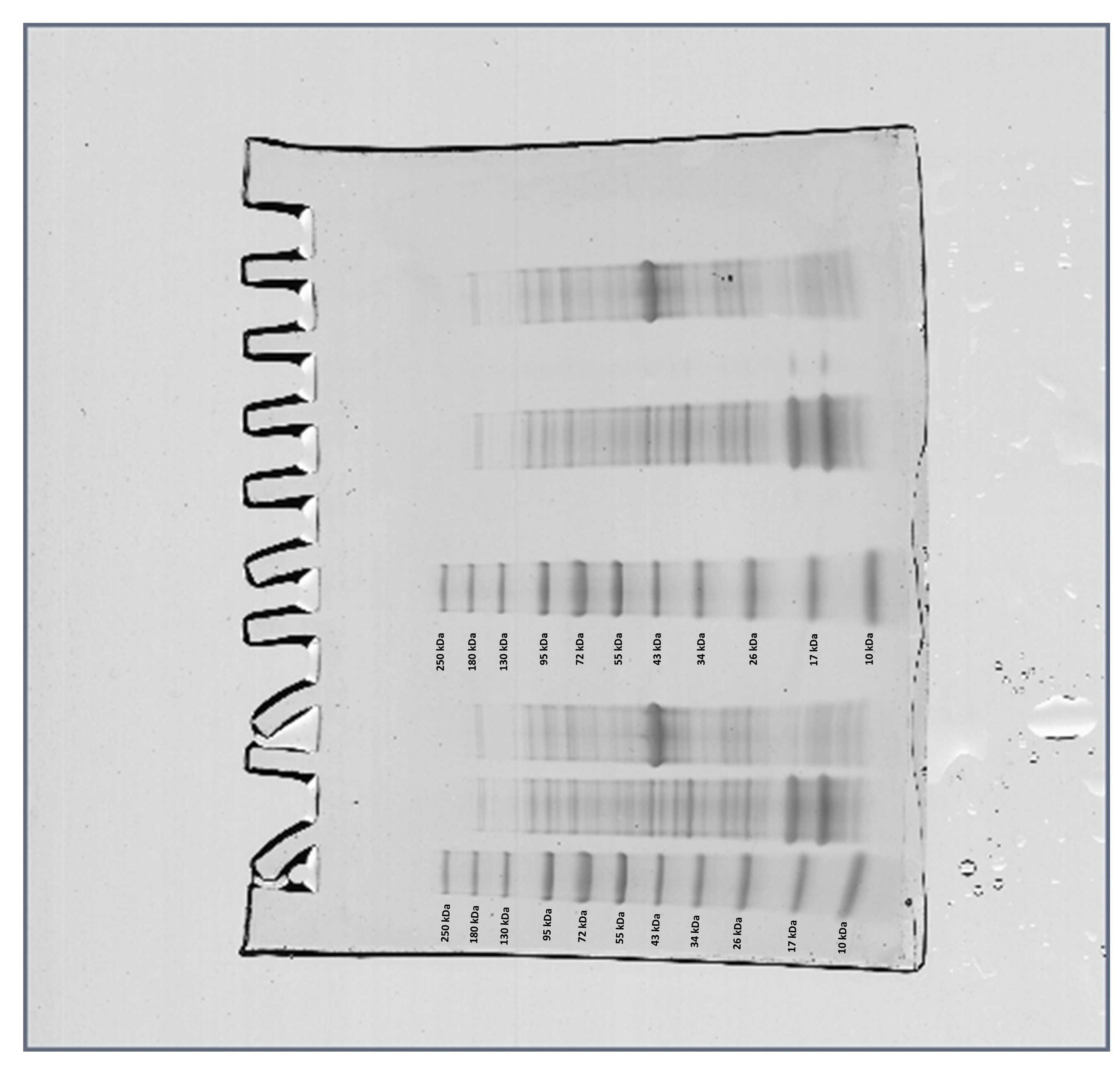

Supplement: Supplementary file 3 — Unprocessed gel for Fig. 4a. [file 41564_2023_1552_MOESM3_ESM.jpeg]
